# Unraveling spatial domain characterization in spatially resolved transcriptomics with robust graph contrastive clustering

**DOI:** 10.1093/bioinformatics/btae451

**Published:** 2024-07-16

**Authors:** Yingxi Zhang, Zhuohan Yu, Ka-Chun Wong, Xiangtao Li

**Affiliations:** School of Artificial Intelligence, Jilin University, Changchun 130012, China; School of Artificial Intelligence, Jilin University, Changchun 130012, China; Department of Computer Science, City University of Hong Kong, Hong Kong 999077, Hong Kong SAR; School of Artificial Intelligence, Jilin University, Changchun 130012, China

## Abstract

**Motivation:**

Spatial transcriptomics can quantify gene expression and its spatial distribution in tissues, thus revealing molecular mechanisms of cellular interactions underlying tissue heterogeneity, tissue regeneration, and spatially localized disease mechanisms. However, existing spatial clustering methods often fail to exploit the full potential of spatial information, resulting in inaccurate identification of spatial domains.

**Results:**

In this article, we develop a deep graph contrastive clustering framework, stDGCC, that accurately uncovers underlying spatial domains via explicitly modeling spatial information and gene expression profiles from spatial transcriptomics data. The stDGCC framework proposes a spatially informed graph node embedding model to preserve the topological information of spots and to learn the informative and discriminative characterization of spatial transcriptomics data through self-supervised contrastive learning. By simultaneously optimizing the contrastive learning loss, reconstruction loss, and Kullback–Leibler divergence loss, stDGCC achieves joint optimization of feature learning and topology structure preservation in an end-to-end manner. We validate the effectiveness of stDGCC on various spatial transcriptomics datasets acquired from different platforms, each with varying spatial resolutions. Our extensive experiments demonstrate the superiority of stDGCC over various state-of-the-art clustering methods in accurately identifying cellular-level biological structures.

**Availability and implementation:**

Code and data are available from https://github.com/TimE9527/stDGCC and https://figshare.com/projects/stDGCC/186525.

## 1 Introduction

Cells are the fundamental components of life, performing diverse functions necessary for the growth and maintenance of living organisms. In multicellular organisms, distinct cell types have specialized functions and collaborate to accomplish specific processes required for the organism’s survival. The functionality of complex biological tissues is inherently linked to the spatial context of the diverse cell types (Asp *et al.* 2020). Therefore, there is a need to carry out transcriptomics on intact tissue where spatial information is preserved. Recent progress in spatial transcriptomics technologies, such as 10× Visium (Ji *et al.* 2020), Slide-seq (Rodriques *et al.* 2019), Slide-seqV2 (Stickels *et al.* 2021), Stereo-seq (Chen *et al.* 2022) and PIXEL-seq (Fu *et al.* 2021), has enabled gene expression analysis in captured locations (referred to as spots) at several cellular resolutions, even reaching the subcellular level, which offers new avenues for comprehending the spatial organization of tissues and the transcriptional patterns of different cell types leading to specific tissue functions. Moreover, spatial transcriptomics (ST) can capture gene expression profiles and spatial information in diseased tissues to gain deeper insights into geographically localized disease mechanisms. Therefore, accurately assigning spots to spatial domains has become a key step in ST analysis.

Unsupervised clustering has been proven to be the most effective method for deciphering spatial domains, as it enables unbiased identification of these domains. Several clustering methods such as k-means (MacQueen *et al.* 1967), Louvain (Blondel *et al.* 2008), Leiden (Traag *et al.* 2019), Seurat (Hao *et al.* 2021), and Scanpy (Wolf *et al.* 2018) have been employed to identify spatial domains in spatially resolved transcriptomics. However, these computational methods primarily rely on gene expression data for clustering, leading to the formation of discontinuous domains due to the exclusion of spatial information. Therefore, it is imperative to develop robust spatial transcriptomic methods that can integrate gene expression data and spatial information to unleash the full potential of ST data for a comprehensive understanding of spatial organization within tissues.

In recent years, there has been a rise in computational methods that incorporate gene expression, spatial location, and morphology to account for the dependence of gene expression on spatial location. These methods have shown considerable improvement in identifying spatial domains by leveraging the spatial information; e.g. BayesSpace (Zhao *et al.* 2021) utilizes a Bayesian statistical model tailored to ST data, which introduces a prior space neighbor structure to promote nearby locations belonging to the same cluster. Similarly, Giotto (Dries *et al.* 2021) unveils spatial domains by employing a hidden Markov random field (HMRF) model to compare the gene expression patterns of each spot with its neighborhood to search for coherent patterns. Another method, DR-SC (Liu *et al.* 2022) simultaneously performs dimension reduction via a probabilistic principal component analysis (PCA) model and promotes spatial clustering using an HMRF based on empirical Bayes. However, these methods are restricted in their performance by their inability to address the complex nonlinear patterns prevalent in gene expression data due to the high-dimensional and sparsity characteristics.

Given these limitations, researchers have turned their attention to developing deep learning-based methods that can effectively capture the nonlinear relationships present in ST data analysis; such as SEDR (Xu *et al.* 2024), stLearn (Pham *et al.* 2023), CoSTA (Xu and McCord 2021), SpaGCN (Hu *et al.* 2021), and STAGATE (Dong and Zhang 2022). These methods leverage diverse neural network architectures to extract the relevant information from the ST data to identify spatial domains. Furthermore, recent advances in unsupervised graph contrastive learning, such as the Deep Graph Infomax (DGI) framework (Velickovic *et al.* 2018) have introduced an alternative objective based on mutual information, and several contrastive learning-based algorithms have been developed such as conST (Zong *et al.* 2022), CCST (Li *et al.* 2022), and SpaceFlow (Ren *et al.* 2022). Although these spatial algorithms have advanced spot clustering compared to non-spatial algorithms, their performance is still limited by the underutilization of spatial information and the inadequate analysis of spot–spot interactions across tissues.

**Figure 1.**
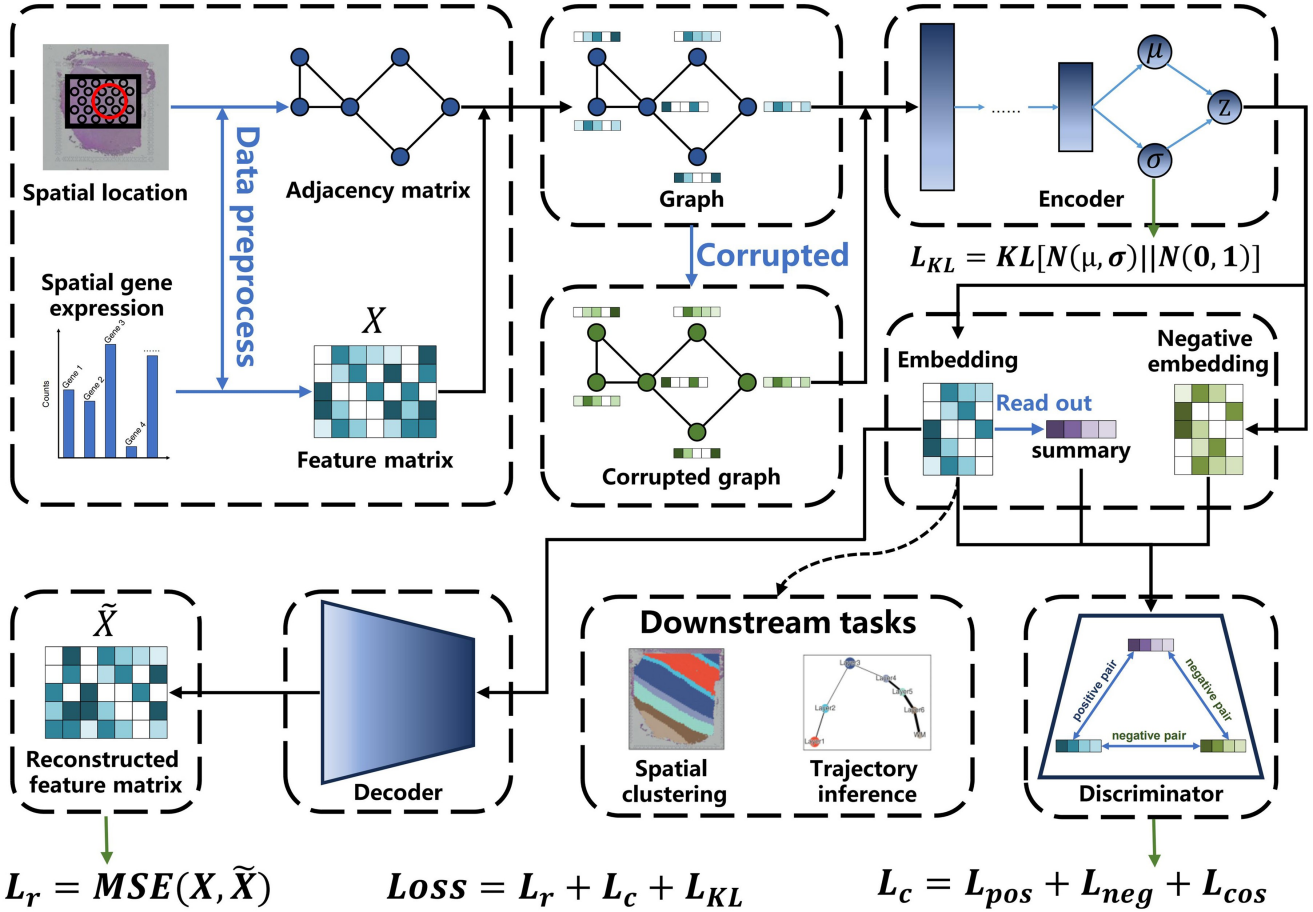
The overview of stDGCC. stDGCC uses an encoder and a decoder to synergistically preserve the spatial and transcriptomic information of spots and constructs positive and negative graphs for self-supervised contrastive learning to refine embedding used for downstream analysis.

Here, we develop a deep graph contrastive clustering framework, named stDGCC, aiming to elucidate accurately the underlying spatial domains by explicitly integrating spatial information and gene expression profiles, as depicted in Fig. 1. stDGCC uses a spatially informed graph node embedding model to extract informative cellular information of the spatial information and gene expression profiles from ST data in an unsupervised manner. Then, by constructing positive and negative graphs, the encoder incorporates both graphs to obtain positive and negative embeddings for contrastive learning, allowing learning of shared semantic representations across different modalities. In addition, three training losses, namely the contrastive learning loss, reconstruction loss, and Kullback–Leibler (KL) divergence loss, are optimized simultaneously to achieve informative and discriminative feature learning while preserving the topological representation of the spot–spot relationships. We carried out spatial domain identification and spatial trajectory inference analyses on a dataset derived from the human dorsolateral prefrontal cortex. The performance evaluation of stDGCC, in comparison with previously proposed algorithms, demonstrates its superior clustering capabilities. Notably, our results highlight the exceptional robustness of stDGCC in accurately identifying the underlying biological structures within the dataset, even under varying resolutions (e.g. 10× Visium, Slide-seq, and Slide-seqV2). Additionally, stDGCC exhibits remarkable discriminatory power in distinguishing populations of cultured cells sharing the same cell type across all four cell cycle phases. This indicates its potential to discern subtle dissimilarities among closely related cells. Furthermore, our comprehensive parameter analysis experiments, along with ablation experiments, offer valuable insights into the intrinsic mechanisms that underlie the efficacy of the proposed model.

## 2 Materials and methods

### 2.1 Data preprocessing

To preprocess the raw gene expression counts, we exclude genes that are expressed in a minimal number of spots (<5 spots) or not expressed at all. Considering the discrete nature of the count matrix and its propensity for high variability in size coefficients, we implement normalization, log-transformation, and scaling techniques to achieve unit variance and zero mean. Subsequently, we select the top *t* highly variable genes to identify genes with high-level information. As a result, we obtain the processed feature matrix $X\in R^{N\times F}$, where *N* denotes the number of spots and *F* represents the number of features.

### 2.2 Spatial graph construction

The spatial information inherent in ST data can be employed to discriminate the states of coexisting similar spots, thereby outlining organizational substructures. To capture the crucial spatial structure in the data, we convert the relative spatial relationship between spots into a topological component of the graph *G*, represented via an undirected adjacency matrix *A*. To accomplish this, we compute the Euclidean distance $d_{ij}$ between ${\text{spot}}_{i}$ and ${\text{spot}}_{j}$ and determine an appropriate threshold δ. On this basis, the adjacent matrix $A_{0}\in R^{N\times N}$ can be defined as follows:

(1) $$\begin{array}{c} A_{0ij}=\{\begin{array}{cc} 1 & ,\;\text{if}\;d_{ij}<\delta \\ 0 & ,\;\text{otherwise}. \end{array} \end{array}$$

Then, to balance the weight of gene expression between a spot and its neighboring spots, we introduce a hyperparameter λ to generate the hybrid adjacency matrix *A* as follows:

(2) $$A=(1-\lambda )*A_{0}+\lambda *I,$$

where $I\in R^{N\times N}$ denotes an identity matrix.

### 2.3 Data augmentation

For subsequent contrastive learning, we employ data augmentation to generate a corrupted graph. Specifically, given the adjacency matrix *A* and the processed feature matrix *X*, we create the corrupted graph by randomly shuffling the feature vectors amongst the spots while maintaining the original graph’s topology. Let $\bar{G}$ and $\bar{X}$ represent the corrupted graph and the shuffled feature matrix, respectively.

### 2.4 Spatially informed graph node embedding

To integrate gene expression profiles and spatial information effectively within the low-dimensional space, we present a novel approach called the variational graph convolutional encoder. This encoder architecture is composed of multiple layers of graph convolutional network (GCN) (Kipf and Welling 2016) and a dedicated layer of variational graph convolutional network (VGCN). The GCN component is responsible for extracting the crucial representations from ST data, capturing the underlying spatial relationships and patterns. On the other hand, the VGCN introduces a disturbance process that involves sampling from a probability distribution. This perturbation aims to enhance the network’s ability to learn robust and generalized representations that can then better capture the complexity and variability of the data. By combining with VGCN, a disturbance process, our proposed model can overcome overfitting and learn more informative and adaptable representations. This approach enables the network to effectively combine gene expression profiles and spatial information, resulting in improved performance and better insights into the underlying biological processes.

In our model, the initial three layers of the encoder comprise GCNs that aim to learn a set of filters to be applied to the node features in a graph. The learned filters are intended to create new, more informative representations of the nodes. Given the preprocessed feature matrix *X* and adjacency matrix *A*, the *k*-th layer of the GCN is defined as follows:

(3) $$\begin{array}{c} \begin{array}{c} E^{k+1}=GCN^{k}(E^{k},A)\\ ={\tilde{D}}^{-1/2}A{\tilde{D}}^{-1/2}E^{k}W^{k}+b^{k}, \end{array} \end{array}$$

where $E^{k}$ and $E^{k+1}$ represent the input and output of the *k*-th graph convolutional layer, respectively; $W^{k}$ and $b^{k}$ denote the trainable weight matrix and bias vector used for feature transformation; additionally, $\tilde{D}$ is a diagonal matrix with diagonal elements defined as ${\tilde{D}}_{ii}=\sum_{j}A_{ij}$.

Then, to further capture important features from the gene expression profiles and the spatial information, and to mitigate the impact of noisy data, we employ VGCN in the final layer.

This layer consists of two GCN layers that learn the parameters μ and σ in parallel, as described in the following equations.

(4) $$\mu ={\text{GCN}}_{\mu }(E,A)$$

(5) $$\sigma ={\text{GCN}}_{\sigma }(E,A),$$

where *E* denotes the output of the graph convolutional layer, and the matrices $\mu \in R^{N\times M}$ and $\sigma \in R^{N\times M}$ are generated by GCN. Here, *N* represents the number of spots, while *M* denotes the dimensionality of the feature space for each spot. Each ${\text{spot}}_{i}$ generates respective vectors $\mu_{i}\in R^{M}$ and $\sigma_{i}\in R^{M}$ comprising *M* means and standard deviations. Subsequently, to obtain the latent representation $Z_{i}\in R^{M}$ for each ${\text{spot}}_{i}$, we perform sampling based on the distributions defined by $\mu_{i}$ and $\sigma_{i}$. The equation governing this process is:

(6) $$Z_{i}∼N(\mu_{i},\text{diag}(\sigma_{i}^{2})).$$

In our implementation, we employ a reparameterization trick to generate the realization of $Z_{i}$ as follows:

(7) $$\epsilon_{i}∼N(0,I)$$

(8) $$Z_{i}=\mu_{i}+\sigma_{i}⊙\epsilon_{i}.$$

This reparameterization trick enables back-propagation in the model. Ultimately, amalgamating all extracted vectors $Z_{i}$ yields the matrix $Z=\{Z_{1},Z_{2},\ldots ,Z_{N}\}$, from which we conclude that Z=μ+σ⊙ϵ. Finally, *Z* is input into the PReLU activation function.

(9) H=PReLU(Z),

where $H\in R^{N\times M}$ represents the output of the encoder. Afterward, to ensure the normal distribution of latent variable $Z_{i}$, the KL divergence is used to measure the difference between the two probability distributions to encourage the learned latent variable to be closer to a prior distribution that is assumed as a Gaussian prior $p(Z_{i})=N(Z_{i}|0,I)$. Therefore, we define the KL divergence loss function of the encoder as follows:

(10) $$\begin{array}{c} \begin{array}{cc} L_{\text{KL}} & =\frac{1}{N}\sum_{i}^{N}KL[q(Z_{i}|X,A)\|p(Z_{i})]\\ & =\frac{1}{N}\frac{1}{2}\sum_{i}^{N}\sum_{j}^{M}\left(-\text{log}\;\sigma_{ij}^{2}+\mu_{ij}^{2}+\sigma_{ij}^{2}-1\right). \end{array} \end{array}$$

### 2.5 Spatial and transcriptomic information fusion decoder

The previously described encoder essentially fuses and compresses ST data into a smaller representation. To ensure that the embedding contains the essential information needed to reconstruct the feature matrix and to avoid over-convolution of the graph convolution layer, we use a decoder consisting of fully connected layers symmetric to the encoder. The decoder can be defined as follows:

(11) $$\tilde{X}=f_{D}(H),$$

where $\tilde{X}\in R^{N\times F}$ denotes the reconstructed feature matrix. To measure the difference between the reconstructed feature matrix and the original feature matrix, we define the reconstruction loss function of the decoder as follows:

(12) $$\begin{array}{c} L_{r}=\frac{1}{N}{\left(X-\tilde{X}\right)}_{2}^{2}. \end{array}$$

### 2.6 Self-supervised contrastive learning for embedding refinement

To enhance the informativeness and discriminative power of the embedding *H*, we employ a self-supervised contrastive learning strategy, which ensures that the model can efficiently capture the local spatial context of the spots. To generate dissimilar examples, we apply data augmentation to create a corrupted graph. In addition, using both the original and corrupted graphs as inputs, the encoder $f_{E}$ generates two corresponding representation matrices $H\in R^{N\times M}$ and $\bar{H}\in R^{N\times M}$ for the spots, as below:

(13) $$H=f_{E}(X,A)$$

(14) $$\bar{H}=f_{E}(\bar{X},A).$$

To further complement the comparative learning strategy, we use a readout function *S* such that s=S(H) to obtain the global representation $s\in R^{M}$ by averaging over the node embeddings *H*. For ${\text{spot}}_{i}$ in the original graph, its representation $h_{i}$ along with the global representation *s* forms a positive pair. In contrast, the corresponding representation $\bar{h_{i}}$ from the corrupted graph, along with the global representation *s*, forms a negative pair. On this basis, the self-supervised contrastive learning approach aims to train the model to distinguish between positive and negative pairs. To achieve this, we introduced a bilinear layer as discriminator $D:R^{M}\times R^{M}\to R$ to assess the amount of mutual information contained within each pair. The loss function for the positive and negative pairs is defined as follows:

(15) $$L_{\text{pos}}=-\frac{1}{N}\sum_{i}^{N}l\text{og}D(h_{i},s)$$

(16) $$L_{\text{neg}}=-\frac{1}{N}\sum_{i}^{N}l\text{og}(1-D(\bar{h_{i}},s)).$$

Additionally, we employ cosine similarity to maximize the separation between positive and negative embeddings (negative pairs) in the latent space during the contrastive learning process. This ensures that positive pairs are brought closer together, while negative pairs are pushed further apart, facilitating the discrimination between them:

(17) $$L_{\;\text{cos}\;}=-\frac{1}{N}\sum_{i}^{N}l\text{og}(1-\text{cos}(h_{i},\bar{h_{i}})).$$

After obtaining the above loss functions, the loss function of contrastive learning $L_{c}$ is as follows:

(18) $$L_{c}=L_{\text{pos}}+L_{\text{neg}}+L_{\;\text{cos}\;}.$$

### 2.7 Spatial domain assignment via joint embedding optimization and clustering

To improve the generalization performance of the model, we jointly optimize the individual loss functions throughout the training process. The total objective function is a linear combination of the following loss functions:

(19) $$L=\alpha *L_{c}+\beta *L_{r}+\gamma *L_{\text{KL}},$$

where $L_{c}$ represents the self-supervised contrastive learning loss, $L_{r}$ represents the reconstruction loss, and $L_{\text{KL}}$ represents the KL divergence loss. The coefficients α, β, and γ are weight coefficients that control the balance of the total objective function. After training the model, we perform PCA on the node embeddings *H* to obtain the first 30 principal components. Subsequently, we apply the K-means clustering algorithm to identify spatial domains.

### 2.8 Implementation details

In our study, we configured the GCN with three layers of node sizes: 1024, 512, and 256, respectively. The VGCN was set to have 128 nodes. The decoder consisted of four fully connected layers with node sizes of 128, 256, 512, and 1024. For the DLPFC dataset, the construction of the spatial graph involved setting the parameter λ to 0.8 and δ to 250. The weight coefficients for the objective function (α,β,γ) were, respectively, set to (2.0,0.05,0.005). During the training phase, we employed the Adam algorithm with a learning rate of 1e−5, and the training process spanned 2500 epochs. The parameters of the baseline methods were set exactly as in the original publications. All experiments were conducted on an Ubuntu server equipped with an NVIDIA GTX 2080Ti GPU, which provided 24 GB of memory. The runtime of stDGCC is shown in Supplementary Fig. S1.

## 3 Results

### 3.1 Data sources

To evaluate the effectiveness of our proposed stDGCC, eight distinct kinds of spatial transcriptomics datasets were employed (see Supplementary Table S1 for details). The first is the human dorsolateral prefrontal cortex (DLPFC) dataset (Maynard *et al.* 2021), which consists of 12 tissue sections acquired using 10× Visium technology. The number of spots in each section ranged from 3460 to 4789, all with a gene number of 33 538. Each section was manually annotated to contain five to seven regions, including the DLPFC layers and white matter. The second dataset is a mouse hippocampus dataset (Stickels *et al.* 2021) from Slide-seqV2 technology comprising 20 143 spots and 19 653 genes, which is not annotated. The third dataset is a mouse hippocampus (Rodriques *et al.* 2019) dataset profiled by Slide-seq with 18 508 spots and 17 275 genes. The fourth dataset is a mouse brain dataset (Bae *et al.* 2023) with 2702 spots and 32 285 genes, which is collected from the 10× Genomics website. The fifth dataset is the Xenium mouse brain dataset, featuring 162 033 cells and 541 genes, sourced from the 10× Genomics website (https://www.10xgenomics.com/datasets/fresh-frozen-mouse-brain-replicates-1-standard). The sixth dataset is the CosMx mouse brain dataset generated using the CosMx Mouse Neuroscience RNA panel, comprising 38 996 cells and 950 genes (https://nanostring.com/). The seventh dataset is a MERFISH mouse brain dataset from Vizgen with 83 546 cells and 483 genes (https://vizgen.com/data-release-program/). The last dataset is the MERFISH dataset (Xia *et al.* 2019), consisting of the expression of 10 050 genes in 1368 human osteosarcoma cells from 3 batches (replicates). As mentioned by Xia *et al.* (2019), obvious spatial structures of the cell cycle phase were discovered within this cell population, so it represents an ideal spatial dataset to test clustered cell groups since the cell cycle can be used as ground truth here.

### 3.2 Baseline methods

We conducted a comprehensive comparative analysis of stDGCC against three base clustering methods that do not utilize neural networks and several state-of-the-art ST data clustering methods including five deep embedded clustering methods and three deep contrastive learning methods.

For the base clustering methods, Seurat (Hao *et al.* 2021) is a widely-used library for single-cell transcriptomics analysis that has also been adapted to accommodate ST data. BASS (Li and Zhou 2022) performs multiscale transcriptomic analyses in the form of joint cell type clustering and spatial domain detection, with the two analytic tasks carried out simultaneously within a Bayesian hierarchical modeling framework. SpatialPCA (Shang and Zhou 2022) builds upon the probabilistic version of PCA, incorporates localization information as additional input, and uses a kernel matrix to explicitly model the spatial correlation structure across tissue locations. In the category of deep embedded clustering methods, SEDR (Xu *et al.* 2024) employs a deep autoencoder network to learn gene representations and uses a variational graph autoencoder to simultaneously embed spatial information. stLearn (Pham *et al.* 2023) uses spatial information and morphological distance to normalize gene expression data and performs unsupervised clustering. SpaGCN (Hu *et al.* 2021) applies the GCN to integrate gene expression, spatial location, and histology and adds an iterative clustering layer to identify spatial domains. STAGATE (Dong and Zhang 2022) uses a graph attention autoencoder framework to identify spatial domains by integrating spatial information and gene expression profiles. DeepST (Xu *et al.* 2022) extracts feature vectors from morphological image tiles using a pretrained deep neural network model then integrates the extracted features with gene expression and spatial location data to characterize the correlation of spatially adjacent spots, and creates a spatial augment gene expression matrix. For deep contrastive learning methods, CCST (Li *et al.* 2022) utilizes DGI to learn embeddings that encapsulate spatial structure and gene expression information. SpaceFlow (Ren *et al.* 2022) utilizes a DGI framework in combination with spatial regularization techniques to extract low-dimensional embeddings that exhibit spatial consistency. Lastly, conST (Zong *et al.* 2022) introduces a two-stage contrastive learning framework designed to generate concise and informative low-dimensional embeddings.

### 3.3 Benchmarking metrics for stDGCC clustering

In this article, we employed two widely used evaluation measures, normalized mutual information (NMI) and adjusted rand index (ARI), to estimate the performance of the different computational methods (Su *et al.* 2024).

NMI measures the normalized mutual information between predicted labels and true labels, resulting in a value ranging from 0 to 1. A higher NMI value indicates a higher similarity between the two clustering results, while a lower value indicates a lower similarity. ARI evaluates the similarity of two clustering results in a statistical way, resulting in a value ranging from −1 to 1, with a value closer to 1 indicating a higher consistency between the clustering results and the reference labels, a value closer to −1 indicating a lower consistency, and a value of 0 indicating random clustering. Let *U* denote the predicted cluster labels and *V* denote the ground-truth labels. The NMI is defined as:

(20) $$\text{NMI}(U,V)=\frac{-2\sum_{ij}\left(n_{ij}\;\text{log}\;\left(\frac{N\cdot n_{ij}}{a_{i}\cdot b_{j}}\right)\right)}{\sum_{i}\left(a_{i}\;\text{log}\;\left(\frac{a_{i}}{N}\right)\right)+\sum_{j}\left(b_{j}\;\text{log}\;\left(\frac{b_{j}}{N}\right)\right)}.$$

The ARI can be defined as:

(21) $$\text{ARI}(U,V)=\frac{\sum_{ij}\left(\begin{array}{c} n_{ij}\\ 2 \end{array}\right)-\left[\sum_{i}\left(\begin{array}{c} a_{i}\\ 2 \end{array}\right)\sum_{j}\left(\begin{array}{c} b_{j}\\ 2 \end{array}\right)\right]/\left(\begin{array}{c} N\\ 2 \end{array}\right)}{\frac{1}{2}\left[\sum_{i}\left(\begin{array}{c} a_{i}\\ 2 \end{array}\right)+\sum_{j}\left(\begin{array}{c} b_{j}\\ 2 \end{array}\right)\right]-\left[\sum_{i}\left(\begin{array}{c} a_{i}\\ 2 \end{array}\right)\sum_{j}\left(\begin{array}{c} b_{j}\\ 2 \end{array}\right)\right]/\left(\begin{array}{c} N\\ 2 \end{array}\right)},$$

where $a_{i}$ is the number of data objects in the *i*-th cluster of *U*, $b_{j}$ is the number of data objects in the *j*-th cluster of *V*, $n_{ij}$ is the number of data objects that belong to both cluster *i* in *U* and cluster *j* in *V*, and *N* denotes total sample size (Fan *et al.* 2023).

### 3.4 stDGCC improves the identification of known layers on the human dorsolateral prefrontal cortex dataset

To quantitatively evaluate the spatial clustering performance of stDGCC, we first applied it to a 10× Visium dataset of 12 human DLPFC sections. Each section was manually annotated to contain five to seven regions, including the DLPFC layers and white matter. Considering it as the ground truth, we compared the clustering accuracy of stDGCC with other baseline methods in terms of ARI and NMI.

**Figure 2.**
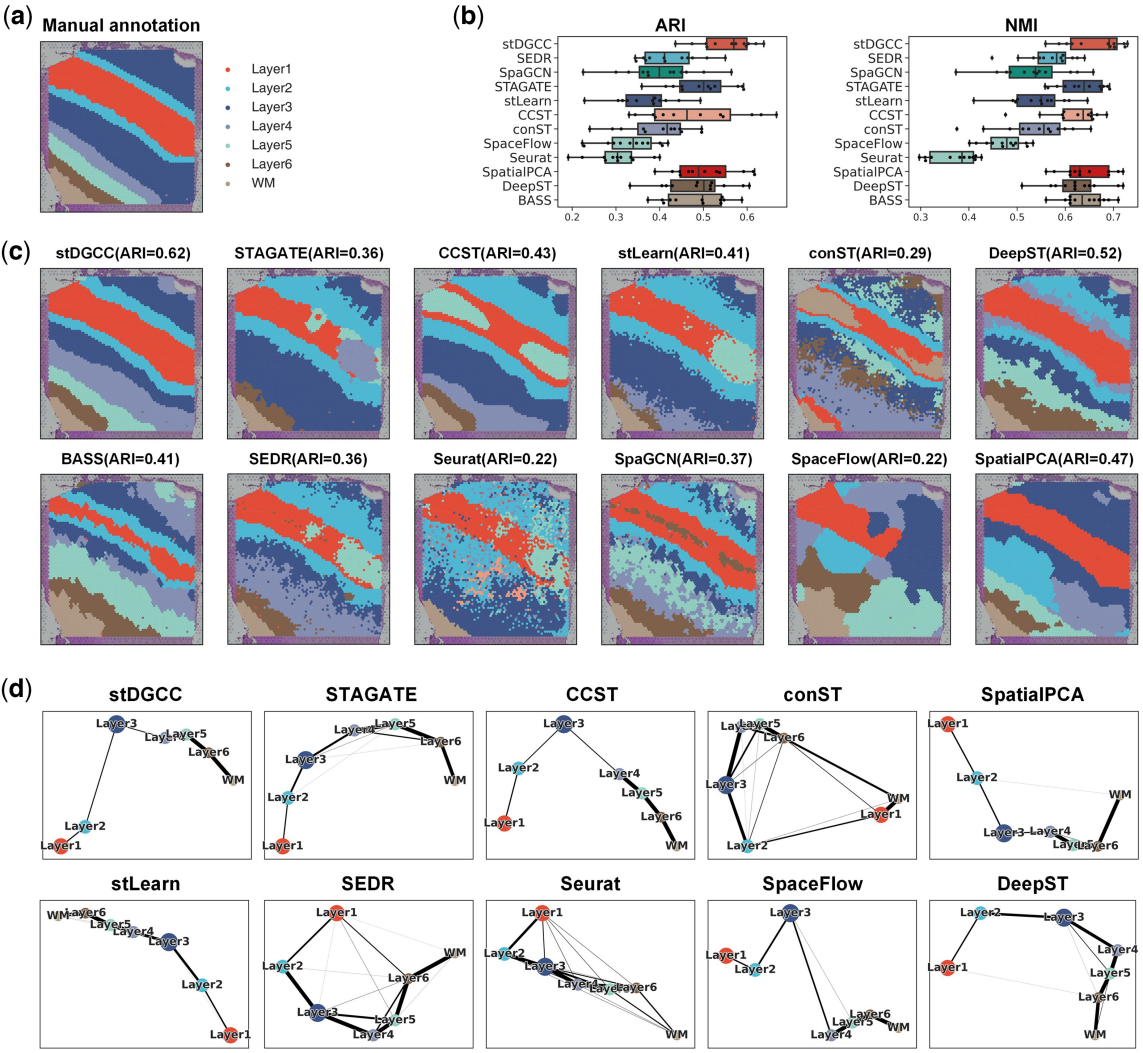
(a) Ground-truth segmentation of cortical layers and white matter in the DLPFC section 151509. (b) Box plots of ARI and NMI for stDGCC and other baseline methods on the DLPFC dataset. (c) Cluster assignments generated by stDGCC and other baseline methods on DLPFC section 151509. (d) PAGA graphs generated by stDGCC and other algorithm embeddings on DLPFC section 151509.

Supplementary Table S2 presents the clustering performance of stDGCC and other baseline methods on the DLPFC dataset where the best results are marked in bold. From the results, stDGCC achieved the highest ARI and NMI on 6 of 12 sections. It is worth noting that the median ARI and NMI of stDGCC were significantly higher than the other baseline methods as shown in Fig. 2b. Compared to the deep embedded clustering methods, our proposed stDGCC has a higher average ARI (0.554) on the DLPFC dataset than SEDR (0.422), SpaGCN (0.4), STAGATE (0.493), stLearn (0.369), and DeepST (0.487), demonstrating that the other deep embedding clustering methods may miss key patterns of the node embedding. Also, as methods without the use of neural networks, SpatialPCA (0.504) and BASS (0.484) achieved the second and fifth highest average ARI, respectively. Our observations revealed the inferior performance of the non-spatial method, Seurat, compared to the spatial methods. Seurat consistently demonstrated subpar results across nearly all sections, exhibiting significantly lower median than the spatial method counterparts. This disparity may be attributed to the insufficiency of gene expression profiles alone for the precise identification of spatial domains, highlighting the necessity of integrating additional spatial information. These observations emphasize the superiority of the stDGCC approach.

Figure 2a and c depicts in a visual manner the outcomes of the manual annotation and each method on section 151509. The annotations reveal a distinct transition from layer 1 to layer 3 in both directions. Among all methods, only stDGCC accurately identified the transition, exhibiting smooth and well-defined boundaries in the individual segmentation results. Additionally, we validated the inferred trajectories using the PAGA algorithm. Note that SpaGCN and BASS being end-to-end clustering methods, cannot be visualized using PAGA. As seen in Fig. 2d, both stDGCC- and CCST-embedded PAGA plots depict a linear developmental trajectory from layer 1 to layer 6 and display similarities between adjacent layers. Conversely, the STAGATE-, stLearn-, SpatialPCA-, DeepST-, and SpaceFlow-embedded PAGA results exhibit a limited number of anomalous links, whereas the PAGA results from Seurat, SEDR, and conST demonstrate a significant number of mixed anomalous links. Clustering results for the other sections are in Supplementary Figs S5 and S6. In addition, for a more comprehensive comparison, we also used fixed parameters among all datasets. As shown in Supplementary Fig. S36a, stDGCC still has an advantage over other baseline methods on the DLPFC dataset at fixed parameters.

### 3.5 Parameter analysis

Our study meticulously explored the influence of three critical hyperparameters: λ, δ, and the number of highly variable genes, on our model’s performance. The parameter λ determined the weighting of individual nodes relative to their neighbors in the convolution process. The δ parameter was crucial for establishing the density of edges within the spatial graph, indicating the magnitude of neighboring nodes. Furthermore, the number of highly variable genes directly influenced the amount of effective information and noise present within the ST data. Before performing hyperparameter optimization, we synthesized the hyperparameter settings from the existing literature on previous studies (Li *et al.* 2022; Dong and Zhang 2022) to establish our parameter range. Subsequently, we performed a detailed hyperparameter search on the DLPFC dataset. For the DLPFC dataset, λ, δ, and the number of highly variable genes were varied within specified ranges to assess their impact systematically. Specifically, λ was tested across [0.1, 0.2, 0.3, 0.4, 0.5, 0.6, 0.7, 0.8, 0.9], δ across [50, 100, 150, 250, 350, 400], and the number of highly variable genes across [2000, 3000, 4000, 5000, 6000]. To ensure a comprehensive evaluation of the performance, we exhaustively enumerated these hyperparameter combinations, resulting in a total of 270 distinct hyperparameter configurations, enabling a detailed comparison of clustering performances. Supplementary Figure S7 elucidates the stDGCC model’s efficacy on the DLPFC datasets under these varied settings, revealing discernible trends correlating parameter values with model performance. Significant performance degradation was noted when δ values were at the lower thresholds of 50 and 100, primarily due to the insufficient number of neighboring nodes, which compromises the model’s structural capture capability. Moreover, elevating the count of highly variable genes above 4000 detrimentally introduced noise, thus deteriorating model performance and emphasizing the critical importance of optimal gene selection. While variations in λ did influence outcomes, their effect was relatively marginal in comparison. Based on these observations from Supplementary Fig. S7, we can find that λ=0.8, δ=250, and 3000 highly variable genes can provide the best performance in our main analysis for the DLPFC datasets. The results of the hyperparametric analyses for the other datasets are in Supplementary Figs S8–S24.

In addition to analyses of hyperparameters, we experimentally explored the impact of varying the number of principal components and the number of layers and nodes in our proposed stDGCC model. We delineated our model variants into four configurations: stDGCC1, stDGCC2, stDGCC3, and stDGCC4 (our proposed stDGCC), correlating to the encoder layers, specifically layer one, layer two, layer three, and layer four, with corresponding neuron node configurations of [128], [256–128], [512–256-128], and [1028–512-256–128], respectively. The decoder architecture was designed to be symmetrical to the encoder. In exploring the principal component analysis, our assessments spanned 10, 20, 30, 60, and 90 components, with an additional scenario excluding PCA (utilizing 128 components directly). Our systematic evaluation encompassed 24 distinct model configurations, benchmarking their clustering effectiveness on the DLPFC dataset. As shown in Supplementary Fig. S25, the number of principal components between 20 and 128 has a relatively minor impact on the model’s performance. However, a reduction in the number of layers and nodes is associated with a notable decrease in the model’s overall effectiveness. Specifically, the stDGCC model, when configured to employ the top 30 principal components, attains the highest average metrics on the DLPFC dataset, with an ARI of 0.554 and an NMI of 0.663. This configuration not only ensures superior clustering results but also contributes to a reduction in computational time required for the subsequent analysis.

Finally, we also analyzed the weights of the objective functions of the different modules. In this article, the weight coefficients were chosen (after some preliminary experiments) to give robust results. We collected and summarized different weight coefficients from previous studies (Li *et al.* 2022; Yu *et al.* 2023). On this basis, α, β, and γ were selected from [1.0, 2.0, 3.0, 4.0], [0.02, 0.05, 0.1], and [0.005, 0.015, 0.025], respectively. Then, to provide comprehensive performance evaluations, we enumerated them to obtain 36 distinct loss weight assignments and compared the clustering performance on the DLPFC dataset in those scenarios. From Supplementary Fig. S26, we can observe that variation in weight loss over a certain range has little effect on the clustering performance of stDGCC in most datasets. For the weights of $L_{c}$, α changes in it also cause fluctuations in the model performance. For instance, with β and γ held constant at 0.05 and 0.005, respectively, increasing α from 1.0 to 2.0 enhances the average ARI from 0.517 to 0.554. Further increasing α to 4.0 results in a slight decrease in average ARI to 0.549. Optimal clustering performance, measured by both the average NMI and ARI, was observed when α, β, and γ were set to [2.0, 0.05, 0.005] out of the 36 tested configurations.

### 3.6 Ablation study

To discern the individual contributions of stDGCC’s components, we conducted an ablation study in three different cases: (i) No VGCN, replaced by a general GCN. The total loss function comprised the reconstruction loss and contrastive learning loss. (ii) No fully connected layer-based decoder. The total loss function included the KL loss and the contrastive learning loss. (iii) No contrastive learning (without $L_{c}$). The total loss function consisted of the reconstruction loss and KL loss. Supplementary Figure S27 presents the average NMI and ARI values on the DLPFC dataset of the three cases of altered stDGCC. As shown in the figure, the absence of the contrastive learning component notably reduces the average ARI and NMI of stDGCC from 0.554 to 0.501 and from 0.663 to 0.636, respectively. The results highlight the substantial role of both the decoder and contrastive learning components in enhancing clustering performance, with the KL component also significantly affecting the outcomes, emphasizing the importance of embedding regularization through KL constraints. In summary, we can conclude that all components of the stDGCC method contribute to its effective performance.

### 3.7 stDGCC enables the identification of tissue structures from ST data of different spatial resolutions

**Figure 3.**
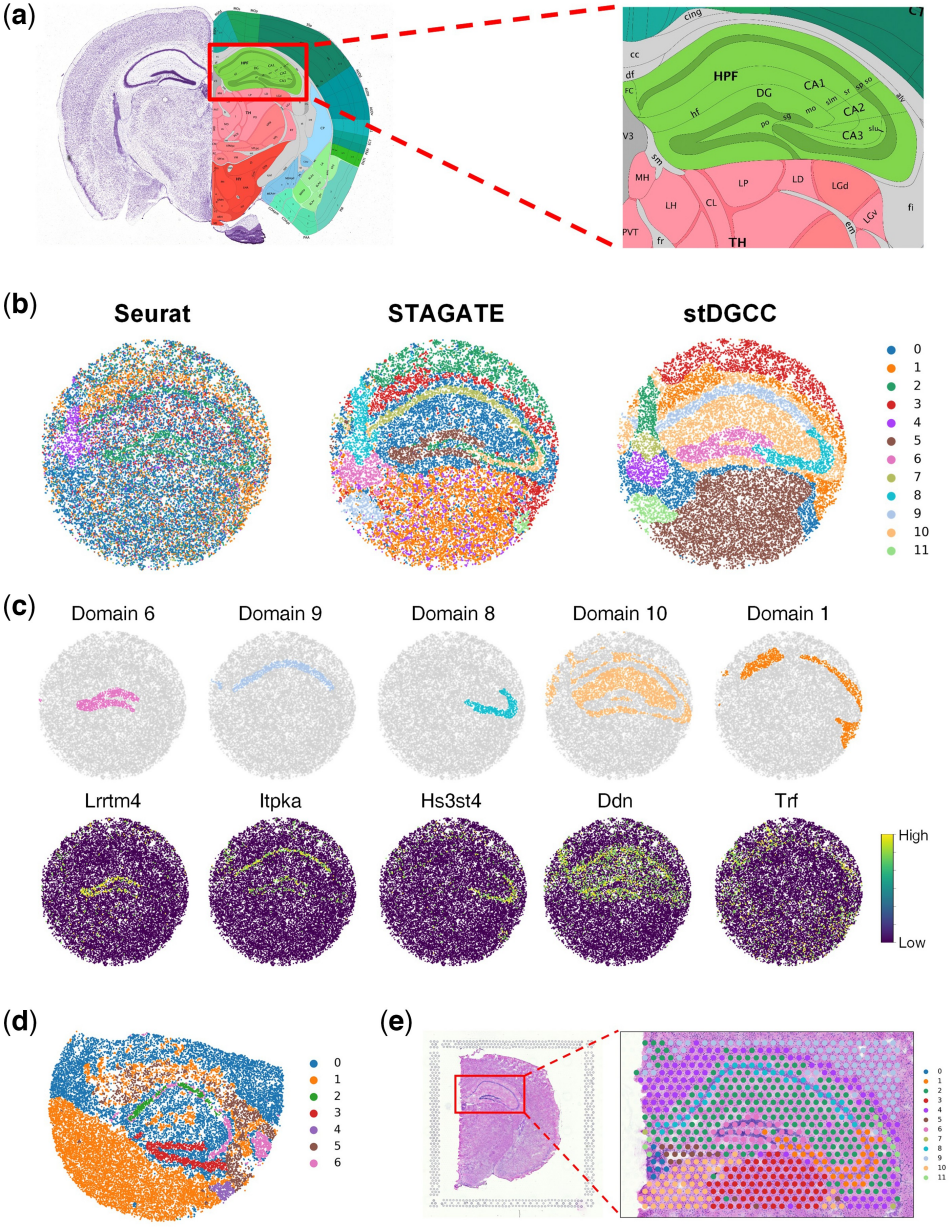
(a) Allen mouse brain reference atlas. (b) Spatial domains generated by Seurat, STAGATE, and stDGCC on the Slide-seqV2 mouse hippocampus dataset. (c) Visualization of marker genes and corresponding domains delineated by stDGCC. (d) Spatial domains generated by stDGCC on the mouse hippocampus dataset profiled by Slide-seq. (e) Spatial domains generated by stDGCC on the mouse brain dataset profiled by 10× Visium.

We further tested whether stDGCC can be applied to ST data of different spatial resolutions (Supplementary Figs S28–S30). First, we compared Seurat, STAGATE, SEDR, conST, SpaceFlow, SpaGCN, conST, SpatialPCA, BASS, DeepST, and stDGCC onto a Slide-seqV2 dataset with 10 µm spatial resolution from the mouse hippocampus. For this comparison, we employed the annotated Allen Brain Atlas (Sunkin *et al.* 2012) as the ground truth (Fig. 3a). As shown in Supplementary Fig. S28, although Seurat, SEDR, SpaGCN, CCST, and conST were able to outline the major anatomical regions, many clusters were intermixed. STAGATE, SpatialPCA, and stDGCC produced more spatially consistent clustering and captured major anatomical regions such as CA1sp, CA3sp, and DG-sg, while SpaceFlow, BASS, and DeepST cannot recognize these biological structures. Here, stDGCC was better than STAGATE and SpatialPCA in delineating the CA3sp and DG-sg regions with sharper boundaries and higher concordance with the anatomical annotation.

Additionally, the assessment of gene marker expressions provides further substantiation for the cluster assignments derived from stDGCC (Fig. 3c and Supplementary Fig. S31). Importantly, the expression patterns of Itpka and Bcl11b elucidate distinct variations between the domains within Ammon’s horn, displaying significant upregulation specifically in the CA1sp region, which aligns consistently with existing knowledge and expectations (Lennon *et al.* 2017; Windhorst *et al.* 2017). Moreover, our analysis reveals the specific expression of Lrrtm4, a known mediator of excitatory synapse development on dentate gyrus granule cells, exclusively within the DG-sg region. This finding further supports the accurate identification and characterization of distinct biological regions facilitated by our approach (Siddiqui *et al.* 2013). In addition to well-established tissue structures, stDGCC effectively identifies numerous spatially segregated domains and unveils their respective gene expression patterns through differential expression analysis (Supplementary Fig. S31). For instance, within the hippocampal domain, excluding the “cord-like” and “arrow-like” structures (designated as domain 10), we observed robust expression of astrocyte gene markers Ddn and Camk2a, emphasizing the distinct molecular signatures associated with astrocytes within these specific regions of the hippocampus (Jouroukhin *et al.* 2018). Furthermore, domain 1, encompassing the region surrounding the hippocampus, exhibits a prominent expression of oligodendrocyte-related gene markers such as Trf and Mobp (Schäfer *et al.* 2016). Notably, stDGCC accurately demarcates the medial habenula (designated as domain 4) based on the gene marker Nwd2, and the overlap of the two regions showed a high degree of consistency (Supplementary Figs S31 and S32). These results demonstrated that stDGCC can dissect spatial heterogeneity and further uncover spatial expression patterns.

We also tested stDGCC on the mouse hippocampus dataset profiled by Slide-seq and the mouse brain dataset profiled by 10× Visium technologies. As the initial version of Slide-seqV2, the sensitivity of the transcript detection of Slide-seq is relatively lower. Compared to the 10× Visium platform with a resolution of 55 µm, Slide-seqV2 can profile spatial expressions at a resolution of cellular levels with more spots (>10 000 per section) but less sequence depth per spot. stDGCC depicted the known tissue structures well on the Slide-seq data (Fig. 3d) and 10× Visium data (Fig. 3e), respectively.

In addition, stDGCC performs very well on these three datasets with fixed parameters, as shown in Supplementary Fig. S36c–e.

### 3.8 stDGCC can identify other spatial transcriptomics data

Given the novelty of the data, we also tested stDGCC and other baseline methods on three new datasets, the Xenium mouse brain dataset, the CosMx mouse brain dataset, and the MERFISH mouse brain dataset from Vizgen. Given the absence of true labels in these datasets, we used the annotated Allen Brain Atlas (Fig. 3a) as the ground truth for comparison.

**Figure 4.**
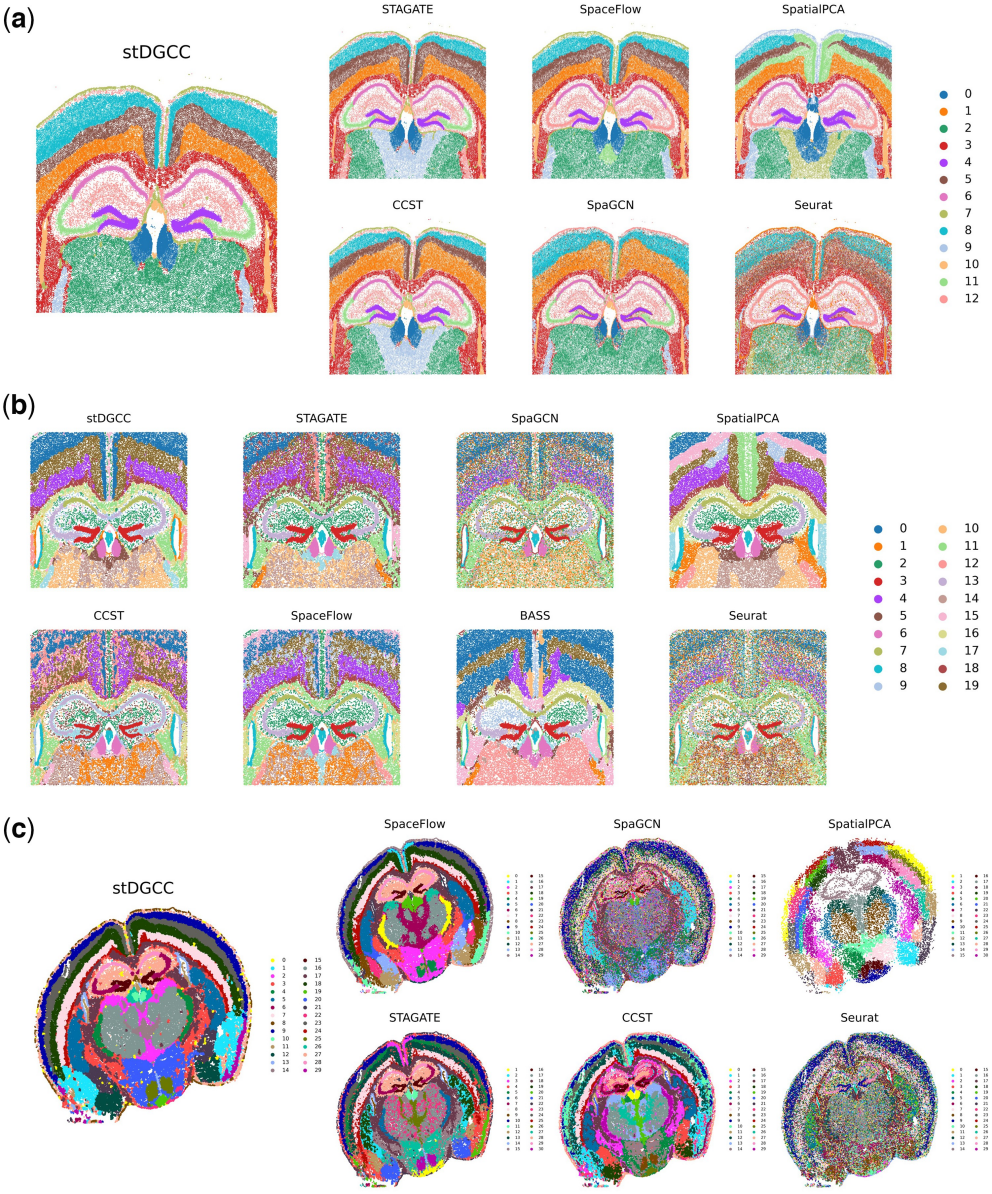
(a) Spatial domains generated by stDGCC and other baseline methods on the Xenium mouse brain dataset. (b) Spatial domains generated by stDGCC and other baseline methods on the CosMx mouse brain dataset. (c) Spatial domains generated by stDGCC and other baseline methods on the Vizgen MERFISH mouse brain dataset.

Some methods were impractical to apply for these three datasets due to high memory requirements. In the Xenium mouse brain dataset analysis, as depicted in Fig. 4a, stDGCC, STAGATE, CCST, and SpaGCN effectively identified key structural organizations within the mouse brain, such as CA1sp, CA3sp, and DG-sg. In particular, stDGCC and STAGATE provided a nuanced representation of multiple cerebral cortices, highlighting clear and symmetrical brain structures. Regarding the CosMx mouse brain dataset, Fig. 4b illustrates that most of the methodologies used successfully identified various biological structures within the mouse brain. In particular, stDGCC, STAGATE, BASS, and SpatialPCA achieved enhanced clarity and more coherent stratification of the cerebral cortex. Among them, stDGCC was distinguished by its ability to reveal detailed stratification within the cortical layers, whereas SpatialPCA facilitated the initial identification and comparison of overall brain patterns by providing a smoother structural view. The integration of these techniques enriches our understanding of the complex biological characteristics of the brain. For the Vizgen MERFISH mouse brain dataset (Fig. 4c), SpaGCN and Seurat could outline the major biological structures, but many clusters were intermixed. For the other methods, only STAGATE, CCST, and stDGCC accurately delineated the organization of key structures in the mouse brain, such as CA1sp, CA3sp, and DG-sg. Notably, stDGCC demonstrated exceptional clarity regarding biological structures among these methods. Furthermore, stDGCC also showcased a well-defined, hierarchically coherent cerebral cortex, setting it apart from the results achieved by other methods.

### 3.9 stDGCC can identify all cell cycle phases of cultured human osteosarcoma

We evaluated the performance of stDGCC in accurately detecting all cell cycle phases within the MERFISH dataset obtained from cultured human osteosarcoma. In contrast to the preprocessing methods applied to other datasets, we implemented batch correction across the three batches of this specific dataset and integrated the adjacency matrices and gene expression matrices from these three batches into a unified dataset. A *k*-means clustering approach was employed, with a selected value of *k* set at 5, aligning with the recommendations outlined in the MERFISH study (Xia *et al.* 2019). Given that cluster 5 (C5) comprised only three cells, the subsequent analysis primarily focused on the remaining four groups (C1, C2, C3, C4). To identify differentially expressed (DE) genes, we analyzed C1, C2, C3, and C4 using Scanpy and selected the top 200 DE genes for subsequent Gene Ontology (GO) term enrichment analysis.

**Figure 5.**
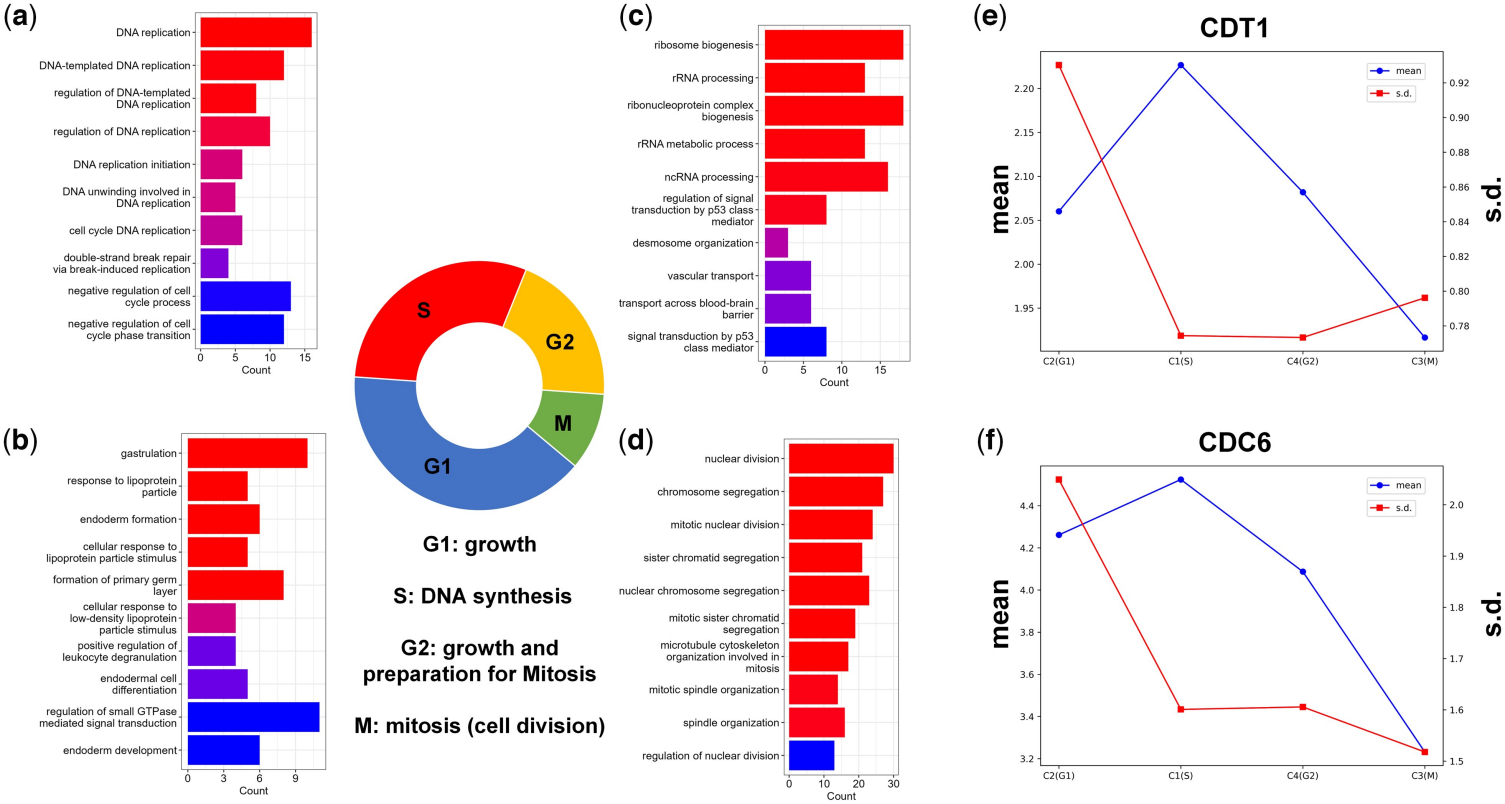
(a–d) GO terms for clustered cell groups C1 (a), C2 (b), C4 (c), and C3 (d), corresponding to S, G1, G2, and M phase, respectively. (e, f) The mean and SD of CDT1 (e) and CDC6 (f).

These findings demonstrate the effectiveness of stDGCC in accurately distinguishing those four phases of the cell cycle, as depicted in Fig. 5. The significant DE genes in C1 are predominantly associated with GO terms related to DNA replication, indicating that C1 represents cells in S-phase, which includes DNA synthesis and replication processes (Fig. 5a). On the other hand, DE genes in C4 are predominantly related to RNA- and ribosome-related GO terms (Fig. 5c), suggesting that cells in C4 are primarily in the G2 phase. During the G2 phase, cells produce macromolecules required for cellular growth and replication, in preparation for the subsequent M phase. It is crucial to highlight that cells can potentially experience arrest in the G2 phase due to factors such as DNA damage or other challenges, where p53 plays a pivotal role in impeding cell cycle progression (Taylor and Stark 2001). This observation further supports the perspective that cells in C4 are primarily situated in the G2 phase. Moreover, the prominent GO terms enriched in C3 correspond to the processes involved in the mitotic cell cycle (Fig. 5d), indicating that cells in C3 primarily reside in the M (mitosis) phase. During this phase, cells undergo the crucial process of cell division, giving rise to new progeny cells. The G1 phase of the cell cycle encompasses a multitude of intricate biological processes (Donjerkovic and Scott 2000). In the case of C2, there is a limited number of GO terms associated with regulatory functions specifically linked to the G1 phase. However, as depicted in Supplementary Fig. S3, the identification of top DE genes in C2 further supports the accuracy of stDGCC predictions. Among the most significant DE genes in C2, MALAT1, and ABI2 are notable, as both are involved in the cell cycle and play critical roles in the G1-to-S phase transition (Merlot *et al.* 2001; Tripathi *et al.* 2013; Wang *et al.* 2014). Additionally, CDT1 and CDC6 play essential roles in the initiation of DNA replication and are widely recognized as marker genes for specific cell cycle stages. Considering the mean and variance values of CDT1 and CDC6 (depicted in Fig. 5e and f), our predictions align with previous research findings (Sakaue-Sawano *et al.* 2008; Mahdessian *et al.* 2021). Specifically, CDT1 expression starts from a minimal level in G1 phase, gradually increases upon entering the S phase, and then diminishes thereafter. Moreover, variations in CDT1 expression predominantly occur in the G1 phase. Additionally, we performed some analyses on cell stage marker genes (Supplementary Fig. S33). Together, these results support the ability of stDGCC to accurately identify all four phases of the cell cycle. Correspondingly, C1, C2, C3, and C4 can be, respectively, associated with the S, G1, M, and G2 phases.

In addition, we utilized stDGCC and other baselines to evaluate the performance of different clustering methods on the MERFISH human osteosarcoma dataset. As depicted in Supplementary Figs S34 and S35, the results obtained from STAGATE reveal that C1 exhibits a strong association with the mitotic cell cycle, while C2 is closely linked to DNA replication. However, C3 and C4 do not display significant enrichment in GO terms, thus it is hard to accurately map these clusters to the cell cycle. Similarly, the findings obtained from SpaGCN indicate that C1 primarily associates with DNA replication, while C3 shows a strong correlation with the mitotic cell cycle. Although C2 demonstrates relatively fewer significant GO terms, and only ABI2 ranks third among the differentially expressed genes in C4, it becomes difficult to precisely assign these clusters to the cell cycle. In the results obtained from SpatialPCA, BASS, DeepST, conST, Seurat, and SEDR, one cluster is primarily associated with the mitotic cell cycle, while the other two clusters are closely related to DNA replication. However, the remaining cluster shows low significance in terms of enriched GO terms, and neither MALAT1 nor ABI2 is highly ranked among the differentially expressed genes in any of the four clusters, making it impossible to map the four clusters to the four cell cycle stages. Conversely, in the results obtained from CCST, stDGCC, and SpaceFlow, three of the clusters are, respectively, associated with the mitotic cell cycle, DNA replication, and RNA biogenesis. Finally, we elucidate the impact of spatial information on the clustering results of this dataset in Supplementary Figs S2–S4.

## 4 Discussion

In this article, we propose a deep graph contrastive clustering framework, stDGCC, which uses a spatially informed graph node embedding model to extract both spatial and cellular information from ST data in an unsupervised manner. Furthermore, we construct both positive and negative graphs for contrastive learning, thus enabling generic semantic representation learning across different modalities. Experimental results on the DLPFC dataset demonstrate that the proposed stDGCC method outperforms other state-of-the-art methods. In addition to recognizing small differences in similar spots, stDGCC can identify tissue structures from ST data of different spatial resolutions. Lastly, we performed parametric and ablation analyses that demonstrate the robustness of the proposed stDGCC methods from multiple perspectives and further explored the intrinsic components of the model’s effective mechanism. With the rapid advancement of ST technology, it is possible to measure a large number of cells at high spatial resolutions. However, the substantial memory required to load the entire graph and limited computational resources restricts their application on very large datasets. Therefore, optimizing memory efficiency through batching and parallel processing techniques will be an important area for future work.

## Supplementary Material

btae451_Supplementary_Data
