# Supplementary material for: Unraveling spatial domain characterization in spatially resolved transcriptomics with robust graph contrastive clustering
Source: Bioinformatics. 2024 Jul 16;40(7):btae451. doi: 10.1093/bioinformatics/btae451 (PMC11272174; doi:10.1093/bioinformatics/btae451)
Supplement: btae451_Supplementary_Data [file btae451_supplementary_data.pdf]

Supplementary Material to ”Unraveling Spatial Domain Characterization in Spatially Resolved Transcriptomics with Robust Graph Contrastive Clustering”

Yingxi Zhang [1] , Zhuohan Yu [1] , Ka-chun Wong [2] and Xiangtao Li [1*]

Contents

1 Supplementary Note 1: Summary of datasets **2**

2 Supplementary Note 2: Running time of stDGCC under different datasets **2**

3 Supplementary Note 3: Additional analyses of the MERFISH human osteosarcoma dataset **3**

4 Results for Section 3.4 **4**

5 Results and Supplementary for Section 3.5 **5**

6 Results for Section 3.6 **8**

7 Results and Supplementary for Section 3.7 **8**

8 Results and Supplementary for Section 3.9 **9**

9 Supplementary Note 4: Results of stDGCC on different datasets with fixed parameters **9**

1 Supplementary Note 1: Summary of datasets

Table 1: Description of all ST datasets used in this study.

Platform	Tissue	Section	Spots	Related Figures
10x Visium	DLPFC	151507	4226	Fig. 2, Figs. S5-S7, Figs. S25-S27
		151508	4384	
		151509	4789	
		151510	4634	
		151669	3661	
		151670	3498	
		151671	4110	
		151672	4015	
		151673	3639	
		151674	3673	
		151675	3592	
		151676	3460	
	Mouse Brain	Mouse Brain Section (Coronal)	2702	Fig. 3e, Figs. S18-S22, Fig. S30
Slide-seqV2	Mouse Hippocampus	Puck_190921_21	20143	Fig. 3b, Figs. S13-S17, Fig. S28, Figs. S31-S32
Slide-seq	Mouse Hippocampus	Puck_180531_23	18508	Fig. 3d, Figs. S8-S12, Fig. S29
MERFISH	Human Osteosarcoma	pnas.1912459116.sd12	1368	Fig. 5, Figs. S2-S4, Figs. S23-S24, Figs. S33-S35
	Mouse Brain	BrainReceptorShowcase_Slice2_Replicate1	83546	Fig. 4c
CosMx	Mouse Brain	Run5642_S3_Quarter	38996	Fig. 4b
Xenium	Mouse Brain	Xenium_V1_FF_Mouse_Brain_MultiSection_1	162033	Fig. 4a

2 Supplementary Note 2: Running time of stDGCC under different datasets

In our study, the computational time of the model is a key factor in assessing its feasibility for real-world applications. Our stDGCC model was trained and tested on an Ubuntu server with standard specifications (detailed hardware configuration: an NVIDIA GTX 2080Ti GPU with 24 GB of memory). To evaluate the runtime performance of stDGCC, we conducted runtime measurements on multiple datasets, namely the DLPFC dataset, 10x Visium mouse brain dataset, Slide-seqV2 mouse hippocampus dataset, Slide-seq mouse hippocampus dataset, and MERFISH human osteosarcoma dataset. These runtimes are the sum of the runtimes of the various stages, including model training, PCA processing, and K-means clustering.

Figure S1 shows the runtime of the stDGCC model across various datasets. These include the 10x Visium mouse brain dataset (denoted as ”MB” in the figure, which, after undergoing a cutting process, contains 691 points), the MERFISH human osteosarcoma dataset (represented as ”U-2 OS,” comprising 1,368 spots), the Slide-seq mouse hippocampus dataset (indicated by ”MH,” encompassing 18,508 spots), and the Slide-seqV2 mouse hippocampus dataset (represented as ”MH2,” containing 20,143 spots). Additionally, we analyzed the DLPFC dataset, which consists of 12 sections, with spot counts ranging between 3,460 and 4,798.

As demonstrated, for the 10x Visium mouse brain dataset, the runtime was only 76.7 seconds, and the runtime of stDGCC varies between 157.8 seconds and 211.9 seconds for the 12 sections of the DLPFC dataset. For the Slide-seq mouse hippocampus dataset and the Slide-seqV2 mouse hippocampus dataset, the runtime of stDGCC is 826.7 seconds and 994.9 seconds, respectively. Overall, the runtime of stDGCC is primarily influenced by the number of spots present in the dataset.

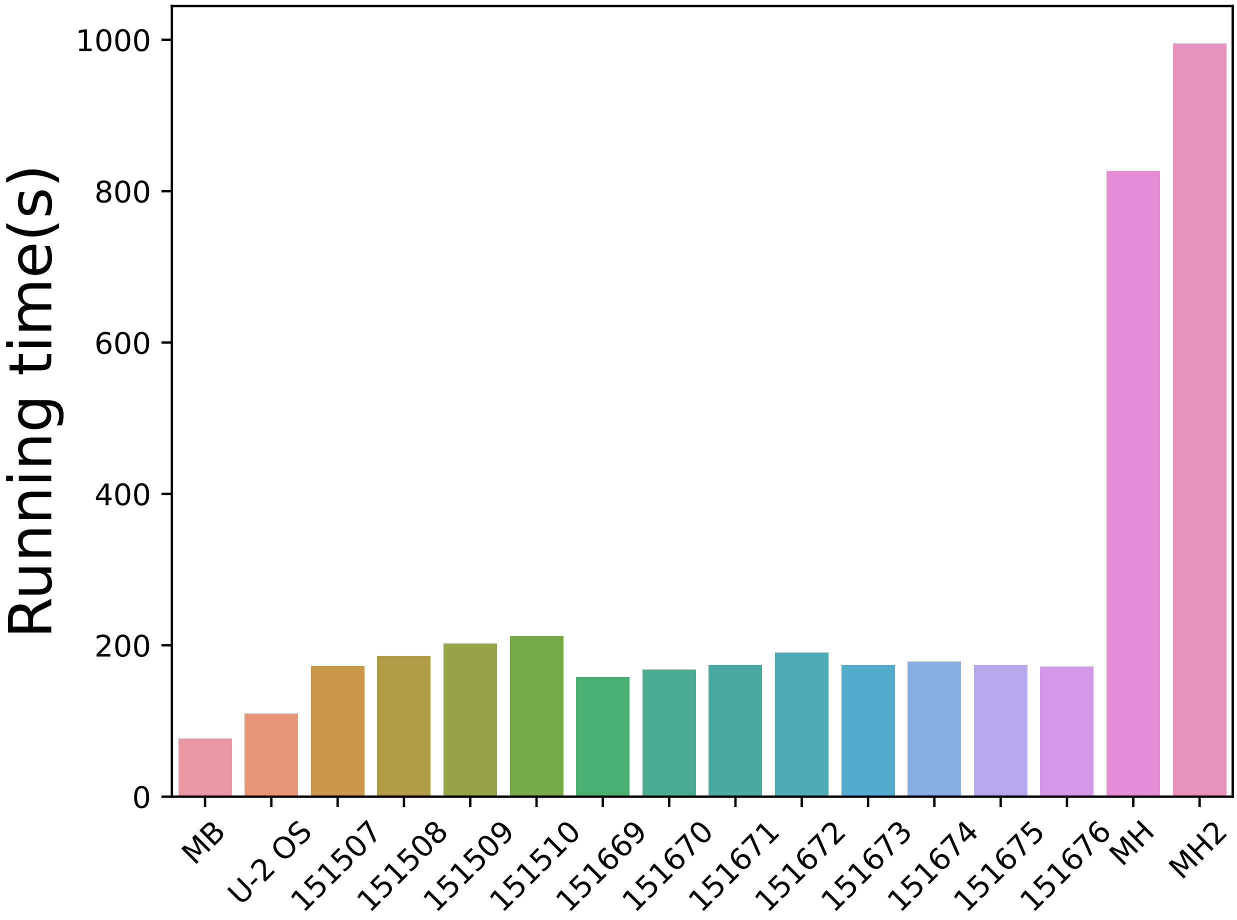

Figure S1: Running time of stDGCC on different datasets

3 Supplementary Note 3: Additional analyses of the MERFISH human osteosarcoma dataset

To elucidate the impact of spatial information on the clustering of human cell lines, we conducted a comparative analysis using the stDGCC algorithm on the MERFISH human osteosarcoma dataset, both with and without incorporating spatial information. The analysis proceeded as follows: we first applied stDGCC to generate clustering results under both conditions. These results were then subjected to a detailed examination using Scanpy, focusing specifically on the top 200 differentially expressed genes to conduct a Gene Ontology (GO) term enrichment analysis. This approach yielded the top-10 GO terms for each cluster. For comparative clarity, we aligned key GO terms with distinct cell cycle stages, utilizing keywords such as S for DNA replication, G2 for RNA and biogenesis, and M for mitotic processes and related terms (e.g., mitosis, spindle, division, segregation, and fission). Given the G1 phase's biological intricacy, marker genes MALAT1 and ABI2 were integrated to assist in delineating the G1 phase (Tripathi *et al.* 2013; Wang *et al.* 2014; Merlot *et al.* 2001).

The comparative results, presented in Figures S2 and S3, illustrate a marked distinction between the use of spatial information and its absence. Without spatial data, the results reveal that cluster C3 exhibits a strong association with the mitotic cell cycle, while cluster C4 is closely linked to DNA replication and RNA biogenesis. In addition, the marker gene MALAT1 was ranked high in both C2 and C3, making it a challenge when attempting to map clusters to distinct cell cycle stages without

spatial information. In contrast, with the incorporation of spatial information, we observed a more distinct association of clusters with specific cell cycle stages. Clusters C1, C3, and C4 could be confidently associated with the DNA replication, mitotic cell cycle, and RNA biogenesis, respectively. Importantly, the integration of spatial data allowed cluster C2 to be distinctly identified through the high ranking of both MALAT1 and ABI2 among its differentially expressed genes, facilitating the accurate mapping of these four clusters to their corresponding cell cycle stages.

To further explore the spatial clustering of cells at different stages, we analyzed the neighbor enrichment ratios for clusters C1 through C4, as depicted in Figure S4. In this analysis, we initially identified all neighboring cells associated with each group using the original adjacency matrix. We then quantified the number of neighbors belonging to each specific group and calculated the ratio, thereby deriving the neighbor enrichment ratio. The obtained ratios unequivocally demonstrate that cells tend to spatially cluster with other cells from the same group, corroborating findings previously reported in the literature (Xia *et al.* 2019).

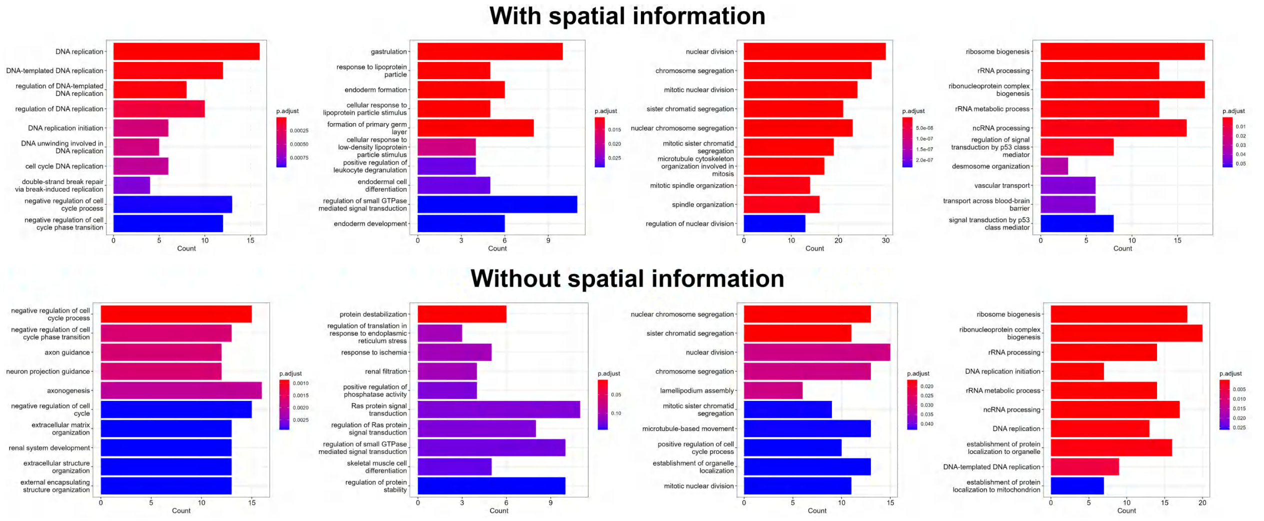

Figure S2: GO term enrichment analysis of clustering results from stDGCC with and without spatial information on the MERFISH human osteosarcoma dataset. The GO terms belong to C1, C2, C3, and C4 from left to right.

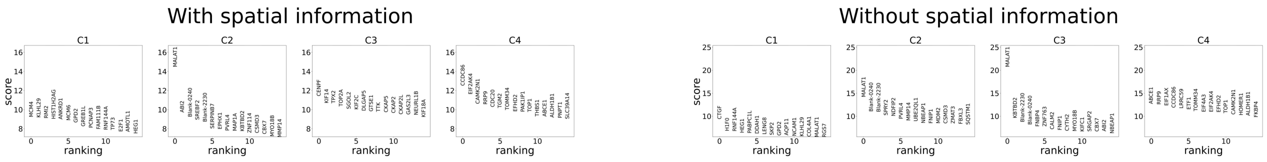

Figure S3: Top 15 DE genes of C1, C2, C3, and C4 of stDGCC with and without spatial information on the MERFISH human osteosarcoma dataset.

4 Results for Section 3.4

Table 2, Figure S5, and Figure S6.

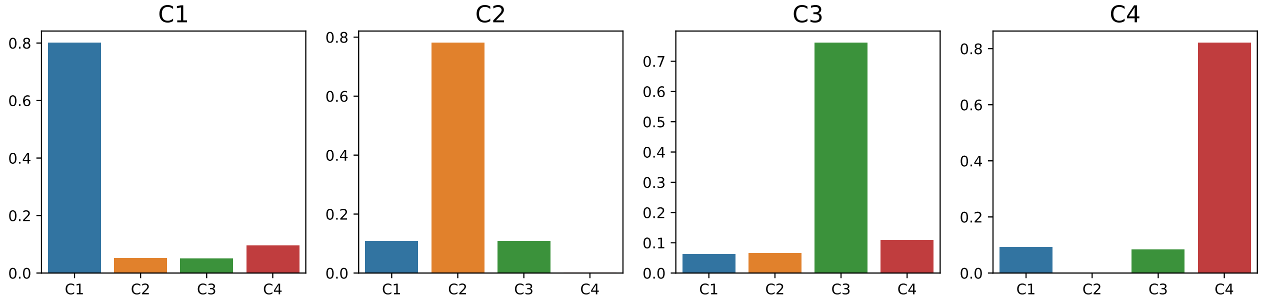

Figure S4: The neighbor enrichment ratios of C1, C2, C3, and C4 of stDGCC with spatial information on the MERFISH human osteosarcoma dataset.

Table 2: Performance of our method and other baseline methods on 12 sections of the DLPFC dataset. Bold font indicates the highest values of all the compared methods.

	Datasets	ours	Deep Embedded Clustering Methods					Deep Contrastive Learning Methods			Base Clustering		
		stDGCC	SEDR	SpaGCN	STAGATE	stLearn	DeepST	CCST	conST	SpaceFlow	SpatialPCA	BASS	Seurat
ARI	151507	**0.598**	0.451	0.449	0.526	0.493	0.519	0.493	0.408	0.419	0.503	0.547	0.339
	151508	0.508	0.377	0.362	**0.511**	0.315	0.429	0.408	0.24	0.295	0.475	0.499	0.4
	151509	**0.62**	0.363	0.373	0.359	0.414	0.516	0.432	0.292	0.223	0.466	0.406	0.223
	151510	**0.574**	0.344	0.424	0.451	0.444	0.486	0.385	0.312	0.282	0.429	0.424	0.276
	151669	**0.527**	0.368	0.225	0.452	0.326	0.415	0.344	0.434	0.31	0.446	0.409	0.303
	151670	**0.506**	0.346	0.374	0.431	0.228	0.332	0.33	0.363	0.227	0.389	0.373	0.334
	151671	0.638	0.41	0.501	0.579	0.389	0.405	**0.667**	0.496	0.361	0.614	0.543	0.273
	151672	0.603	0.475	0.564	0.589	0.347	0.547	**0.632**	0.446	0.401	0.592	0.437	0.191
	151673	0.565	0.464	0.461	0.49	0.305	**0.591**	0.39	0.426	0.347	0.537	0.588	0.388
	151674	**0.593**	0.508	0.432	0.414	0.386	0.516	0.539	0.367	0.332	0.53	0.497	0.304
	151675	0.436	0.55	0.3	0.592	0.384	**0.605**	0.545	0.45	0.406	0.447	0.539	0.293
	151676	0.474	0.412	0.329	0.516	0.4	0.484	0.611	0.496	0.373	**0.617**	0.54	0.311
NMI	151507	**0.693**	0.555	0.559	0.679	0.646	0.62	0.654	0.512	0.533	0.62	0.68	0.408
	151508	0.633	0.448	0.478	**0.65**	0.529	0.57	0.594	0.375	0.449	0.61	**0.65**	0.426
	151509	**0.729**	0.573	0.541	0.583	0.608	0.64	0.626	0.485	0.415	0.63	0.61	0.361
	151510	**0.689**	0.547	0.546	0.619	0.598	0.62	0.597	0.43	0.47	0.577	0.61	0.401
	151669	0.603	0.534	0.373	0.6	0.501	0.58	0.547	**0.616**	0.487	0.61	0.6	0.297
	151670	0.559	0.502	0.486	0.558	0.41	0.51	0.476	0.546	0.401	**0.56**	**0.56**	0.322
	151671	**0.722**	0.591	0.611	0.675	0.549	0.6	0.654	0.585	0.469	0.71	0.67	0.31
	151672	**0.701**	0.595	0.658	0.692	0.491	0.66	0.657	0.565	0.521	0.69	0.61	0.309
	151673	0.702	0.625	0.625	0.672	0.497	**0.71**	0.596	0.599	0.497	0.69	**0.71**	0.414
	151674	**0.724**	0.615	0.535	0.564	0.551	0.62	0.648	0.524	0.437	0.65	0.62	0.39
	151675	0.587	0.64	0.458	0.688	0.563	**0.72**	0.666	0.569	0.519	0.63	0.69	0.381
	151676	0.614	0.594	0.515	0.628	0.571	0.65	0.686	0.653	0.492	**0.72**	0.66	0.413

5 Results and Supplementary for Section 3.5

In our study, hyperparameter adjustments were not confined to a single dataset; instead, they were applied across twelve sections of the human dorsolateral prefrontal cortex (DLPFC). After that, considering the differences in biological conditions, experimental techniques, and the absence of real labels in these datasets, we did not directly apply the best parameters obtained from the DLPFC dataset to several other datasets. After determining an effective parameter range, we further independently optimized hyperparameters on the 10x Visium mouse brain dataset, the Slide-seqV2 mouse hippocampus dataset, and the Slide-seq mouse hippocampus dataset, ensuring that our algorithm could adapt to the distinct characteristics of multiple datasets.

Our study meticulously explored the influence of three critical hyperparameters: λ, δ, and the number of highly variable genes, on our model's performance. The parameter λ determined the weighting of individual nodes relative to their neighbors in the convolution process. The δ parameter was crucial for establishing the density of edges within the spatial graph, indicating the magnitude of neighboring nodes. Furthermore, the number of highly variable genes directly influenced the amount of effective

information and noise present within the ST data.

Before performing hyperparameter optimization, we synthesized the hyperparameter settings from the existing literature on previous studies (Li *et al.* 2022; Dong and Zhang 2022) to establish our parameter range. Subsequently, we performed a detailed hyperparameter search on the DLPFC dataset, with the aim of exploring the parameter space and identifying ranges that yield good performance. Then, we did not directly apply the best parameters obtained from the DLPFC datasets to several other datasets, due to the different biological conditions, experimental techniques, and the lack of real labels in those datasets. Instead, we adopted a more comprehensive and systematic approach. Upon establishing an efficient parameter range, we proceeded to conduct individual hyperparameter optimization on the 10x Visium mouse brain dataset, the Slide-seqV2 mouse hippocampus dataset, and the Slide-seq mouse hippocampus dataset. This meticulous approach aimed to guarantee the adaptability of our algorithm to the unique characteristics exhibited by each dataset.

For each dataset, λ, δ, and the number of highly variable genes were varied within specified ranges to assess their impact systematically. Specifically, λ was tested across [0.1, 0.2, 0.3, 0.4, 0.5, 0.6, 0.7, 0.8, 0.9], δ across [50, 100, 150, 250, 350, 400], and the number of highly variable genes across [2000, 3000, 4000, 5000, 6000]. To ensure a comprehensive evaluation of the performance, we exhaustively enumerated these hyperparameter combinations, resulting in a total of 270 distinct hyperparameter configurations, enabling a detailed comparison of clustering performances.

Figure S7 elucidates the stDGCC model's efficacy on the DLPFC datasets under these varied settings, revealing discernible trends correlating parameter values with model performance. Significant performance degradation was noted when δ values were at the lower thresholds of 50 and 100, primarily due to the insufficient number of neighboring nodes, which compromises the model's structural capture capability. Moreover, elevating the count of highly variable genes above 4000 detrimentally introduced noise, thus deteriorating model performance and emphasizing the critical importance of optimal gene selection. While variations in λ did influence outcomes, their effect was relatively marginal in comparison. Based on these observations from Figure S7, we can find that $\lambda = 0.8$, $\delta = 250$, and 3000 highly variable genes can provide the best performance in our main analysis for the DLPFC datasets.

After that, we applied the same parameter setting intervals to analyze the 10x Visium mouse brain dataset, the Slide-seqV2 mouse hippocampus dataset, and the Slide-seq mouse hippocampus dataset. In light of the lack of genuine labels in these datasets, comparison and evaluation were facilitated by adopting a methodology akin to that employed in preceding studies (Dong and Zhang 2022; Xu *et al.* 2022; Shang and Zhou 2022). Clustering outcomes were initially visualized using spatial information, followed by manual assessment aligned with the Allen mouse brain reference atlas (Sunkin *et al.* 2012) (**Figure 3a**), focusing on the clarity of clustering results and the delineation of known tissue structures such as CA1sp, CA3sp, and DG-sg. By employing this methodology, we discerned distinct patterns and trends linked to different parameter configurations.

Figures S18, S19, S20, S21, and S22 elucidate the stDGCC model's efficacy on the 10x Visium mouse brain dataset under diverse parameter settings. Remarkably, when δ is set to 50 and 100, the stDGCC model successfully identifies distinct organizational structures (CA1sp, CA3sp, and DG-sg) that exhibit high consistency with the morphological picture. In contrast, higher δ values led to more 'inflated' representations of biological structures; for instance, CA3sp transitioned from a slender to a more aggregated form. Similar to the findings with the DLPFC dataset, we found that λ had a minimal impact on the clustering results for the 10x Visium mouse brain dataset. Additionally, an

increase in the number of highly variable genes was observed to inflate the proportion of noise within identified biostructures, attributable to the model's heightened sensitivity to the noise accompanying a greater count of highly variable genes. Based on these observations in Figures S18, S19, S20, S21, and S22, the clustering outcomes for $\lambda = 0.8$, $\delta = 100$, and 3000 highly variable genes were selected for inclusion in the main text.

The analysis of the Slide-seqV2 mouse hippocampus dataset, as illustrated in Figures S13, S14, S15, S16, and S17 demonstrated that the number of highly variable genes and λ have minimal impact on the clustering results. These parameters appeared to have negligible effects on both the accuracy and the distinctiveness of the biological structures identified within the dataset. Notably, with δ values ranging from 50 to 150, the clustering algorithm was able to accurately identify known tissue structures in the mouse hippocampus, such as CA1sp, CA3sp, and DG-sg, among others. However, increasing the δ parameter beyond the range of 50 to 150 led to a notable aggregation of data points into larger clusters. This aggregation complicates the precise identification and differentiation of specific biological structures within the dataset. Based on these observations in Figures S13, S14, S15, S16, and S17, we have selected clustering outcomes for $\lambda = 0.8$, $\delta = 100$, and 3000 highly variable genes for detailed presentation in the main text. This parameter configuration optimizes the balance between structural clarity and the delineation of biological structures within the dataset.

Figures S8, S9, S10, S11, and S12 exhibited that the parameter λ has a minimal impact on the mouse hippocampus dataset derived from Slide-seq. Limiting the analysis to 2000 highly variable genes often resulted in the conflation of CA1sp and CA3sp within the same cluster, suggesting that this gene set lacks sufficient discriminative information. Nevertheless, the identifiable tissue structures of the mouse hippocampus, including CA1sp, CA3sp, and DG-sg, were accurately recognized within the δ range of 50 to 150 when employing 3000 highly variable genes. However, increasing the number of highly variable genes to beyond 4000 introduced difficulties in distinguishing CA3sp at a δ setting of 50, likely due to the diminished number of neighboring nodes and augmented noise levels. Based on these insights from Figures S8, S9, S10, S11, and S12, the clustering parameters of $\lambda = 0.8$, $\delta = 50$, and 3000 highly variable genes were selected for detailed discussion in the main text.

In consideration of the higher resolution of the MERFISH human osteosarcoma dataset, we selected a range of δ values from 50 to 400 (specifically, [50, 100, 150, 200, 250, 300, 350, 400]), while keeping the range of λ values unchanged. To evaluate their performance, GO enrichment analysis was conducted. After obtaining the top 10 GO terms, For comparative clarity, we aligned key GO terms with distinct cell cycle stages, utilizing keywords such as S for DNA replication, G2 for RNA and biogenesis, and M for mitotic processes and related terms (e.g., mitosis, spindle, division, segregation, and fission). Given the G1 phase's biological intricacy, marker genes MALAT1 and ABI2 were integrated to assist in delineating the G1 phase (Tripathi *et al.* 2013; Wang *et al.* 2014; Merlot *et al.* 2001). The results, as depicted in Figures S23 and S24, indicate that λ has a relatively minor influence on the model when δ is below 150. At this point, the clustering results obtained from stDGCC revealed one cluster associated with the M phase and another cluster associated with DNA replication. However, it was not feasible to map these clusters to specific stages of the cell cycle. As δ gradually increased, the stDGCC results exhibited three clusters linked to RNA, DNA replication, and mitosis, respectively. In the remaining cluster, the marker genes MALAT1 and ABI2 ranked high among the differentially expressed genes, enabling the mapping of four clusters to four cell cycles. Notably, when δ exceeded 350, the clustering outcomes of stDGCC included one cluster associated with the M phase, one cluster associated with DNA replication, one cluster where the marker genes MALAT1 and ABI2 ranked high among the differentially expressed genes, and an additional cluster that could not be matched to the G2 phase. Based on these insights, We use λ and δ in the main text as 0.8 and 200, respectively.

In addition to analyses of hyperparameters, we experimentally explored the impact of varying the number of principal components and the number of layers and nodes in our proposed stDGCC model. We delineated our model variants into four configurations: stDGCC1, stDGCC2, stDGCC3, and stDGCC4 (our proposed stDGCC), correlating to the encoder layers, specifically layer one, layer two, layer three, and layer four, with corresponding neuron node configurations of [128], [256-128], [512-256-128], and [1028-512-256-128], respectively. The decoder architecture was designed to be symmetrical to the encoder. In exploring the principal component analysis, our assessments spanned 10, 20, 30, 60, and 90 components, with an additional scenario excluding PCA (utilizing 128 components directly). Our systematic evaluation encompassed 24 distinct model configurations, benchmarking their clustering effectiveness on the DLPFC dataset. As shown in Figure S25, the number of principal components between 20 and 128 has a relatively minor impact on the model's performance. However, a reduction in the number of layers and nodes is associated with a notable decrease in the model's overall effectiveness. Specifically, the stDGCC model, when configured to employ the top 30 principal components, attains the highest average metrics on the DLPFC dataset, with an Adjusted Rand Index (ARI) of 0.554 and a Normalized Mutual Information (NMI) score of 0.663. This configuration not only ensures superior clustering results but also contributes to a reduction in computational time required for the subsequent analysis.

Finally, we also analyzed the weights of the objective functions of the different modules. In this paper, the weight coefficients were chosen (after some preliminary experiments) to give robust results. We collected and summarized different weight coefficients from previous studies (Li *et al.* 2022; Ren *et al.* 2022). On this basis, α, β, and γ were selected from [1.0, 2.0, 3.0, 4.0], [0.02, 0.05, 0.1], and [0.005, 0.015, 0.025] respectively. Then, to provide comprehensive performance evaluations, we enumerated them to obtain 36 distinct loss weight assignments and compared the clustering performance on the DLPFC dataset in those scenarios (Figure S26). From Figure S26, we can observe that variation in weight loss over a certain range has little effect on the clustering performance of stDGCC in most data sets. For the weights of Lc, α changes in it also cause fluctuations in the model performance. For instance, with β and γ held constant at 0.05 and 0.005, respectively, increasing α from 1.0 to 2.0 enhances the average Adjusted Rand Index (ARI) from 0.517 to 0.554. Further increasing α to 4.0 results in a slight decrease in average ARI to 0.549. Optimal clustering performance, measured by both the average NMI and ARI, was observed when α, β, and γ were set to [2.0, 0.05, 0.005] out of the 36 tested configurations.

6 Results for Section 3.6

Figure S27

7 Results and Supplementary for Section 3.7

Regarding the inclusion of quantitative results, the inherent characteristics of the first dataset, DLPFC, which comprises 12 human dorsolateral prefrontal cortex sections, allow for the use of NMI and ARI as quantitative metrics for evaluating the performance of different algorithms. These sections have been manually annotated, with the DLPFC layer and white matter (WM) acting as the ground truth (Maynard *et al.* 2020). In contrast, the remaining datasets, including the Slide-seqv2 mouse hippocampus dataset, the Slide-seq mouse hippocampus dataset, and the 10x Visium mouse brain dataset, lack actual labels. Consequently, they cannot be directly assessed using common evaluation metrics such as ARI or NMI. This predicament is not isolated to our study; previous

research, including studies like STAGATE (Dong and Zhang 2022) and SpatialPCA (Shang and Zhou 2022), also refrained from using quantitative evaluation methods on these unlabeled datasets. This highlights the inherent challenge in applying quantitative metrics to directly evaluate a model's performance in the absence of real labels.

To quantitatively evaluate these datasets, we conducted exploratory computations on these unlabeled datasets using the Average Silhouette Width (ASW) (Rousseeuw 1987). ASW serves as an internal evaluation metric for cluster analysis and is particularly suitable for assessing the rationality of clustering in the absence of external label validation (Batool and Hennig 2021; Yu *et al.* 2023). It evaluates the clustering quality by considering both the cohesion within clusters and the separation between them, calculated using the following formula:

$$ASW\left(x\right) = \frac{1}{n}\sum_{i=1}^{n}\left(\frac{b\left(i\right) - a\left(i\right)}{max\left\{a\left(i\right), b\left(i\right)\right\}}\right) \tag{1}$$

where $a\left(i\right)$ denotes the average distance from x_i to all the other data points in the cluster to which x_i belongs, and $b\left(i\right)$ denotes the minimum average distance from x_i to all other clusters to which o does not belong. The value of ASW is between $[-1, 1]$. If ASW is close to 1, it means that the clustering of the data object x is reasonable; if ASW is close to -1, it means that the division of x is inaccurate; if ASW is approximately 0, it implies that many data points in x are on the boundary of the two clusters. The ASW measures the reasonableness and validity of the clustering results. The results of stDGCC and other baselines on the Slide-seqv2 mouse hippocampus dataset, the Slide-seq mouse hippocampus dataset, and the 10x Visium mouse brain dataset are presented in Figures S28, S29, and S30.

8 Results and Supplementary for Section 3.9

Through a comprehensive literature review and employing differential gene analysis techniques, we have identified a set of genes that demonstrate associations with the cell cycle. To visually represent these findings, we have utilized violin plots (Figure S33). Notably, MCM5 has been established as a recognized marker for the G1 phase (Whitfield *et al.* 2002). Similarly, CDC20 has been acknowledged as a marker specifically for the G2/M phase (Weinstein 1997; Weinstein *et al.* 1994). The gene MCM6, which plays a crucial role in S phase progression and is anticipated to be up-regulated in G1/S cells while down-regulated during G2/M transition (Kearsey *et al.* 1996), exhibited up-regulation in the C1 and C2 clusters while displaying down-regulation in the C3 and C4 clusters. The expression level of the KIF2C gene was found to be remarkably high in the C3 cluster, aligning with prior knowledge that KIF2C is vital for the M phase of the cell cycle (Maney *et al.* 1998).

9 Supplementary Note 4: Results of stDGCC on different datasets with fixed parameters

Considering the different biological conditions and experimental techniques, which may lead to significant differences between datasets, we initially did not use the same parameters across all eight datasets. We added a set of experiments with fixed stDGCC parameters to facilitate fair comparisons with other methods. In particular, the δ parameter is significantly influenced by sequencing technologies, making it impractical to fix this parameter. This situation is similar to other benchmark methods like STAGATE, which also adjusts the δ parameter for different sequencing technologies in its tutorials.

In this experiment, we fixed five parameters: λ, the number of highly variable genes, α, β, and γ, set to [0.8, 3000, 2.0, 0.2, 0.005]. For datasets with fewer than 3000 genes, we did not select highly variable genes. This parameter setting helps us to test and validate the performance across different datasets, ensuring consistency and comparability of the results.

For the DLPFC dataset, we computed the ARI and NMI for stDGCC and other baseline methods and visualized them as box plots (Figure S36a). As shown in Figure S36a, the median ARI and NMI of stDGCC were significantly higher than those of the other baseline methods. In addition, although the average ARI of stDGCC (0.526) has decreased from the previous evaluation, it still maintains an advantage over other models.

For the 10x Visium mouse brain dataset, the results of stDGCC and other baseline methods are shown in Figure S36c. Notably, stDGCC, STAGATE, SpatialPCA, DeepST, SpaGCN, and Seurat are particularly effective in identifying established structural organizations within the mouse brain, such as the CA1sp, CA3sp, and DG-sg. For the Slide-seq mouse hippocampus dataset, the results of stDGCC and other baselines are visualized in Figure S36d. As depicted in this figure, stDGCC and STAGATE exhibited remarkable proficiency in identifying established structural organizations within the mouse brain, including CA1sp, CA3sp, and DG-sg. Conversely, alternative methods struggled to distinguish between DG-sg and the hippocampal horn cone cell layers. For the Slide-seqV2 mouse hippocampus dataset, the results of stDGCC and other baselines are visualized in Figure S36e. Although Seurat, SEDR, SpaGCN, CCST, and conST were able to outline major anatomical regions, many clusters were intermixed. STAGATE, SpatialPCA, and stDGCC produced more spatially consistent clustering and captured major anatomical regions such as CA1sp, CA3sp, and DG-sg, while SpaceFlow, BASS, and DeepST can not recognize these biological structures.

For the other datasets, the parameters are consistent with the main text, and the specific results and analyses are presented in the main text. In this comprehensive evaluation, stDGCC has consistently demonstrated commendable performance across diverse datasets by employing fixed parameters.

References

Batool, F. and Hennig, C. (2021). Clustering with the average silhouette width. *Computational Statistics & Data Analysis*, **158**, 107190.

Dong, K. and Zhang, S. (2022). Deciphering spatial domains from spatially resolved transcriptomics with an adaptive graph attention auto-encoder. *Nature communications*, **13**(1), 1–12.

Kearsey, S. E. *et al.* (1996). The role of mcm proteins in the cell cycle control of genome duplication. *Bioessays*, **18**(3), 183–190.

Li, J. *et al.* (2022). Cell clustering for spatial transcriptomics data with graph neural networks. *Nature Computational Science*, **2**(6), 399–408.

Maney, T. *et al.* (1998). Mitotic centromere–associated kinesin is important for anaphase chromosome segregation. *The Journal of cell biology*, **142**(3), 787–801.

Maynard, K. E. *et al.* (2020). Transcriptome-scale spatial gene expression in the human dorsolateral prefrontal cortex. *Cold Spring Harbor Laboratory*, (3).

Merlot, S. *et al.* (2001). The abi1 and abi2 protein phosphatases 2c act in a negative feedback regulatory loop of the abscisic acid signalling pathway. *The Plant Journal*, **25**(3), 295–303.

Ren, H. *et al.* (2022). Identifying multicellular spatiotemporal organization of cells with spaceflow. *Nature communications*, **13**(1), 4076.

Rousseeuw, P. J. (1987). Silhouettes: a graphical aid to the interpretation and validation of cluster analysis. *Journal of computational and applied mathematics*, **20**, 53–65.

Shang, L. and Zhou, X. (2022). Spatially aware dimension reduction for spatial transcriptomics. *Nature Communications*, **13**(1), 7203.

Sunkin, S. M. *et al.* (2012). Allen brain atlas: an integrated spatio-temporal portal for exploring the central nervous system. *Nucleic acids research*, **41**(D1), D996–D1008.

Tripathi, V. *et al.* (2013). Long noncoding rna malat1 controls cell cycle progression by regulating the expression of oncogenic transcription factor b-myb. *PLoS genetics*, **9**(3), e1003368.

Wang, J. *et al.* (2014). Malat1 promotes cell proliferation in gastric cancer by recruiting sf2/asf. *Biomedicine & Pharmacotherapy*, **68**(5), 557–564.

Weinstein, J. (1997). Cell cycle-regulated expression, phosphorylation, and degradation of p55cdc: a mammalian homolog of cdc20/fizzy/slp1. *Journal of Biological Chemistry*, **272**(45), 28501–28511.

Weinstein, J. *et al.* (1994). A novel mammalian protein, p55cdc, present in dividing cells is associated with protein kinase activity and has homology to the saccharomyces cerevisiae cell division cycle proteins cdc20 and cdc4. *Molecular and cellular biology*, **14**(5), 3350–3363.

Whitfield, M. L. *et al.* (2002). Identification of genes periodically expressed in the human cell cycle and their expression in tumors. *Molecular biology of the cell*, **13**(6), 1977–2000.

Xia, C. *et al.* (2019). Spatial transcriptome profiling by merfish reveals subcellular rna compartmentalization and cell cycle-dependent gene expression. *Proceedings of the National Academy of Sciences*, **116**(39), 19490–19499.

Xu, C. *et al.* (2022). Deepst: identifying spatial domains in spatial transcriptomics by deep learning. *Nucleic Acids Research*, **50**(22), e131–e131.

Yu, Z. *et al.* (2023). Topological identification and interpretation for single-cell gene regulation elucidation across multiple platforms using scmgca. *Nature Communications*, **14**(1), 400.

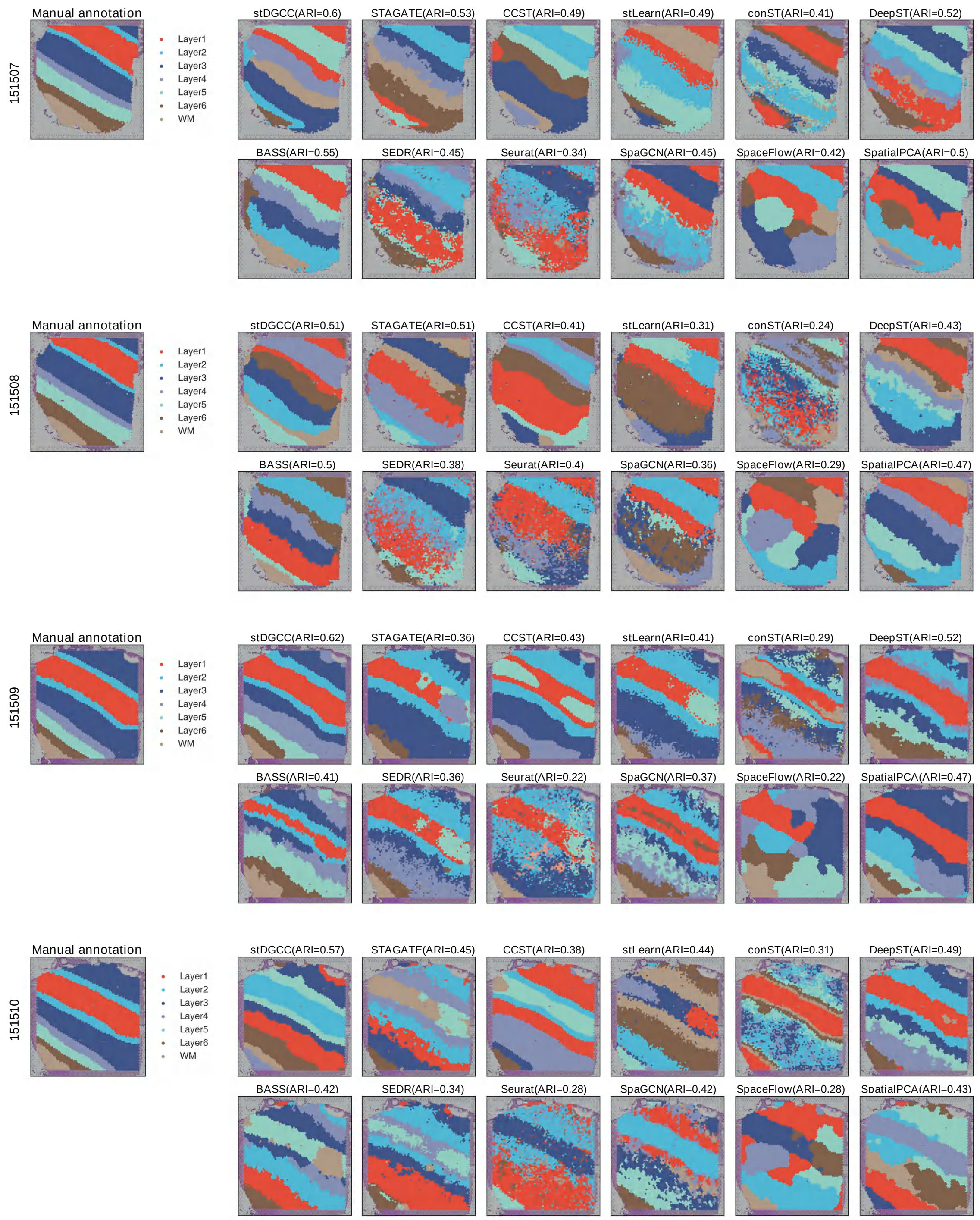

See next page

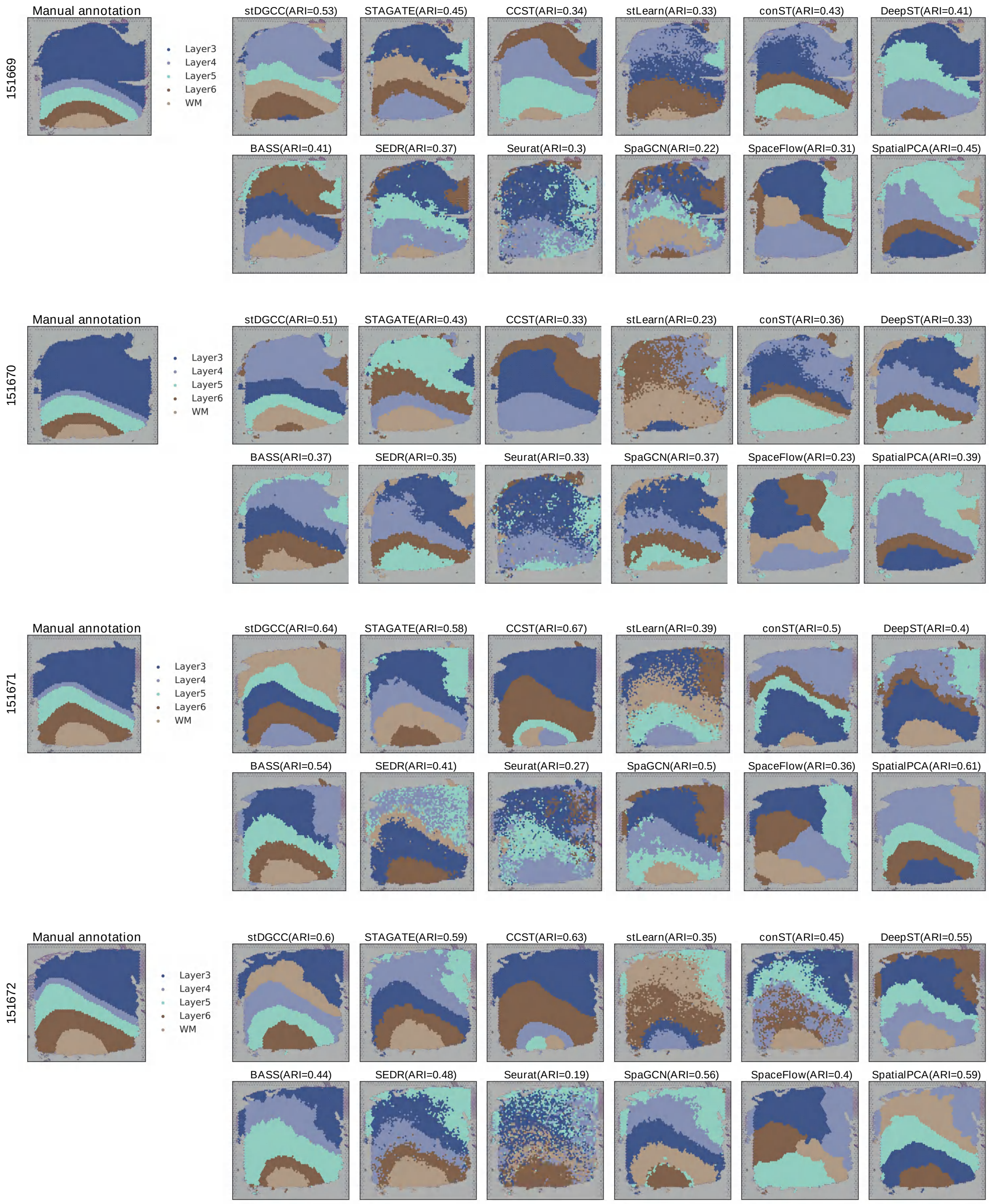

See next page

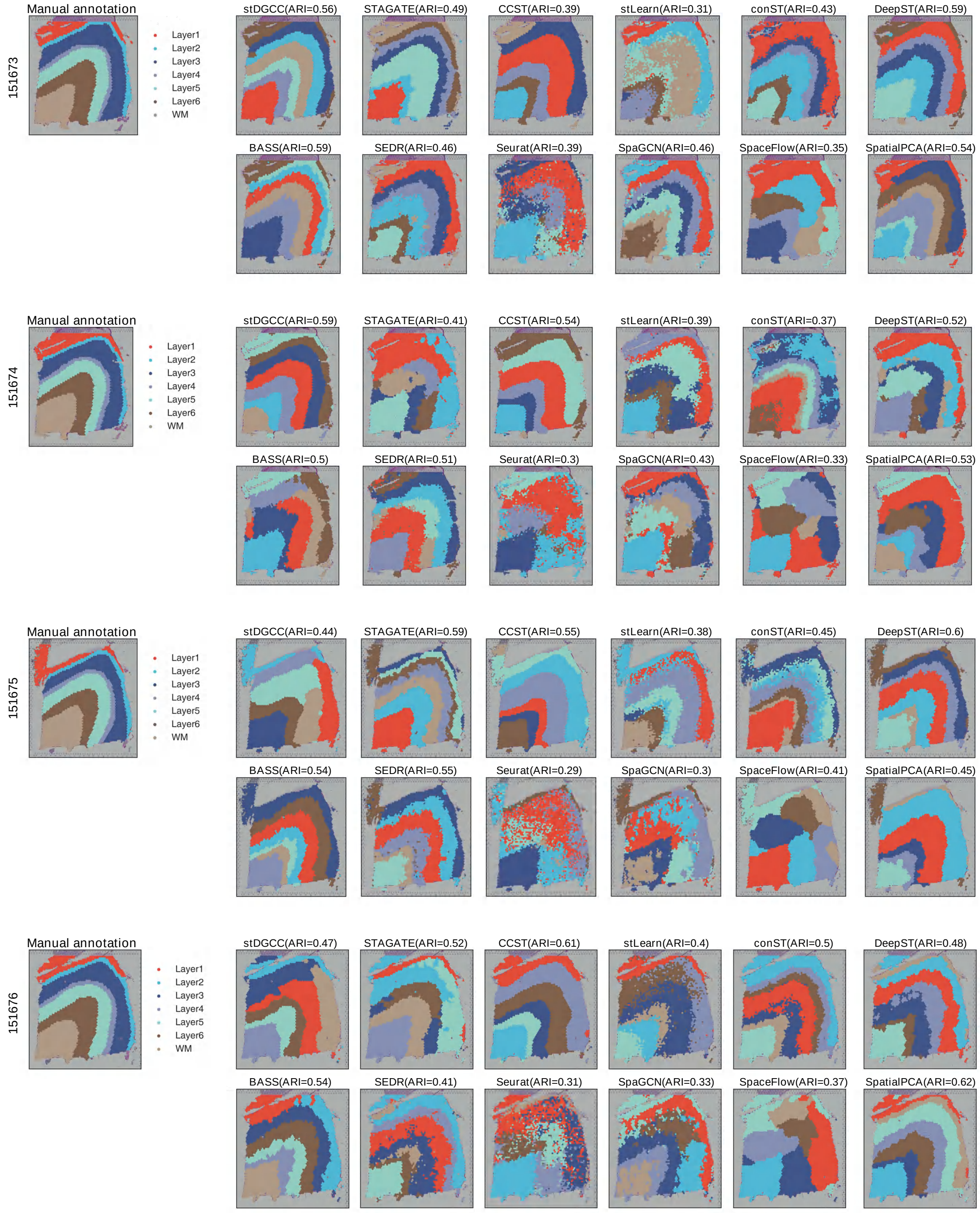

Figure S5: Cluster assignments generated by stDGCC and other baseline methods on 12 DLPFC sections.

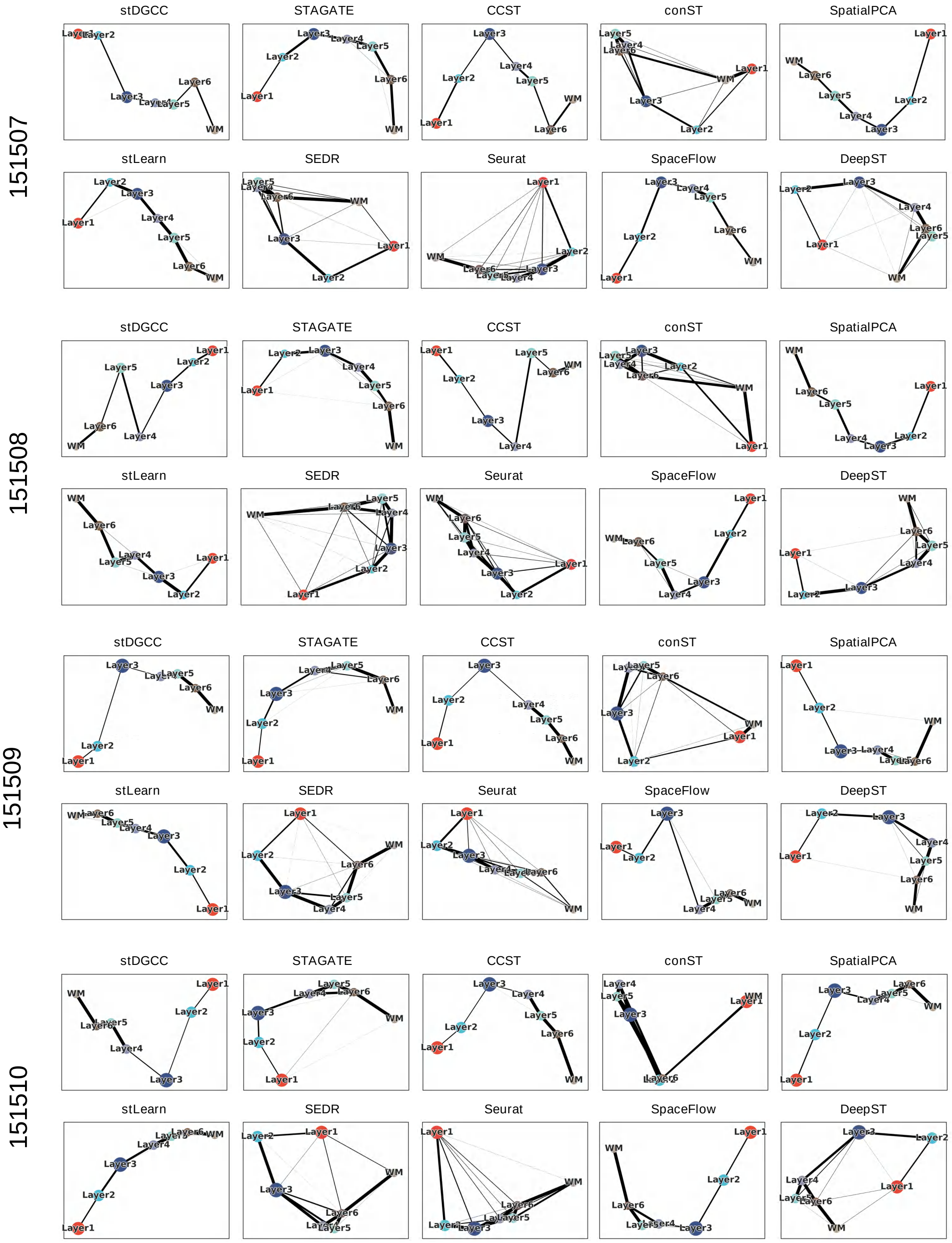

See next page

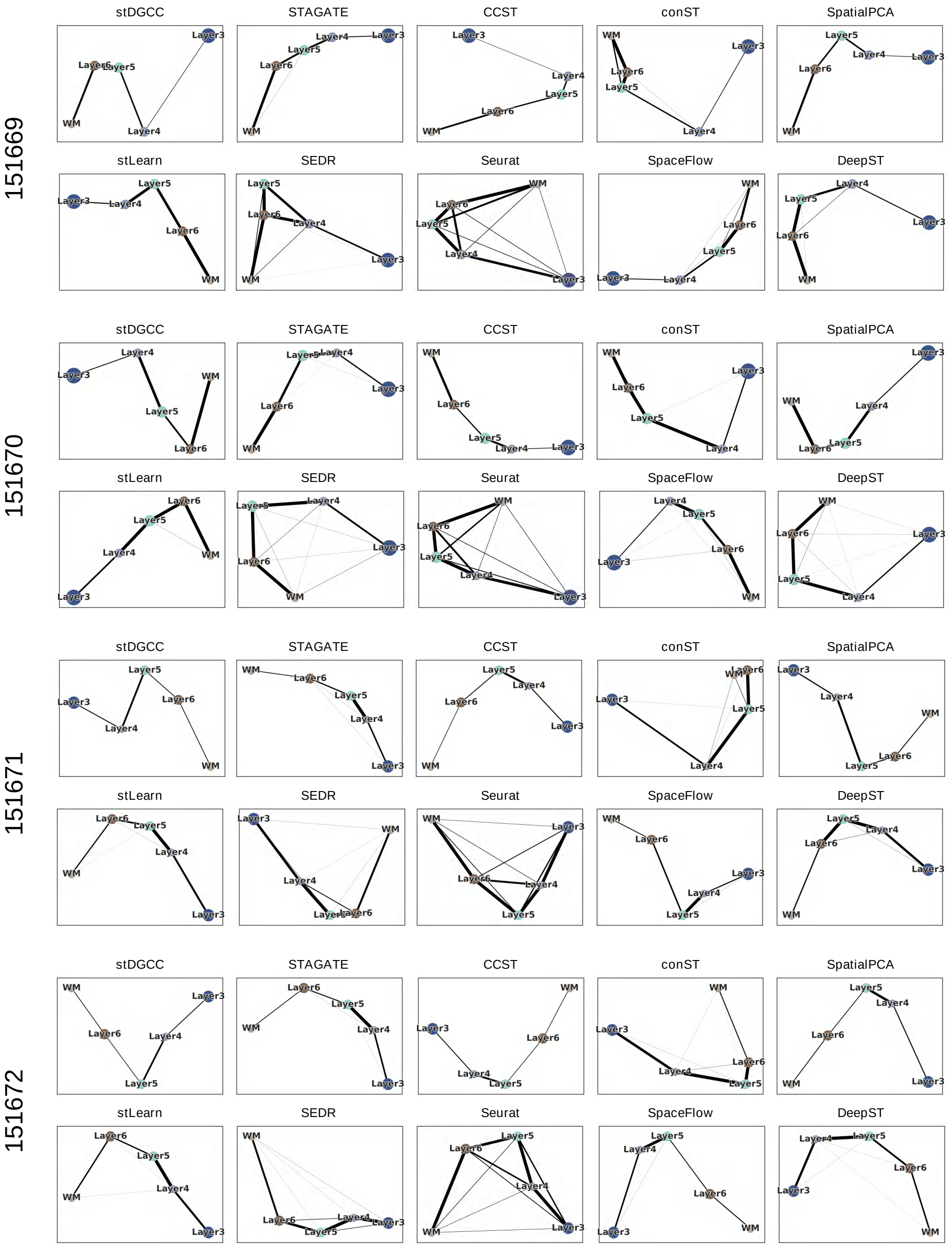

See next page

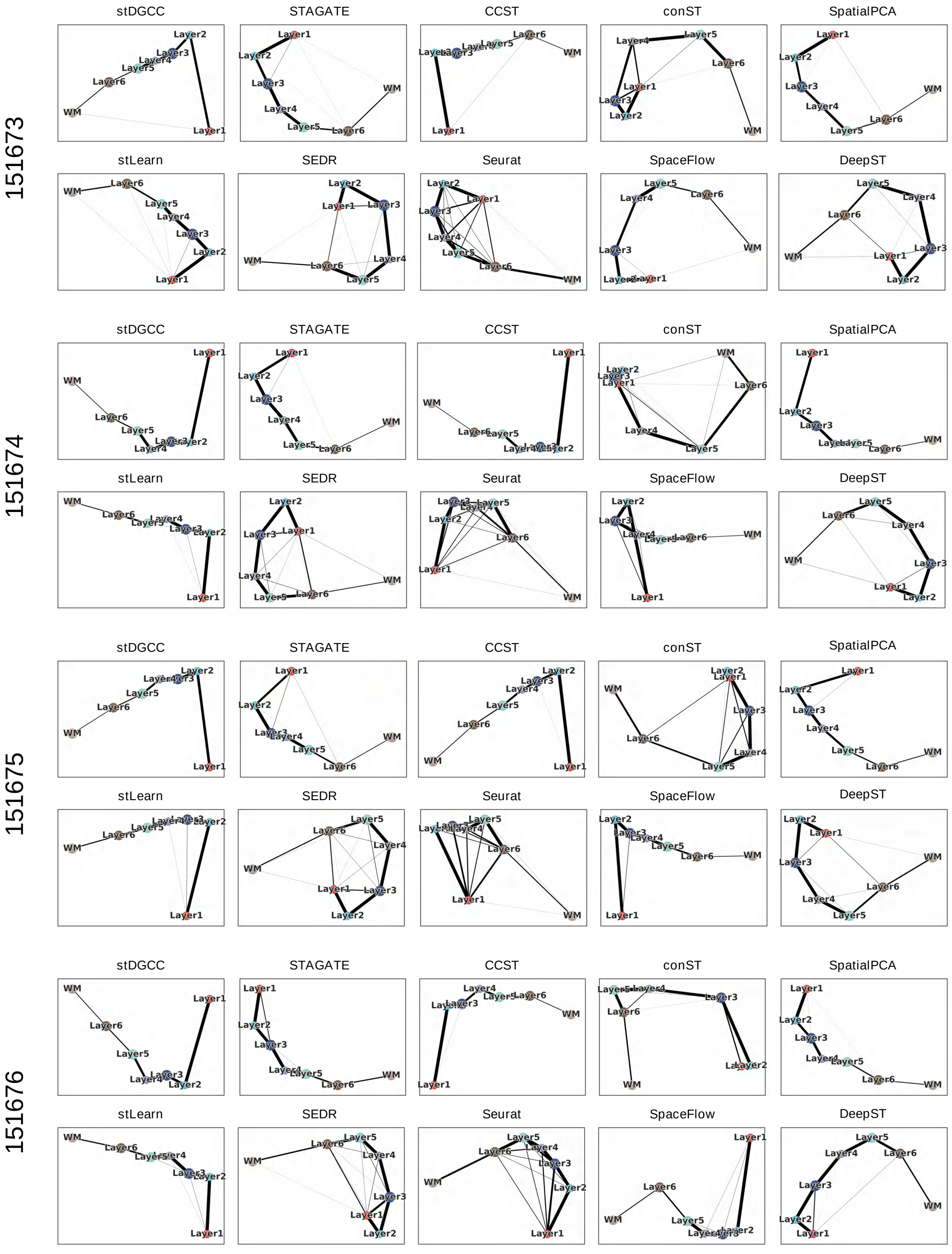

Figure S6: PAGA graphs generated by stDGCC and other algorithm embeddings on 12 DLPFC sections.

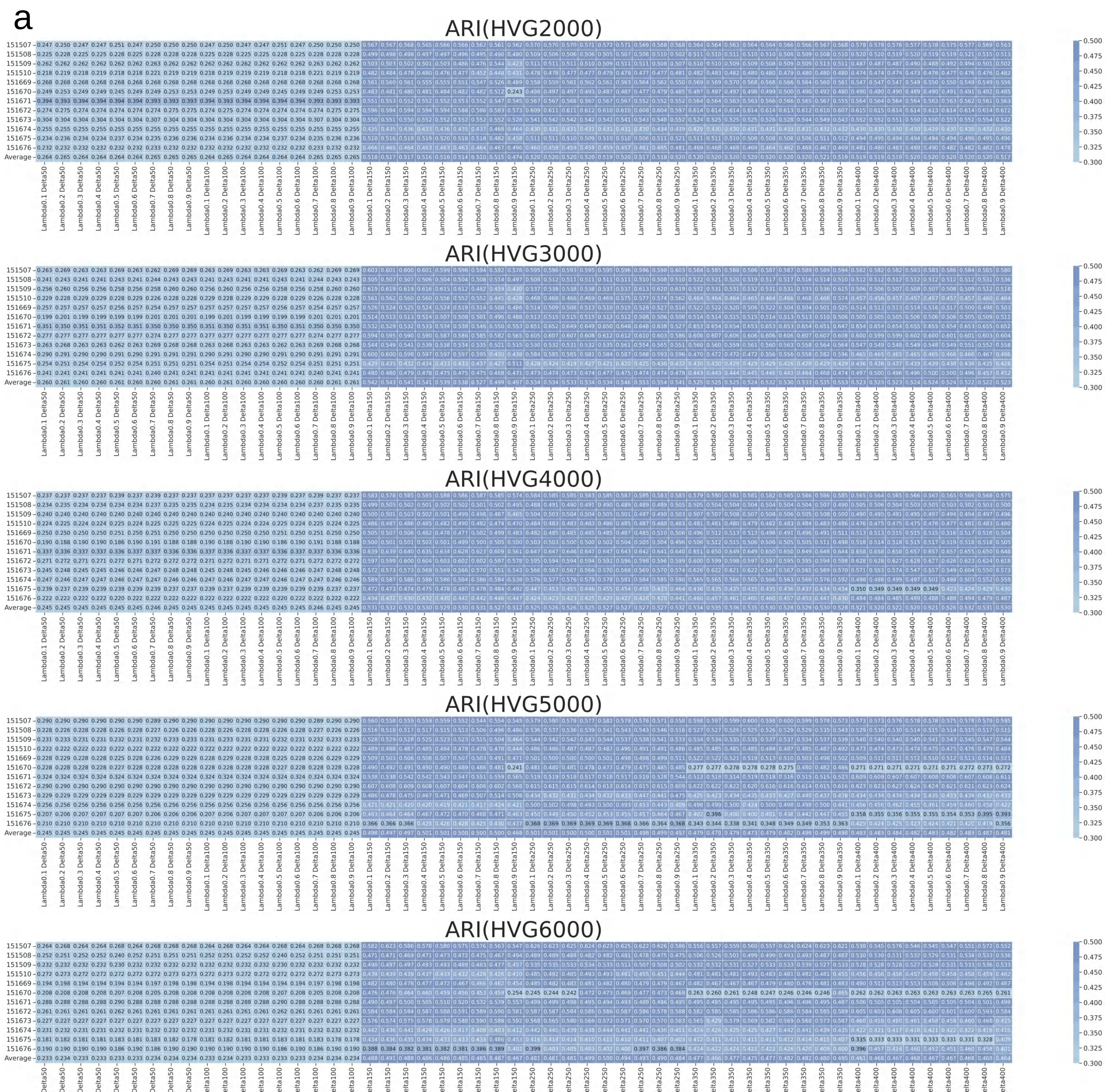

See next page

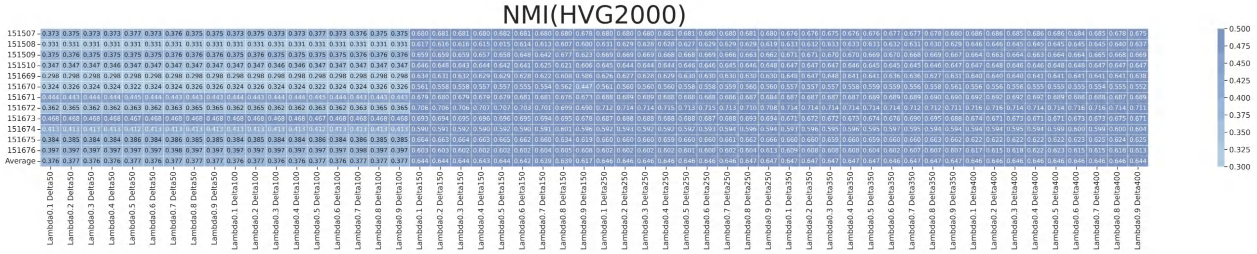

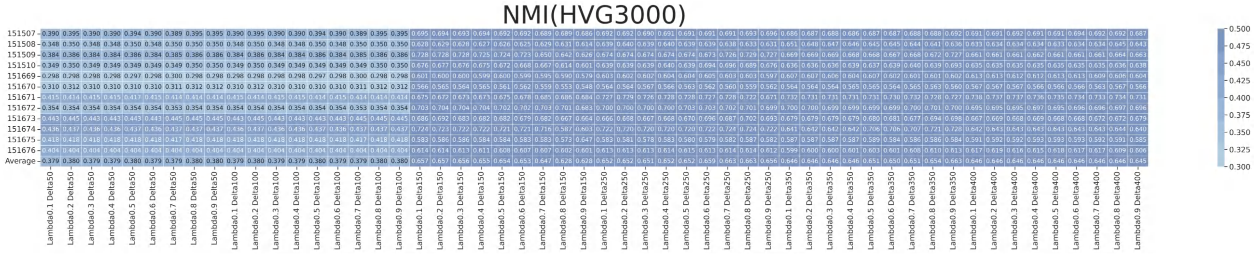

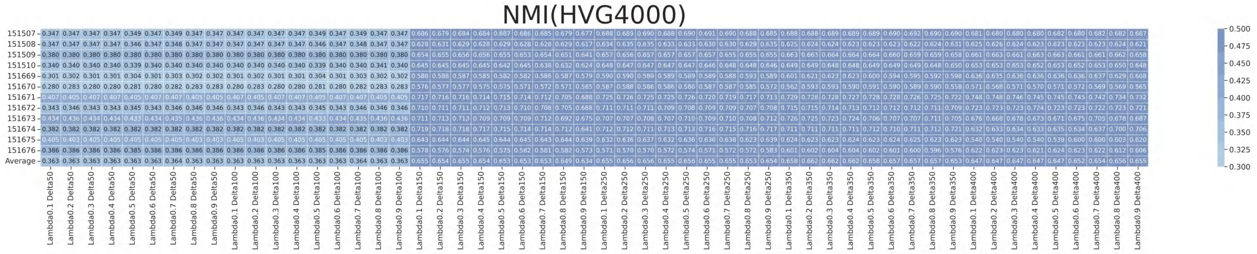

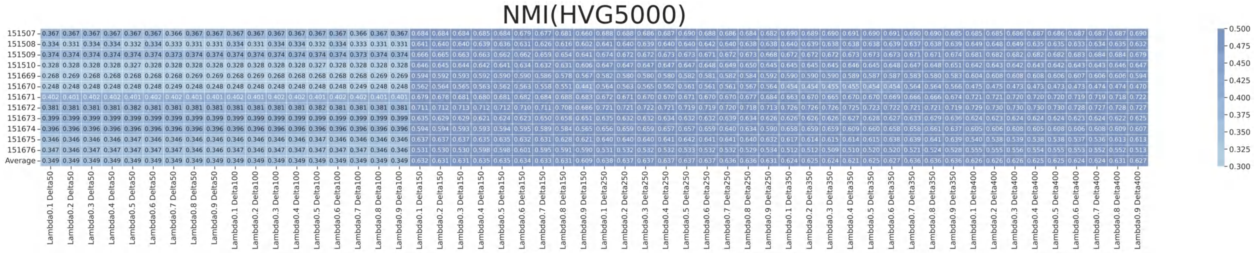

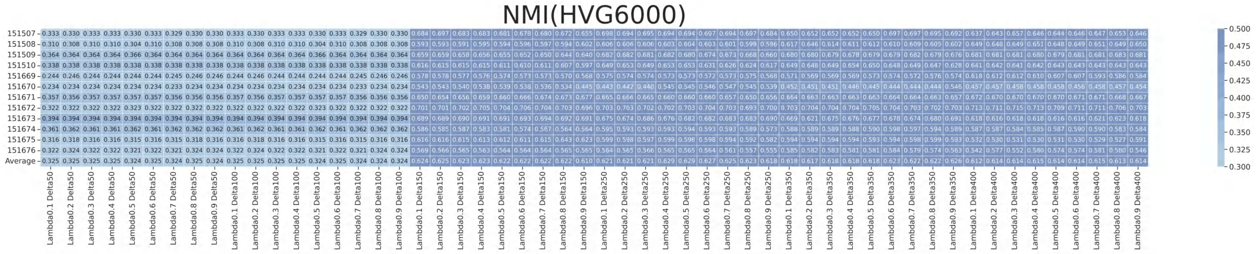

See next page

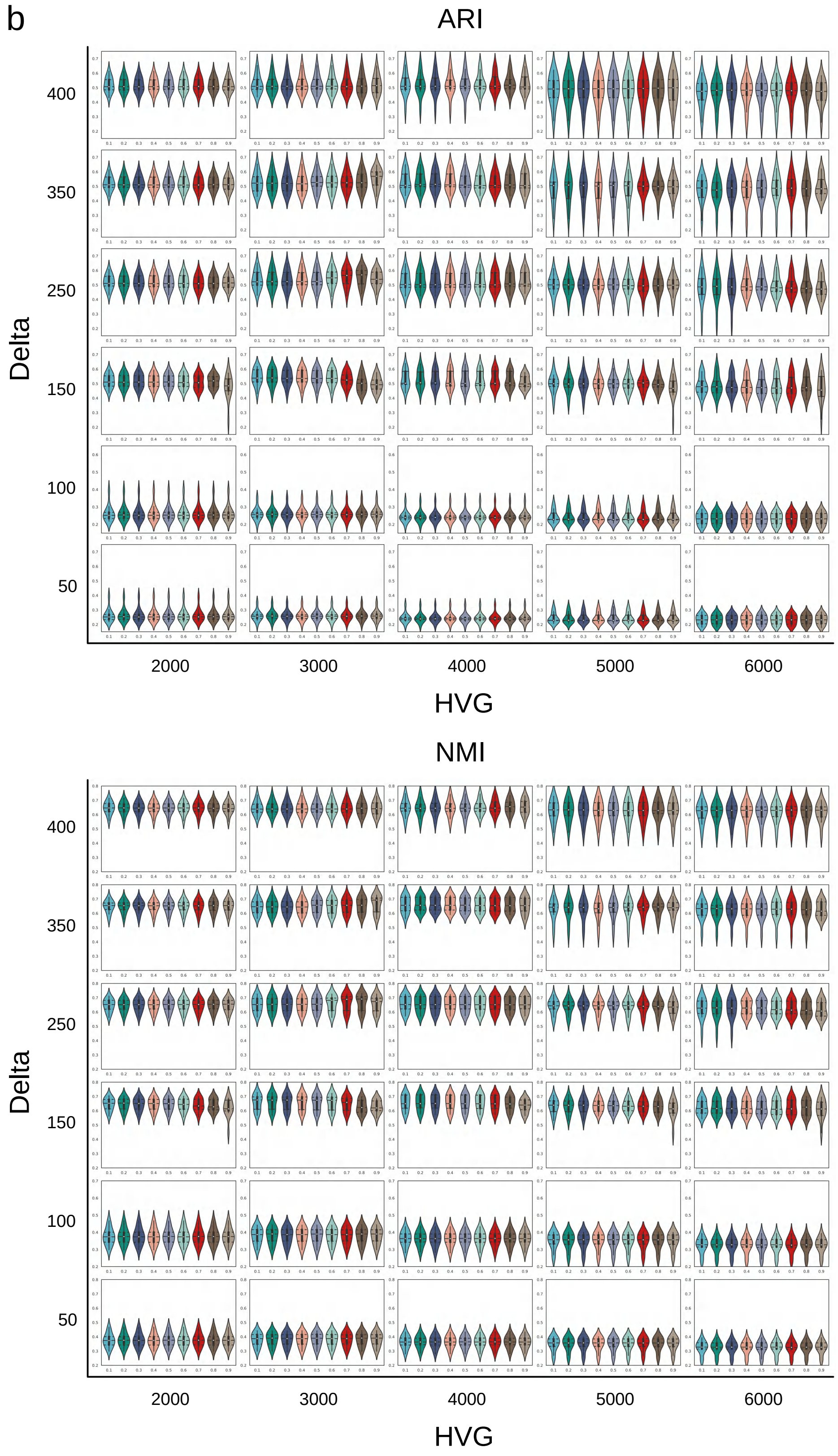

Figure S7: (a) The clustering performance of stDGCC with different combinations of hyperparameters on the DLPFC dataset was evaluated using NMI and ARI as evaluation metrics. (b) The violin plots of clustering performance of stDGCC with different combinations of hyperparameters on the DLPFC dataset were evaluated using NMI and ARI as evaluation metrics.

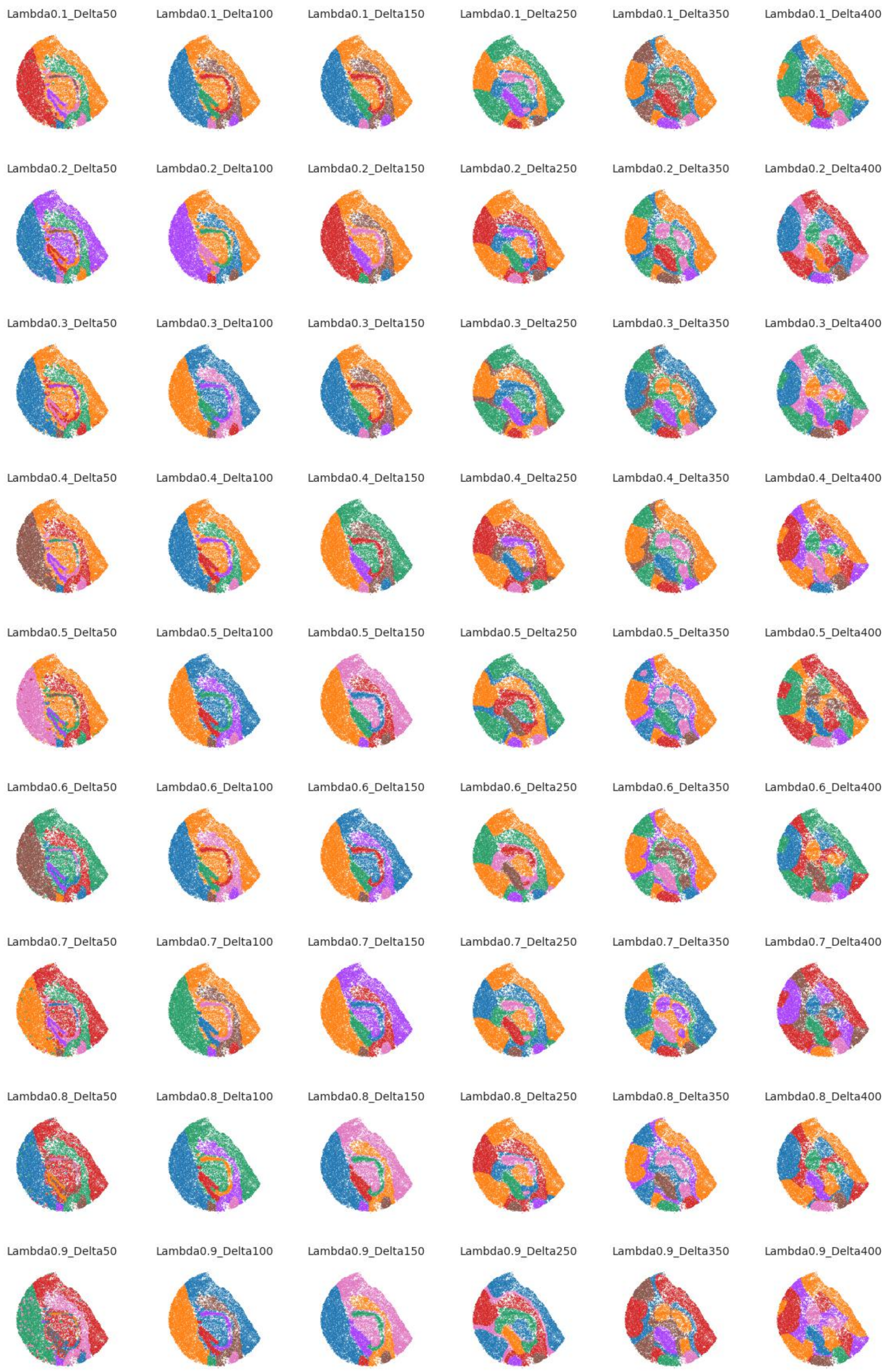

Figure S8: The results of stDGCC with different combinations of λ and δ on the mouse hippocampus dataset profiled by Slide-seq when the number of highly variable genes is set to 2000

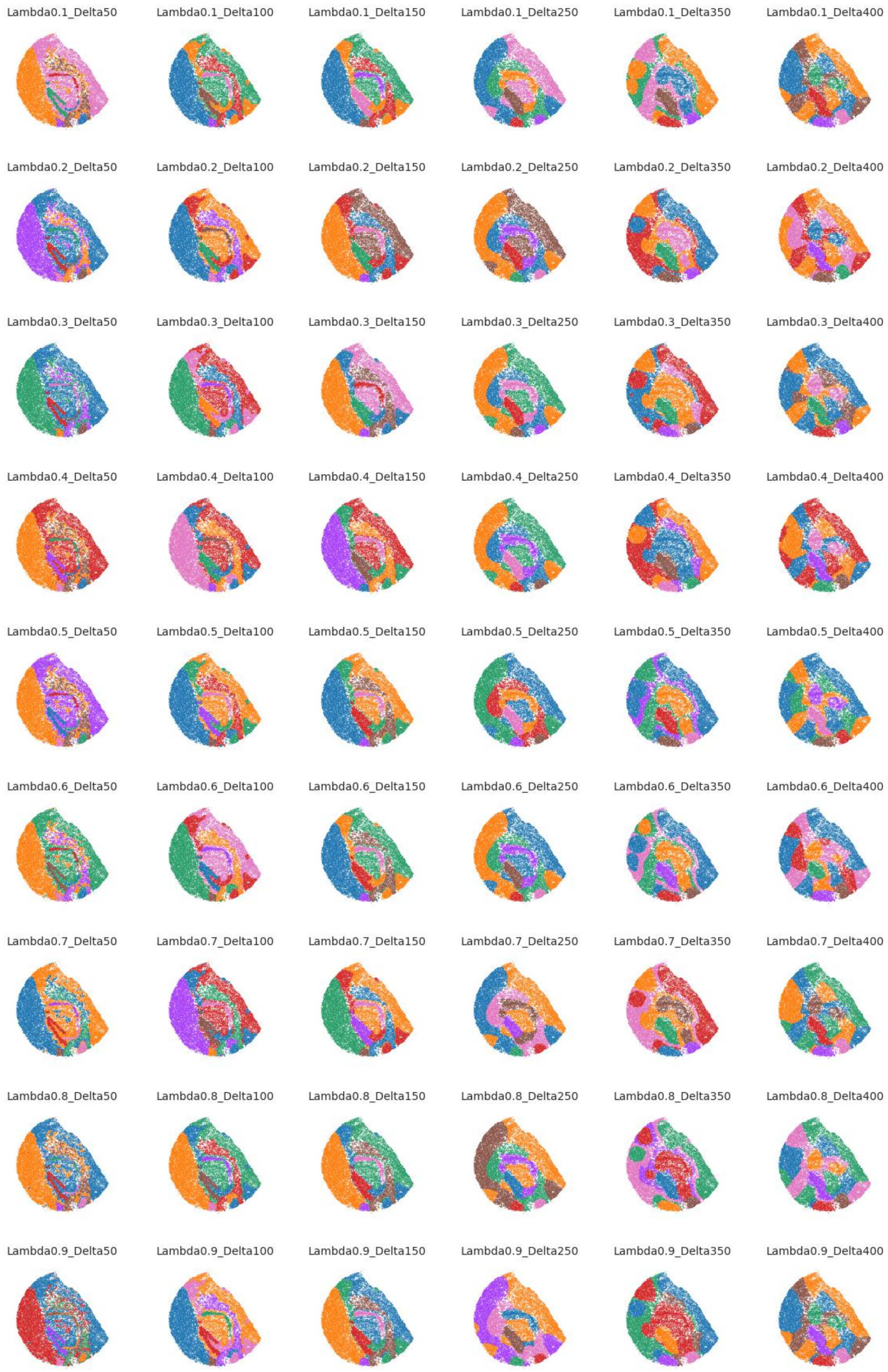

Figure S9: The results of stDGCC with different combinations of λ and δ on the mouse hippocampus dataset profiled by Slide-seq when the number of highly variable genes is set to 3000

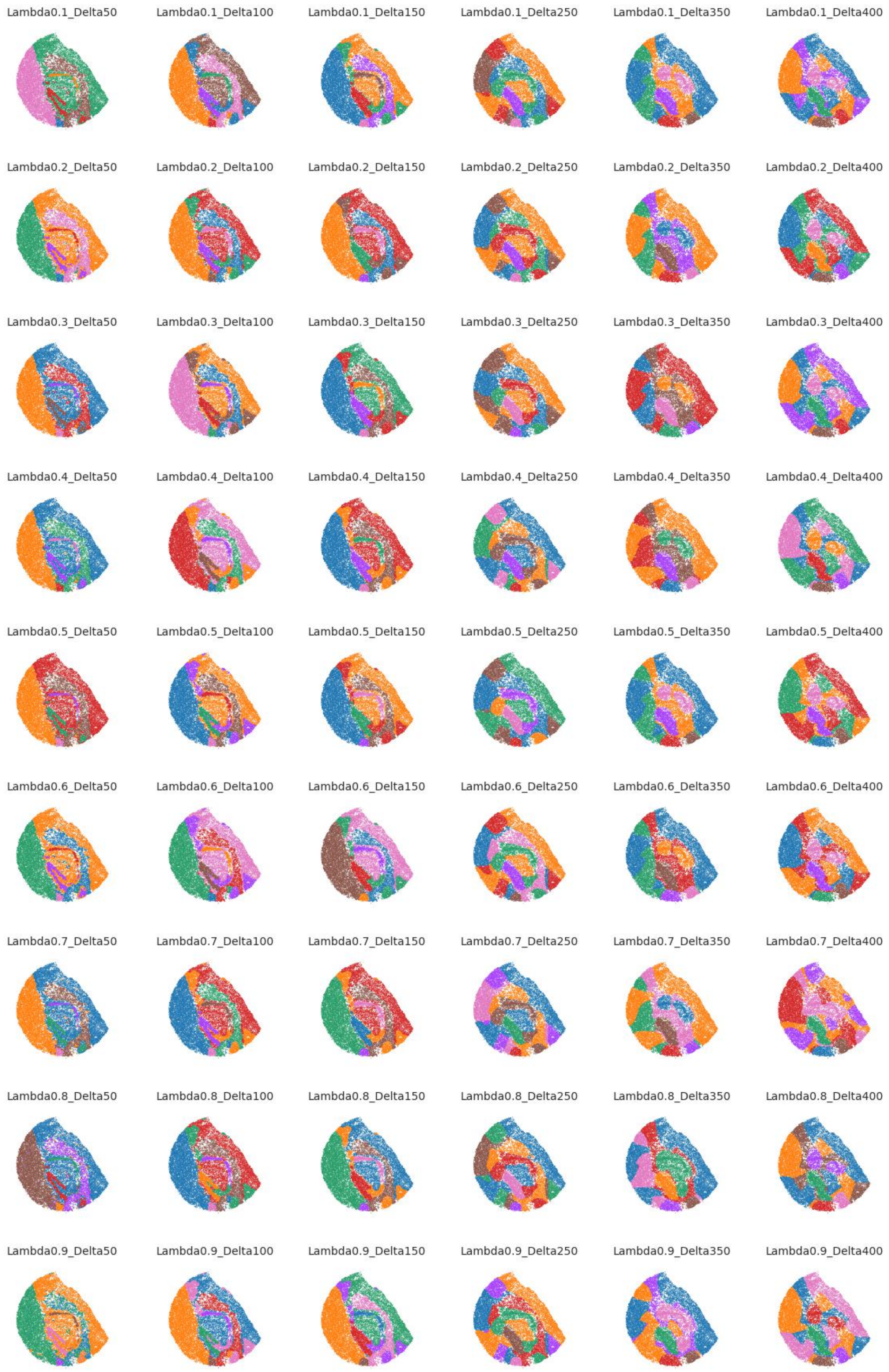

Figure S10: The results of stDGCC with different combinations of λ and δ on the mouse hippocampus dataset profiled by Slide-seq when the number of highly variable genes is set to 4000

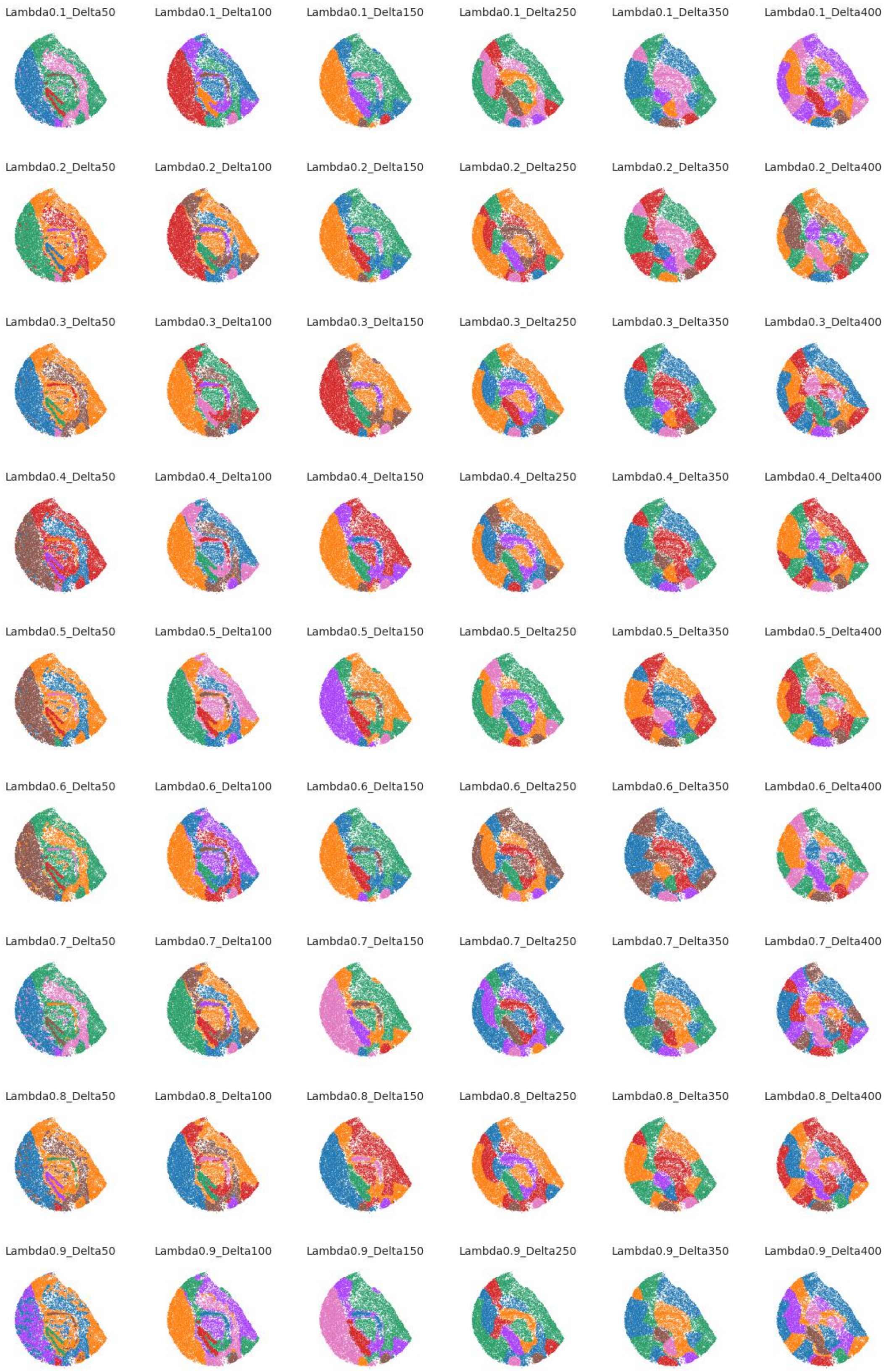

Figure S11: The results of stDGCC with different combinations of λ and δ on the mouse hippocampus dataset profiled by Slide-seq when the number of highly variable genes is set to 5000

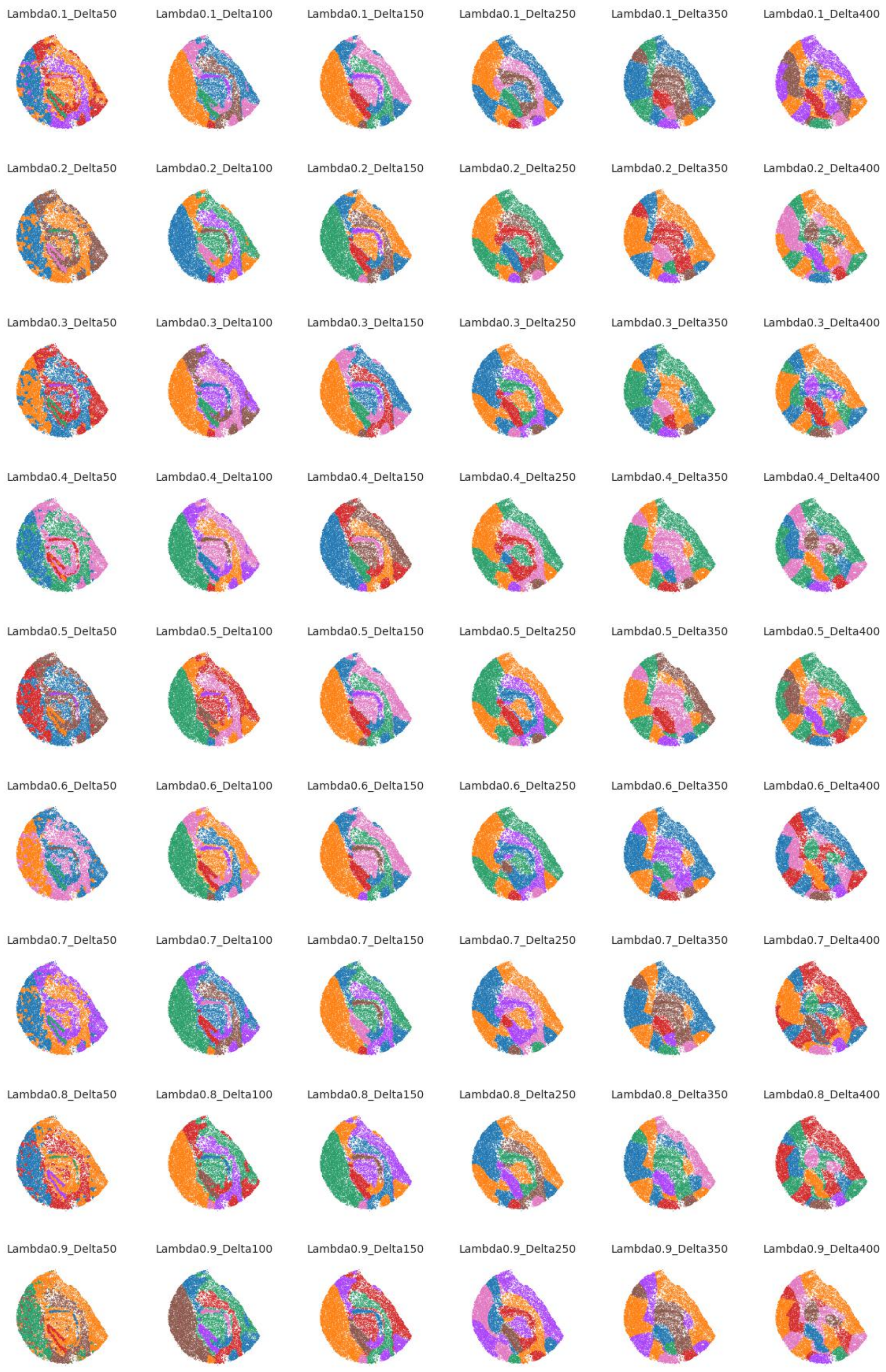

Figure S12: The results of stDGCC with different combinations of λ and δ on the mouse hippocampus dataset profiled by Slide-seq when the number of highly variable genes is set to 6000

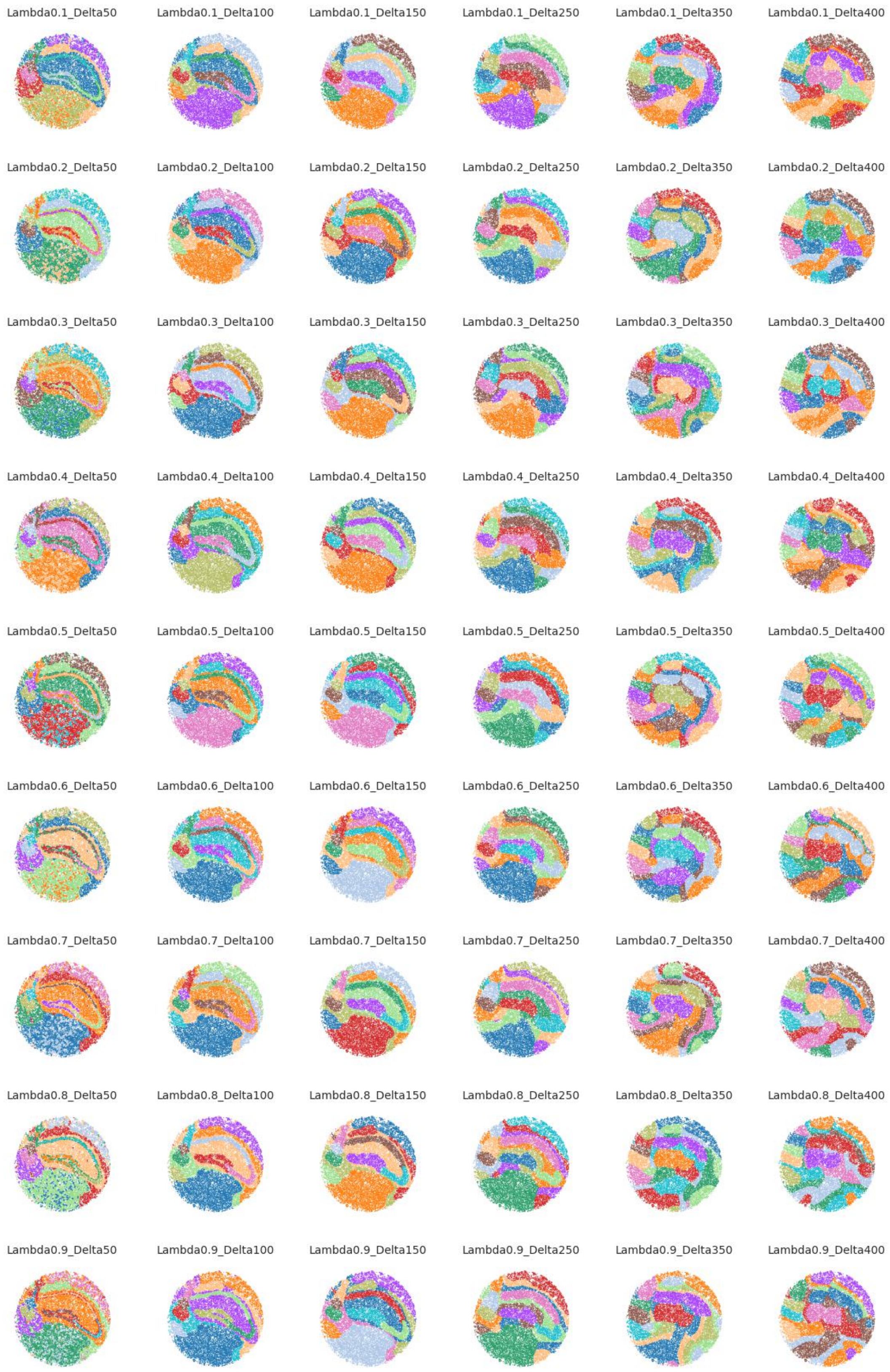

Figure S13: The results of stDGCC with different combinations of λ and δ on the mouse hippocampus dataset profiled by Slide-seqV2 when the number of highly variable genes is set to 2000

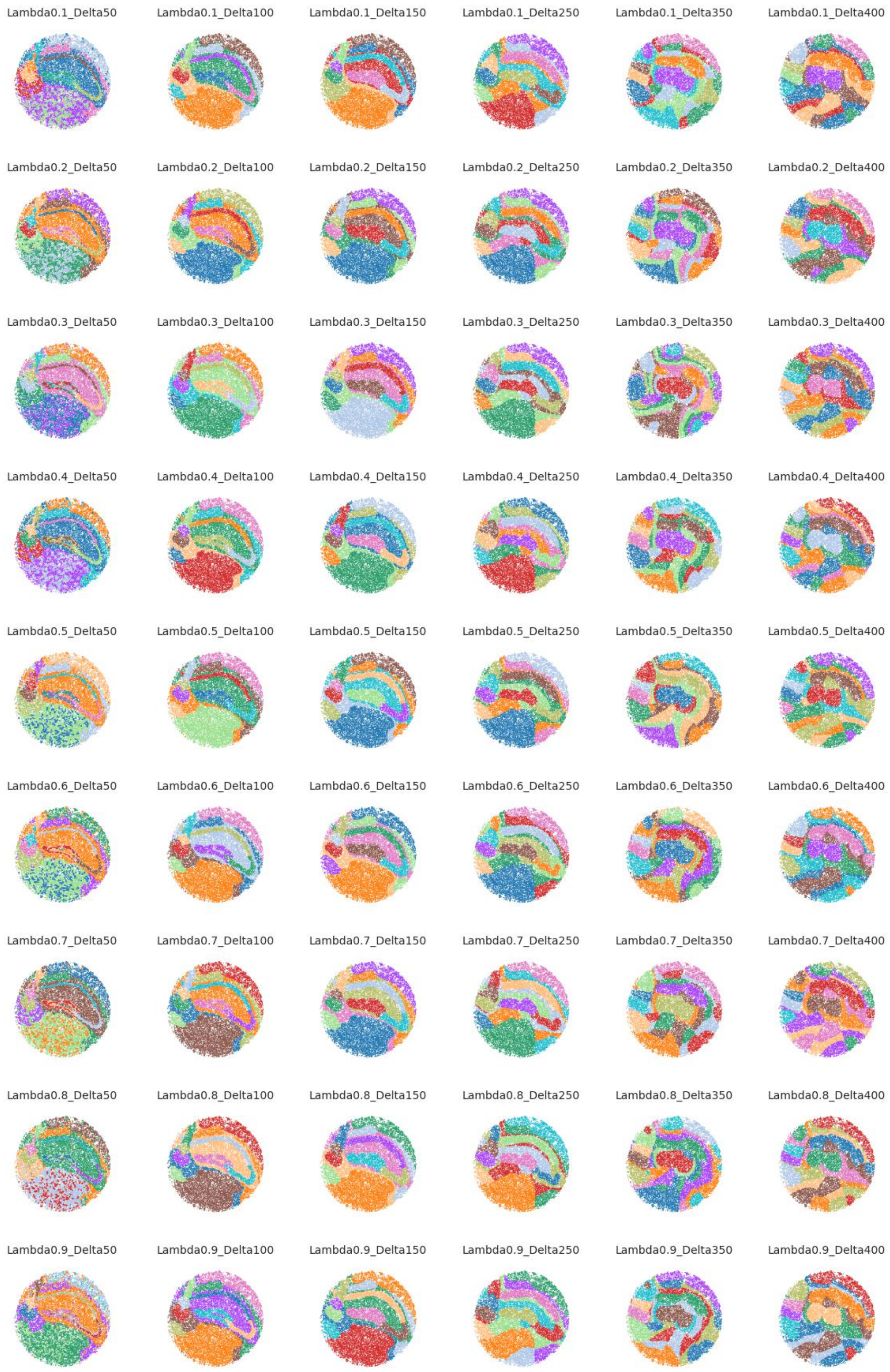

Figure S14: The results of stDGCC with different combinations of λ and δ on the mouse hippocampus dataset profiled by Slide-seqV2 when the number of highly variable genes is set to 3000

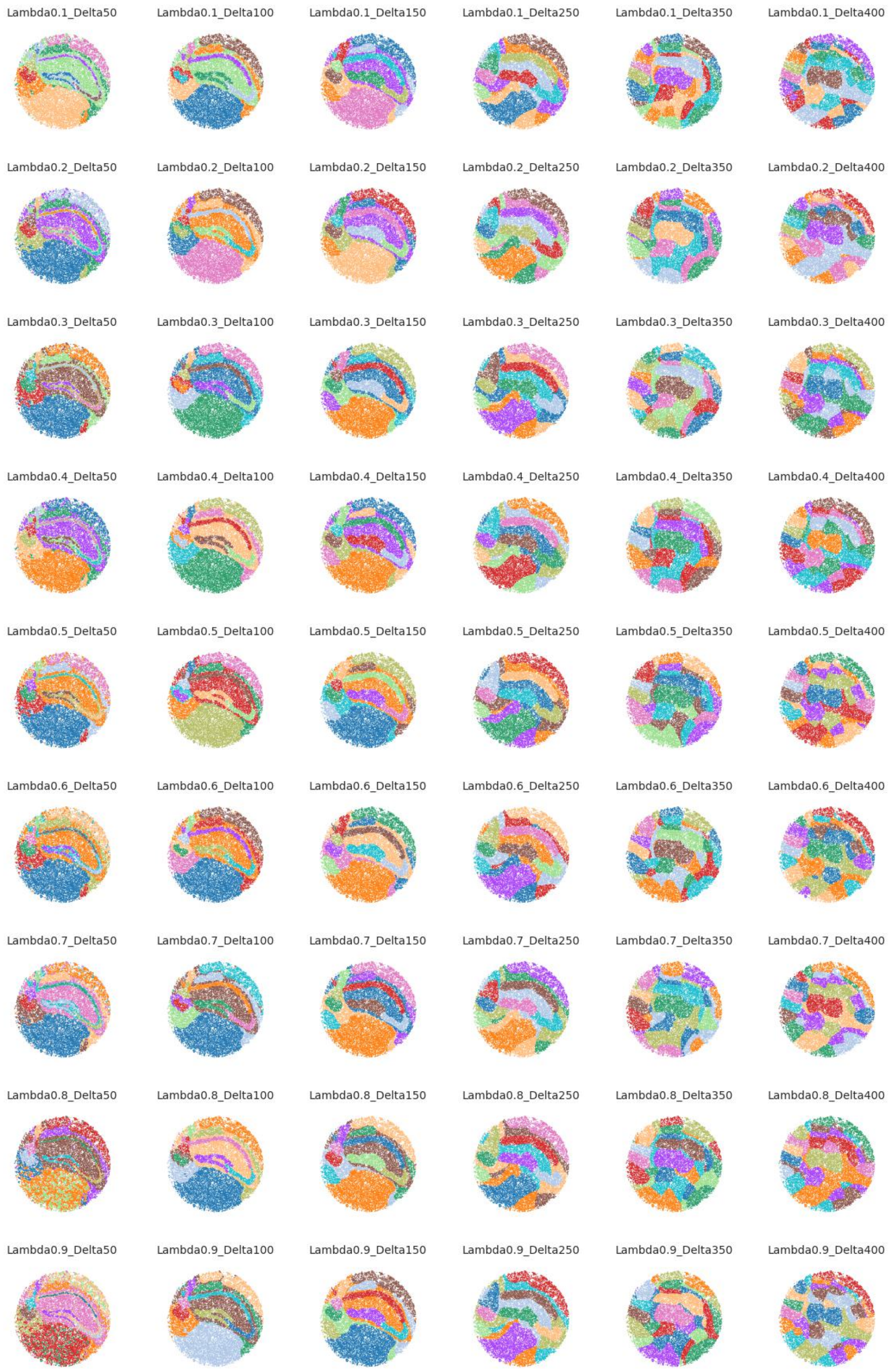

Figure S15: The results of stDGCC with different combinations of λ and δ on the mouse hippocampus dataset profiled by Slide-seqV2 when the number of highly variable genes is set to 4000

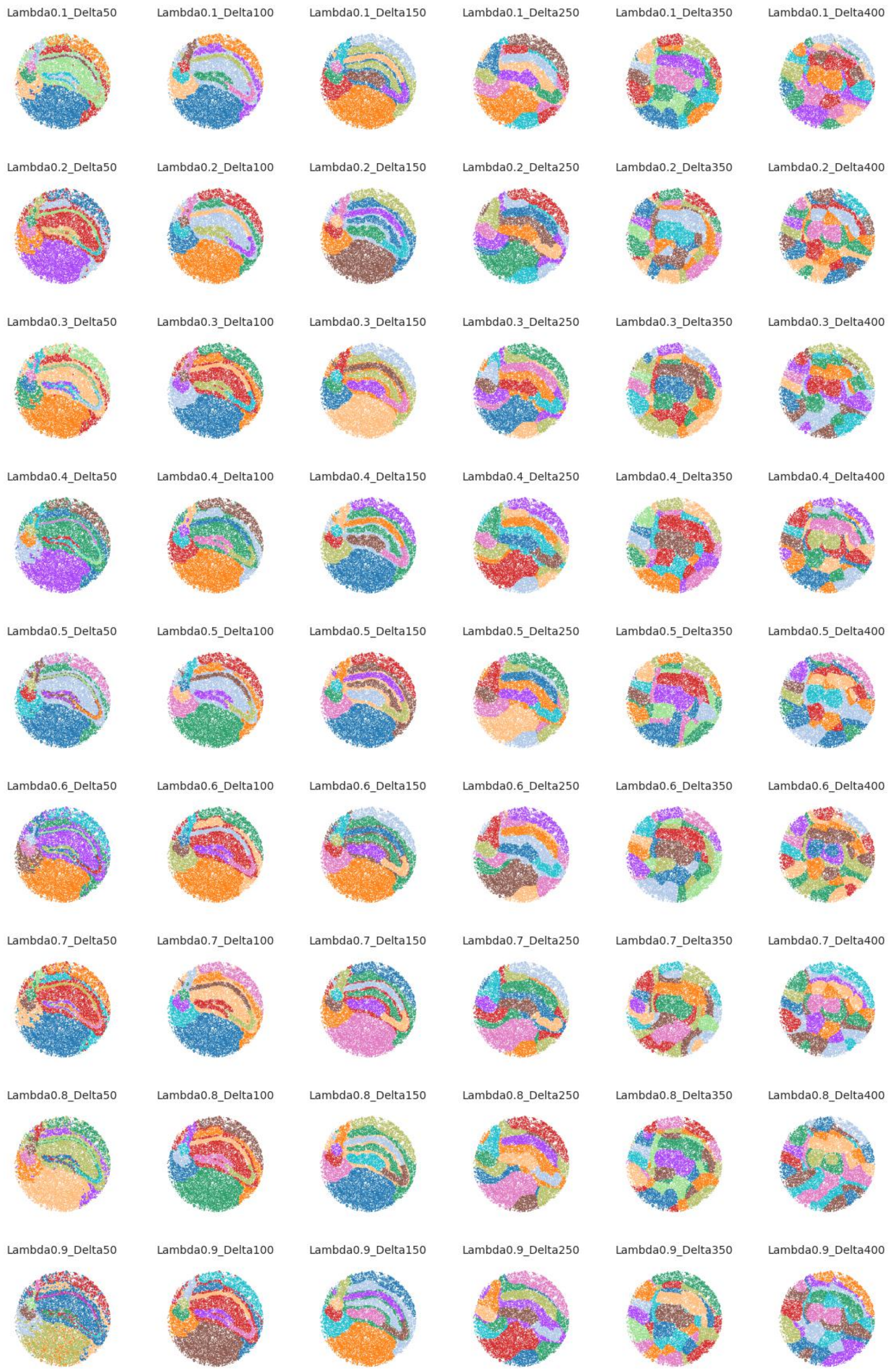

Figure S16: The results of stDGCC with different combinations of λ and δ on the mouse hippocampus dataset profiled by Slide-seqV2 when the number of highly variable genes is set to 5000

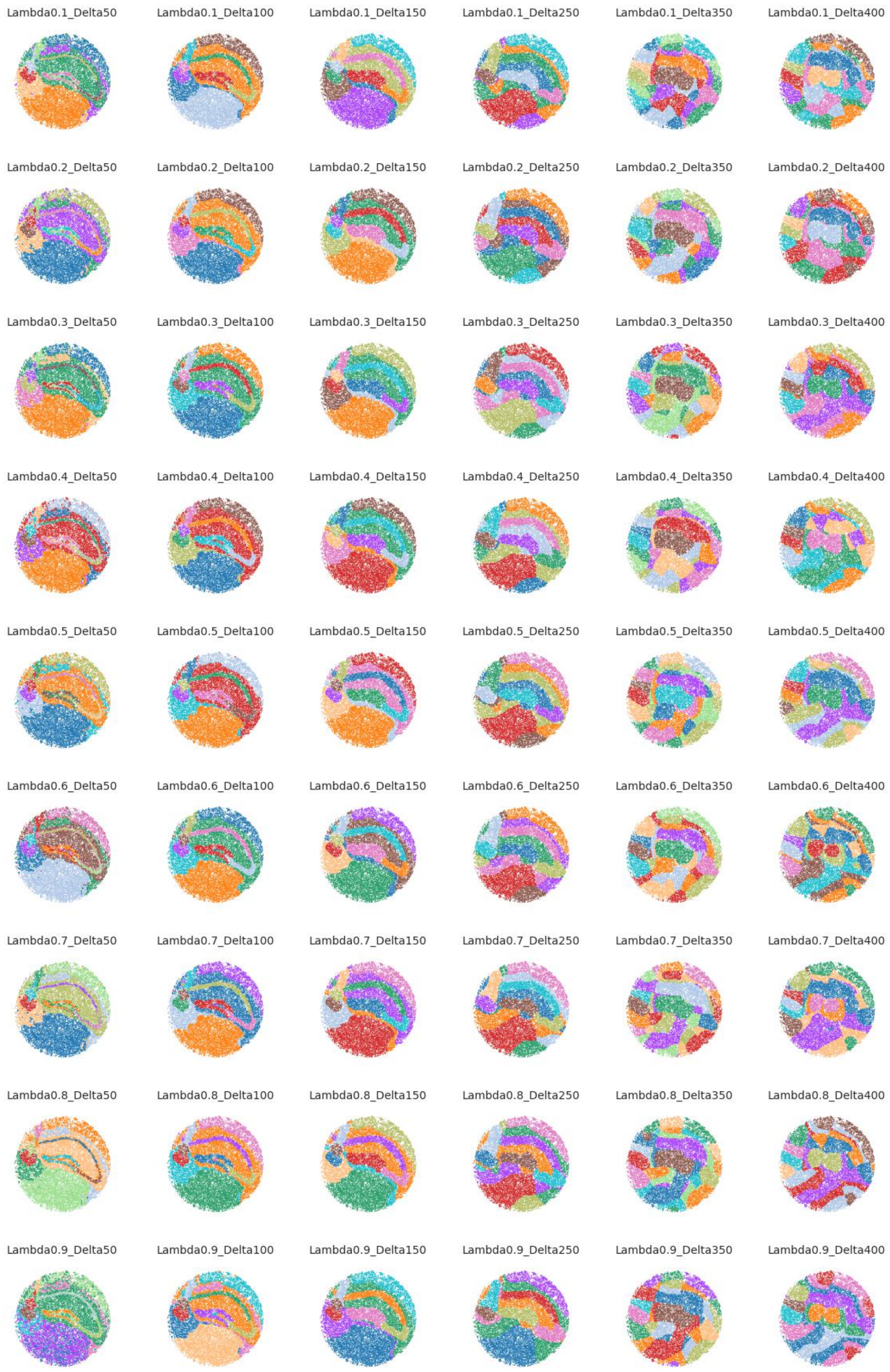

Figure S17: The results of stDGCC with different combinations of λ and δ on the mouse hippocampus dataset profiled by Slide-seqV2 when the number of highly variable genes is set to 6000

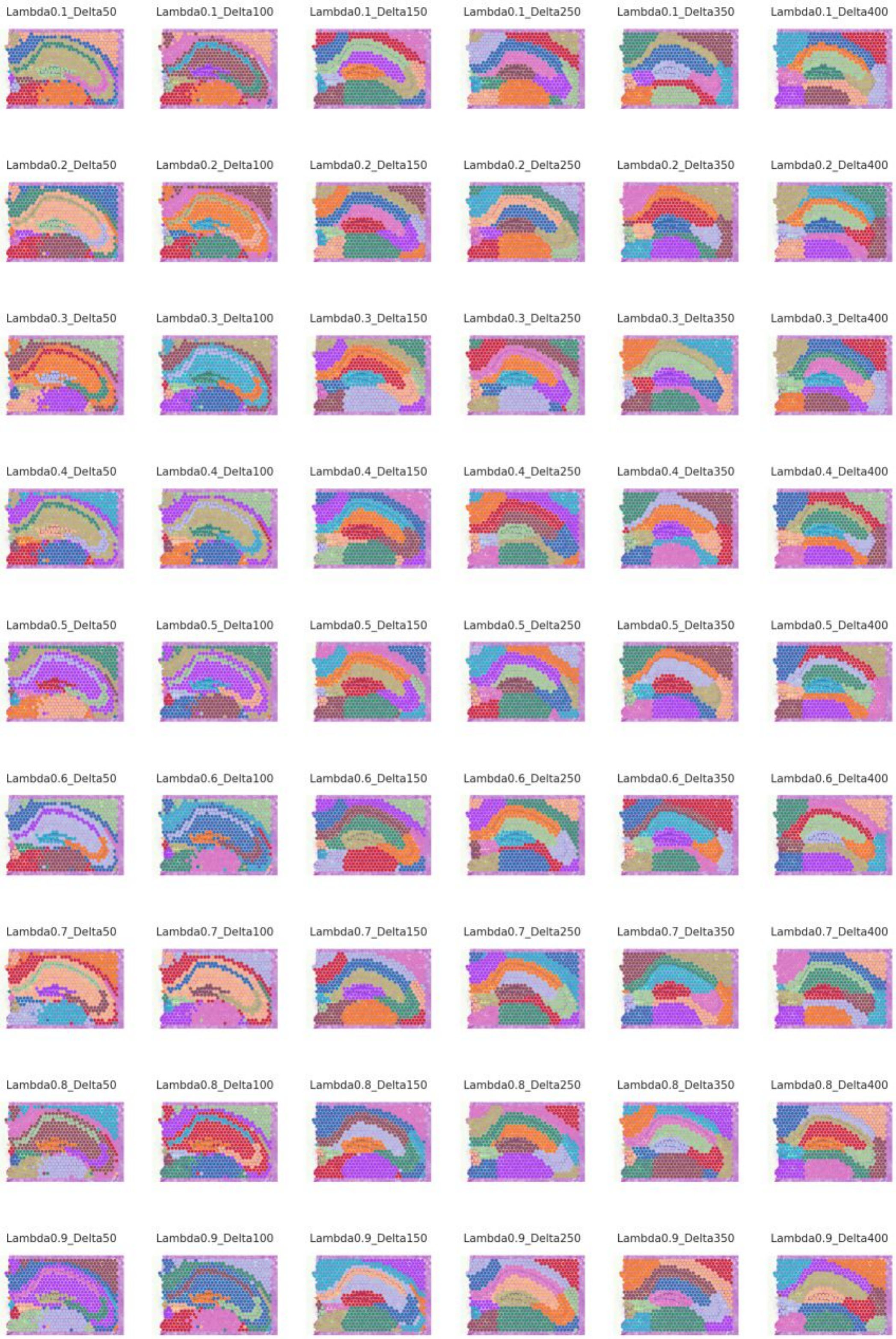

Figure S18: The results of stDGCC with different combinations of λ and δ on the mouse brain dataset profiled by 10x Visium when the number of highly variable genes is set to 2000

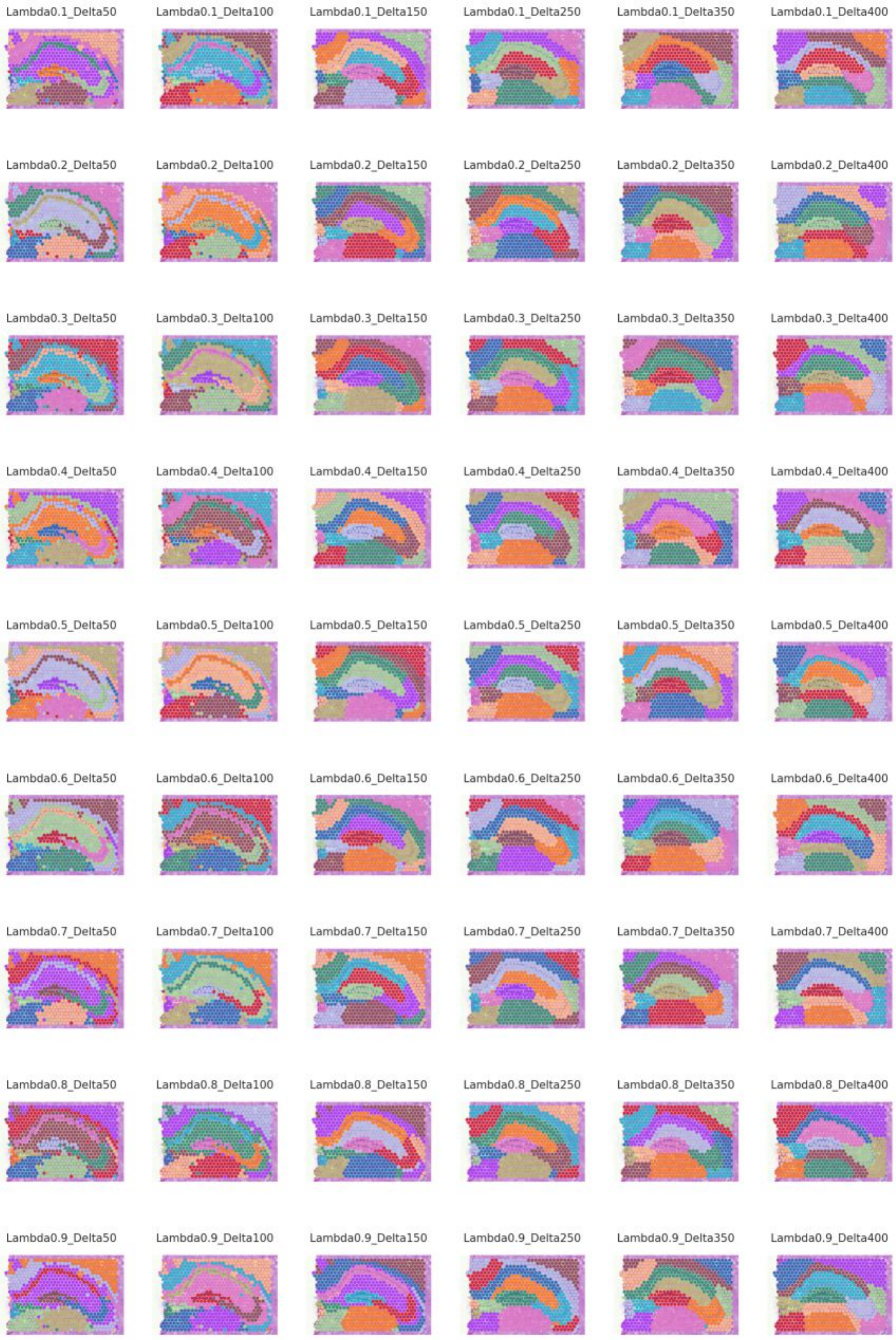

Figure S19: The results of stDGCC with different combinations of λ and δ on the mouse brain dataset profiled by 10x Visium when the number of highly variable genes is set to 3000

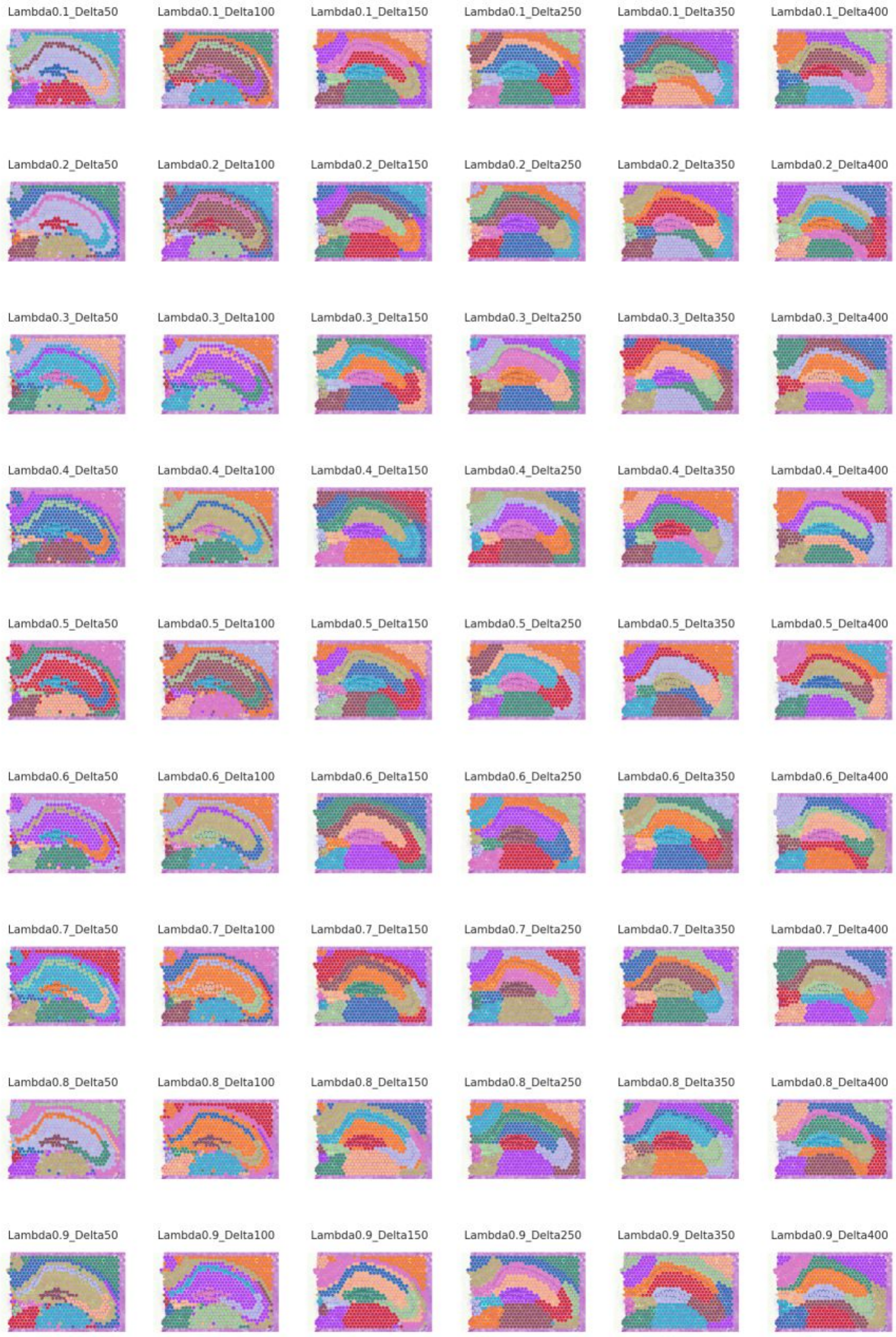

Figure S20: The results of stDGCC with different combinations of λ and δ on the mouse brain dataset profiled by 10x Visium when the number of highly variable genes is set to 4000

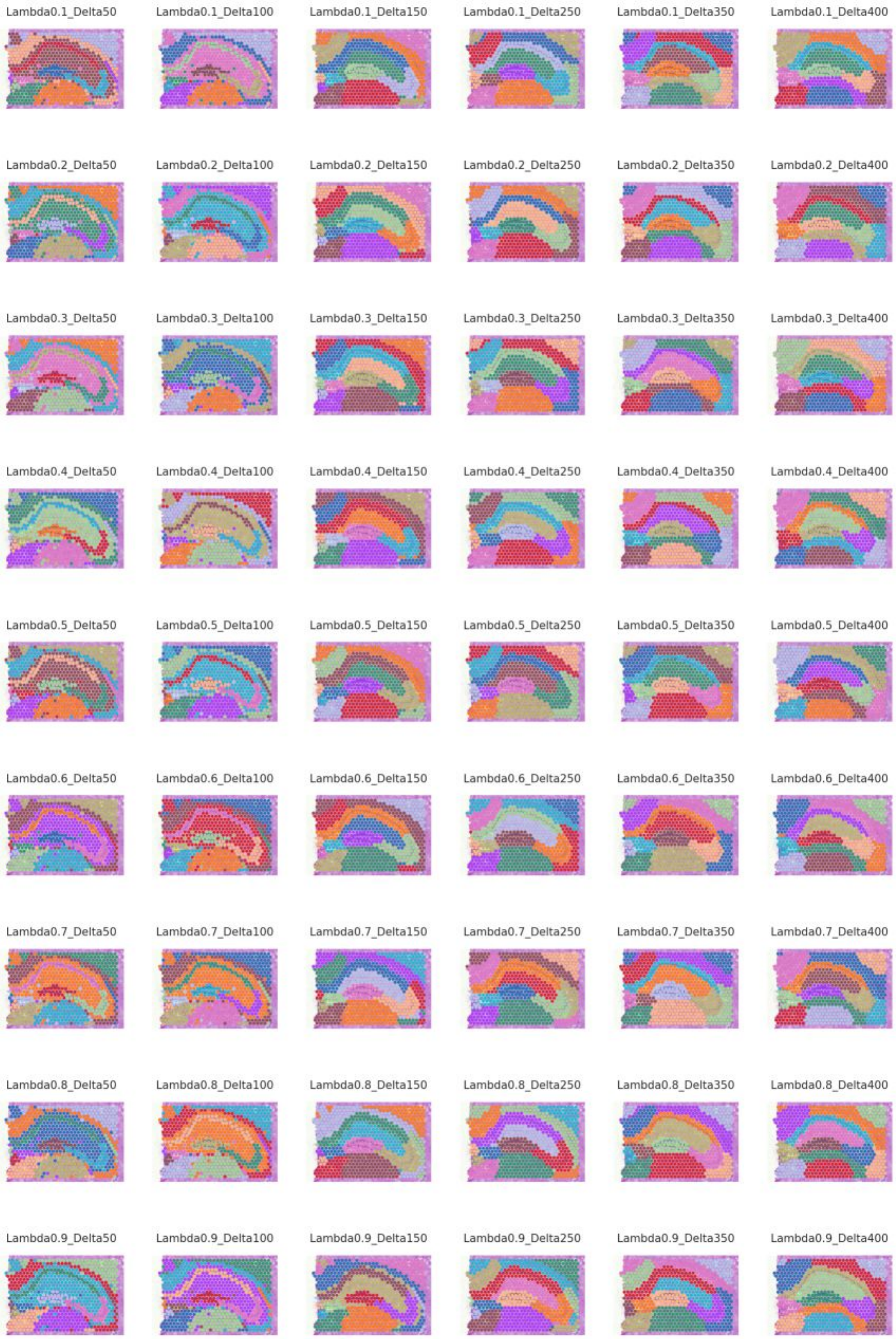

Figure S21: The results of stDGCC with different combinations of λ and δ on the mouse brain dataset profiled by 10x Visium when the number of highly variable genes is set to 5000

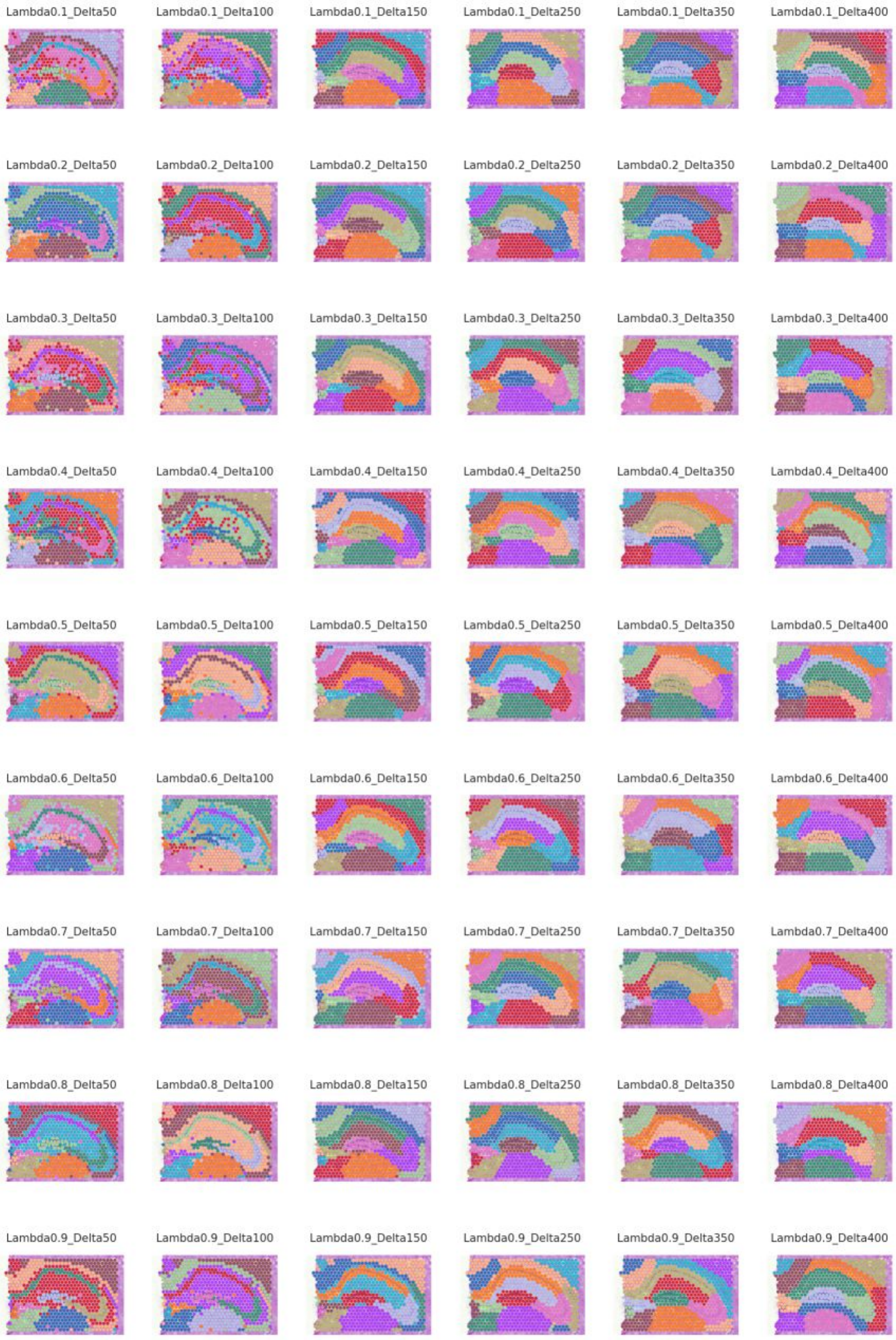

Figure S22: The results of stDGCC with different combinations of λ and δ on the mouse brain dataset profiled by 10x Visium when the number of highly variable genes is set to 6000

Lambda 0.1 Delta 50

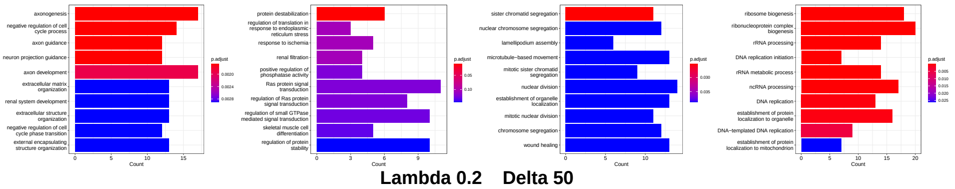

Lambda 0.2 Delta 50

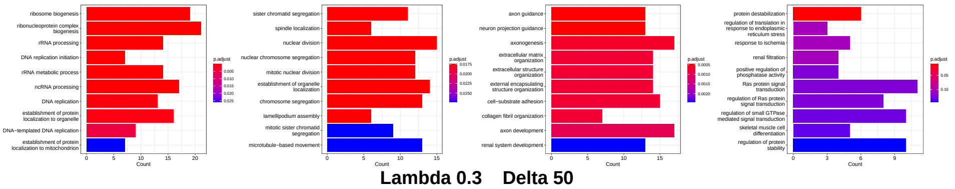

Lambda 0.3 Delta 50

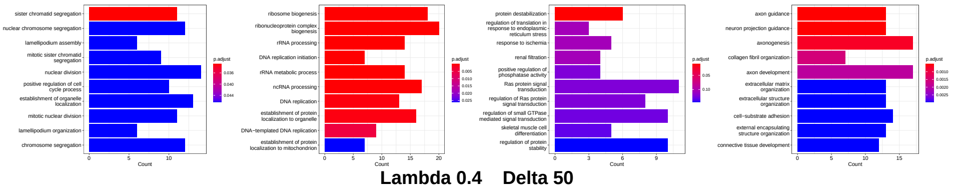

Lambda 0.4 Delta 50

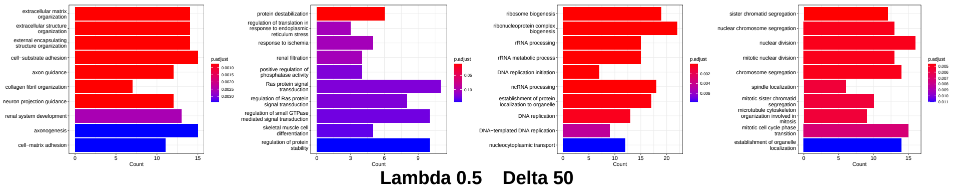

Lambda 0.5 Delta 50

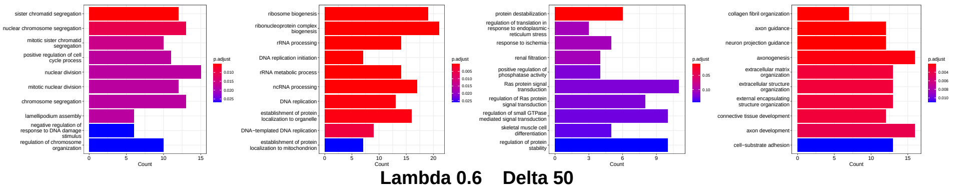

Lambda 0.6 Delta 50

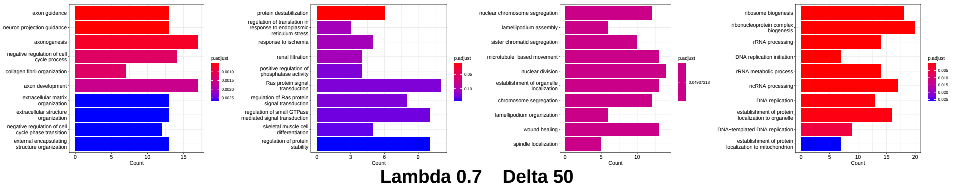

Lambda 0.7 Delta 50

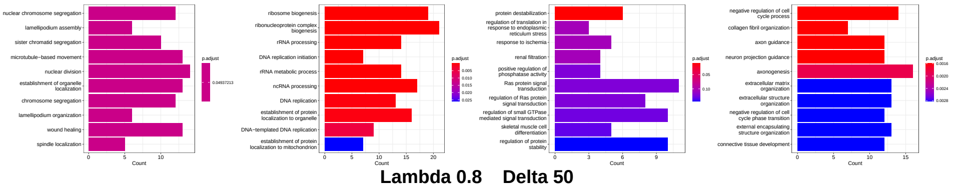

Lambda 0.8 Delta 50

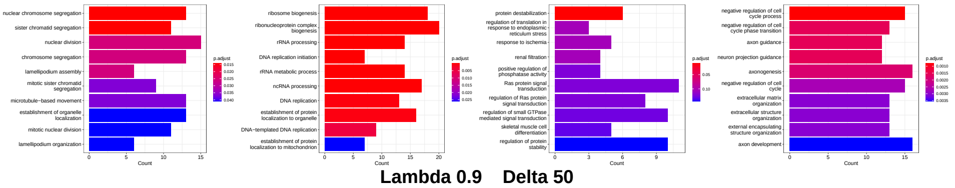

Lambda 0.9 Delta 50

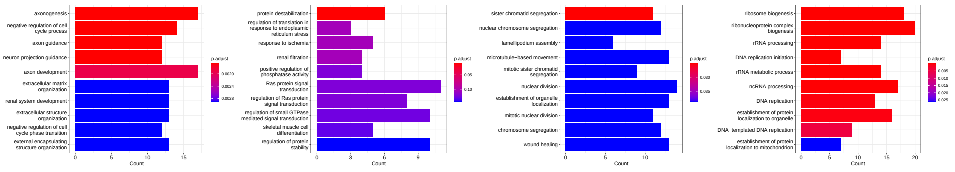

See next page

Lambda 0.1 Delta 100

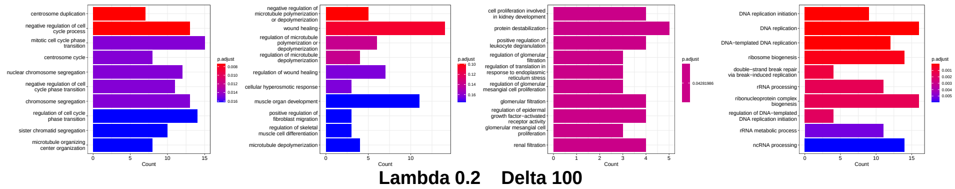

Lambda 0.2 Delta 100

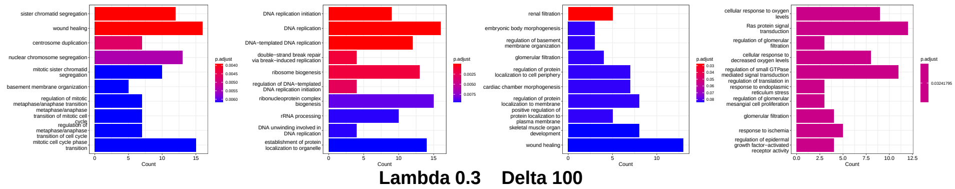

Lambda 0.3 Delta 100

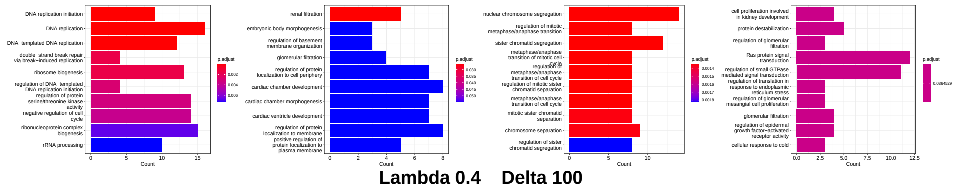

Lambda 0.4 Delta 100

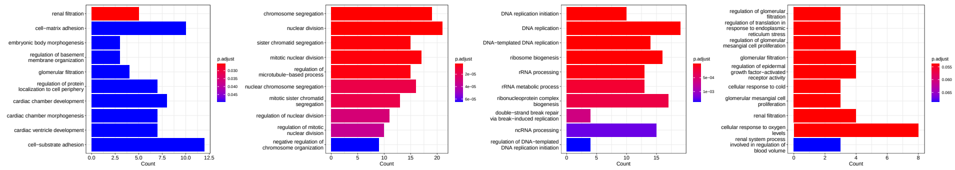

Lambda 0.5 Delta 100

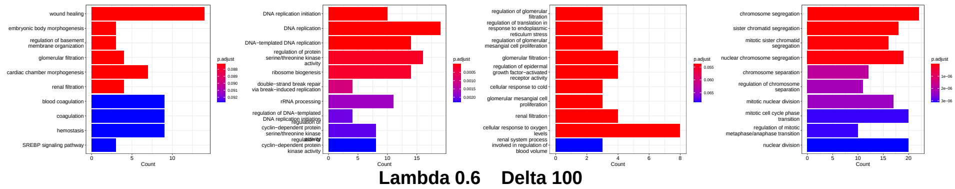

Lambda 0.6 Delta 100

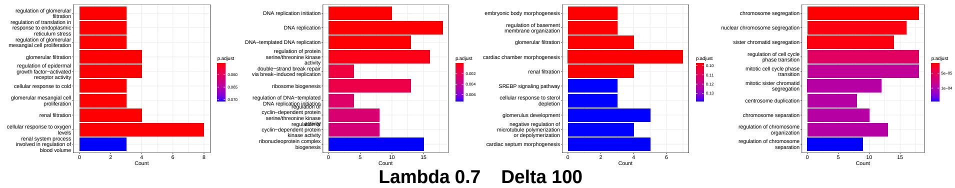

Lambda 0.7 Delta 100

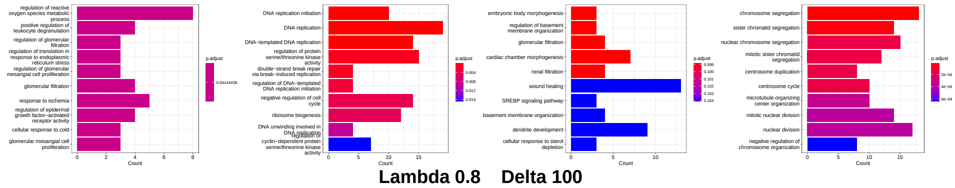

Lambda 0.8 Delta 100

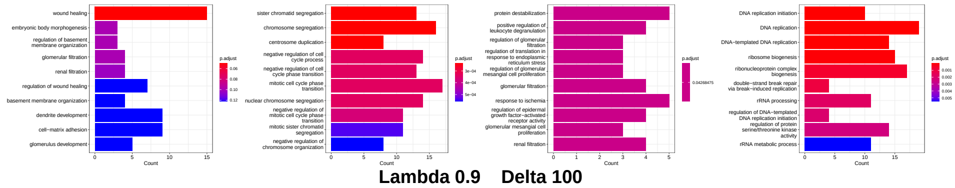

Lambda 0.9 Delta 100

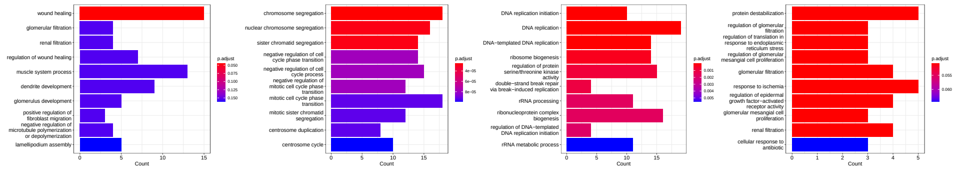

See next page

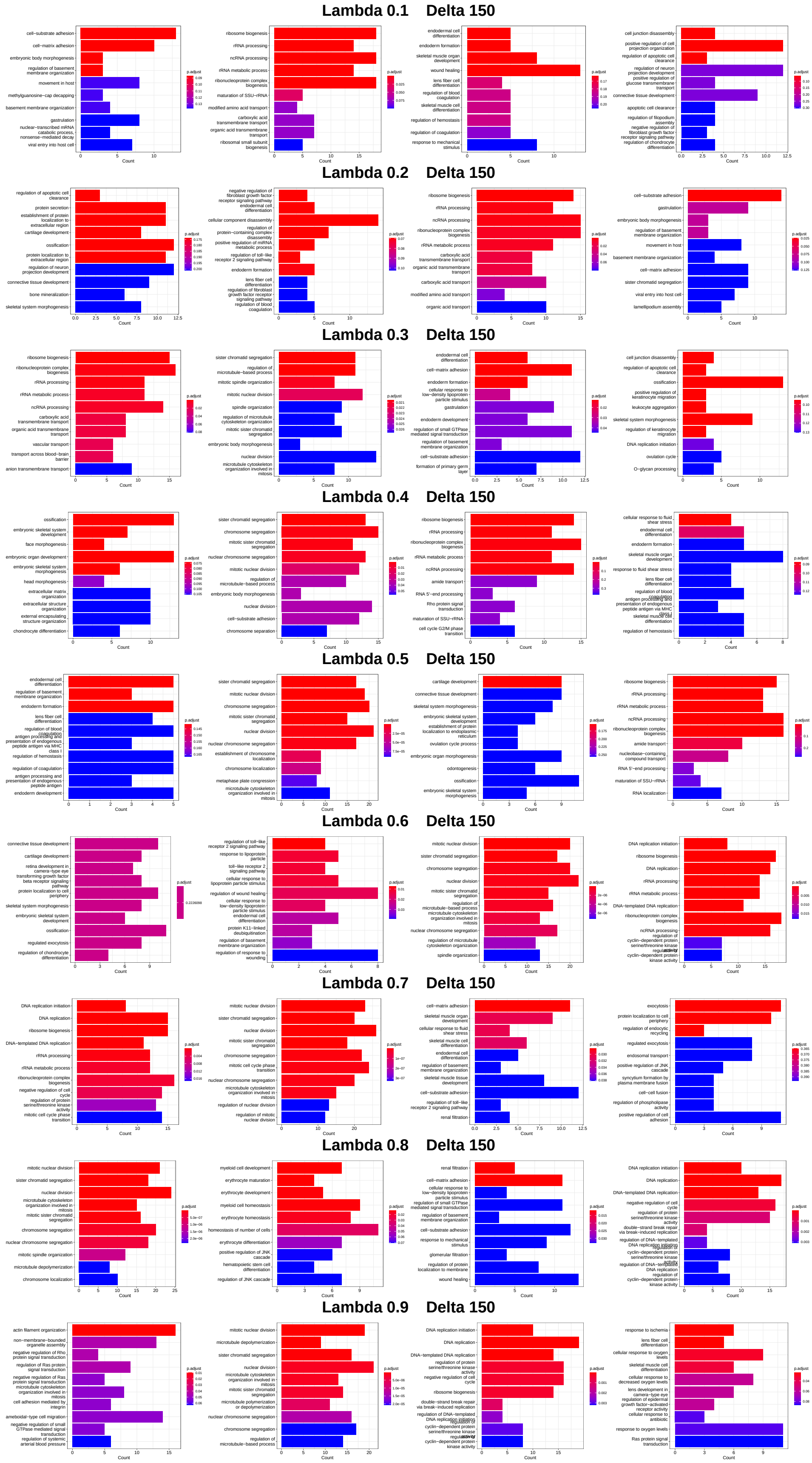

See next page

Lambda 0.1 Delta 200

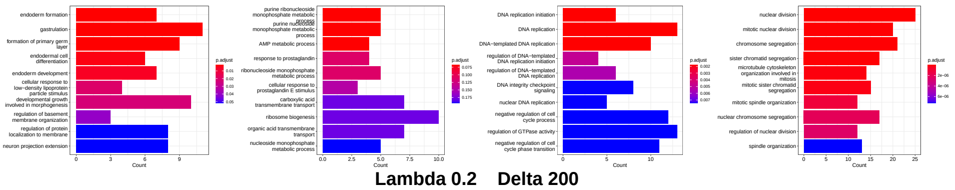

Lambda 0.2 Delta 200

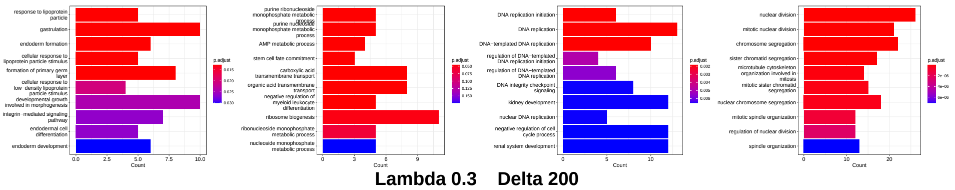

Lambda 0.3 Delta 200

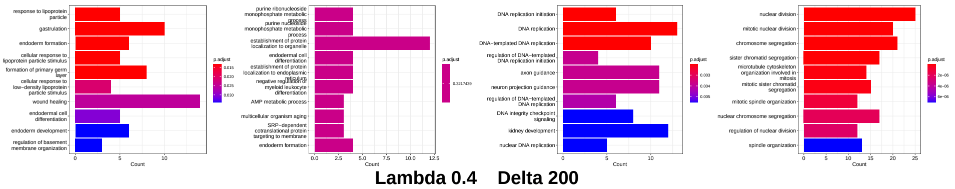

Lambda 0.4 Delta 200

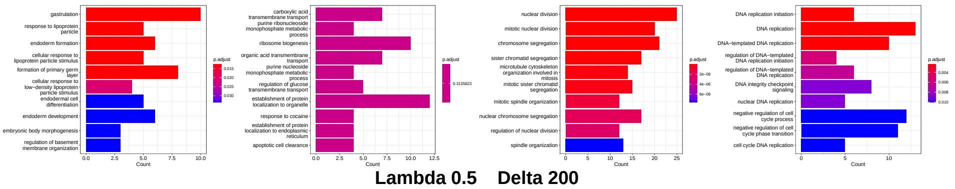

Lambda 0.5 Delta 200

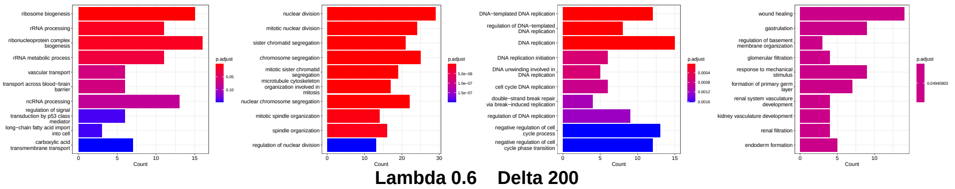

Lambda 0.6 Delta 200

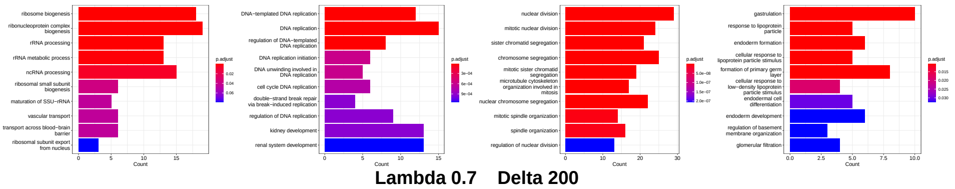

Lambda 0.7 Delta 200

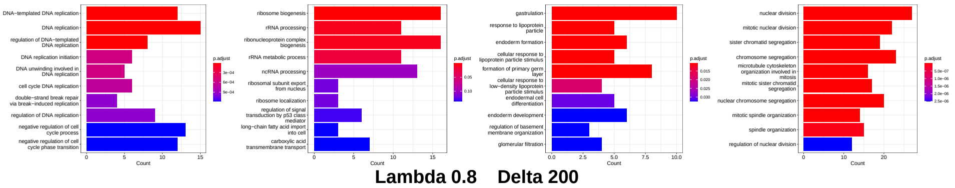

Lambda 0.8 Delta 200

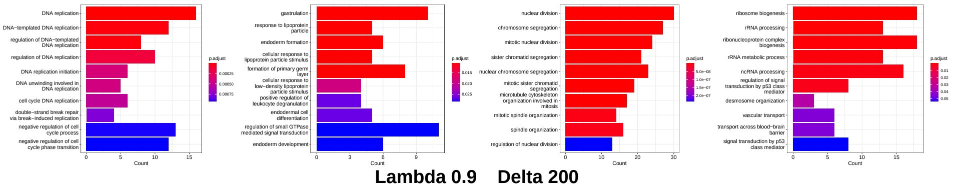

Lambda 0.9 Delta 200

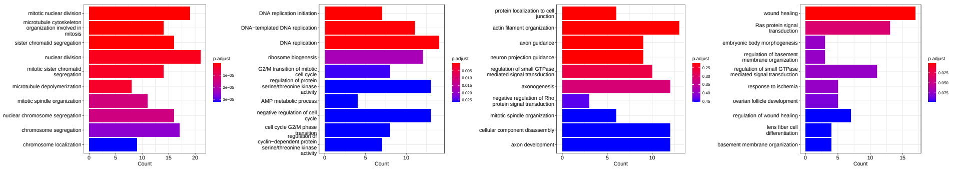

See next page

Lambda 0.1 Delta 250

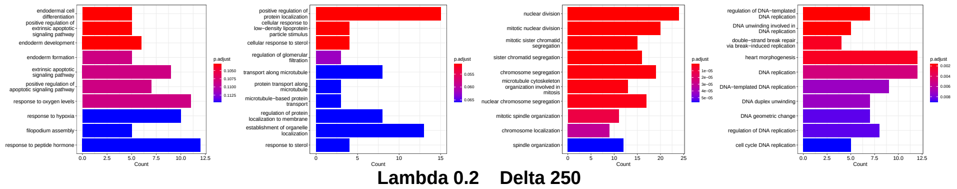

Lambda 0.2 Delta 250

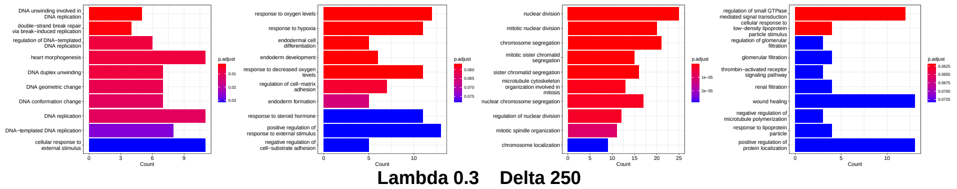

Lambda 0.3 Delta 250

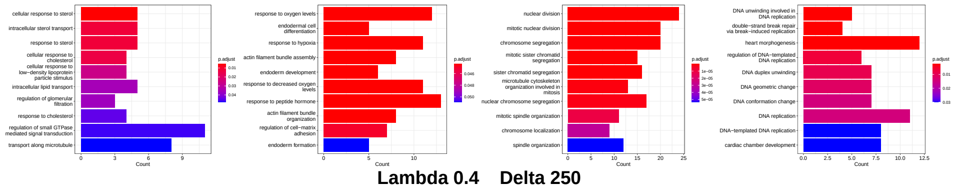

Lambda 0.4 Delta 250

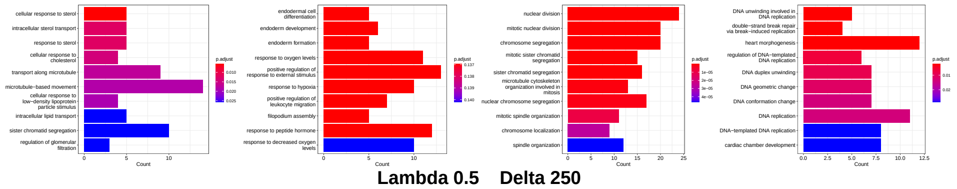

Lambda 0.5 Delta 250

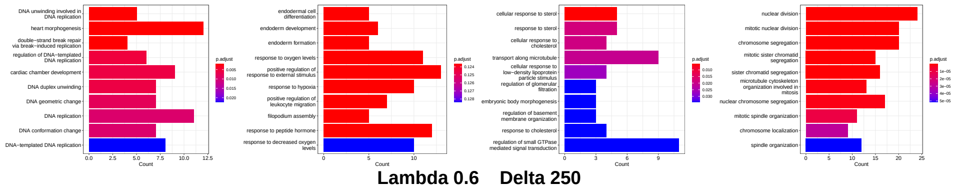

Lambda 0.6 Delta 250

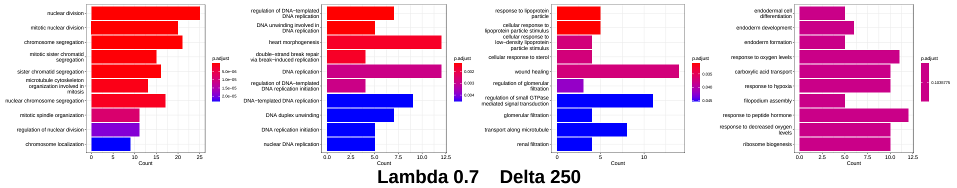

Lambda 0.7 Delta 250

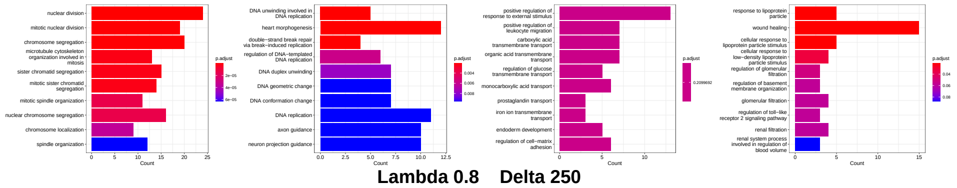

Lambda 0.8 Delta 250

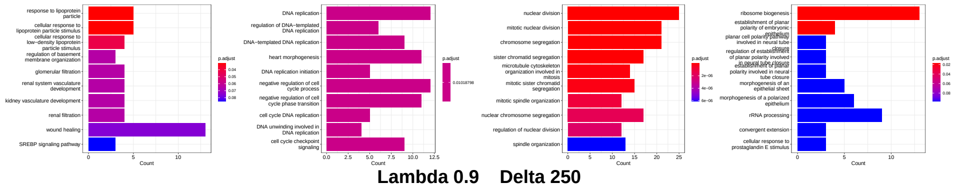

Lambda 0.9 Delta 250

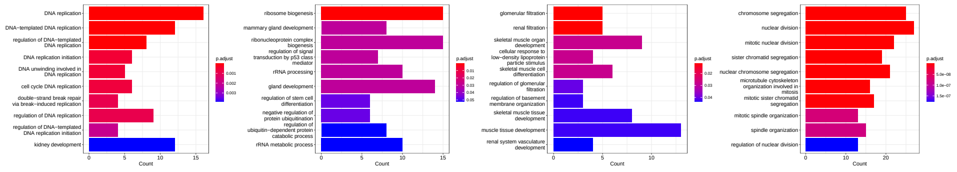

See next page

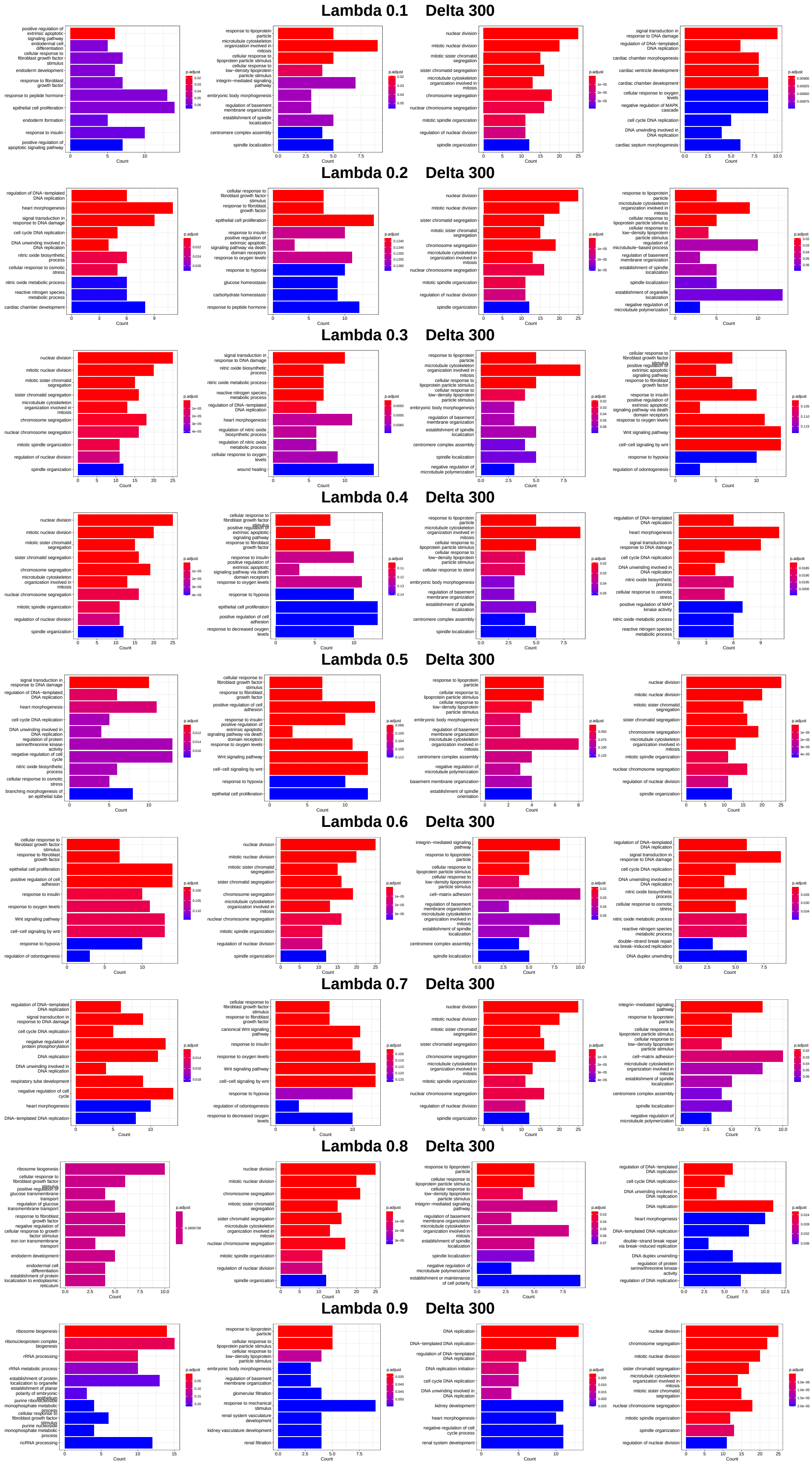

See next page

Lambda 0.1 Delta 350

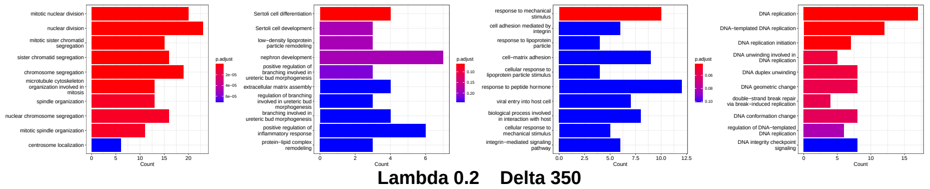

Lambda 0.2 Delta 350

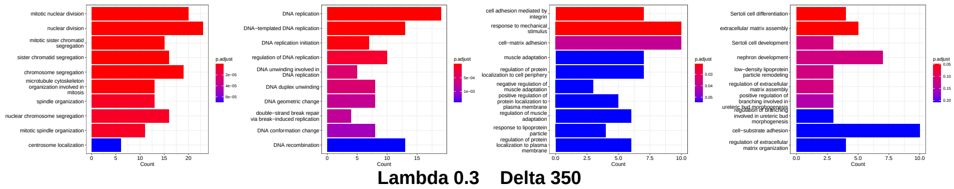

Lambda 0.3 Delta 350

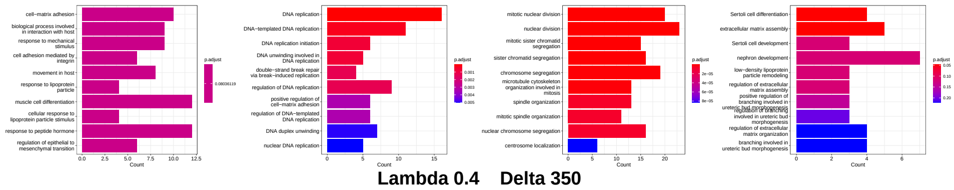

Lambda 0.4 Delta 350

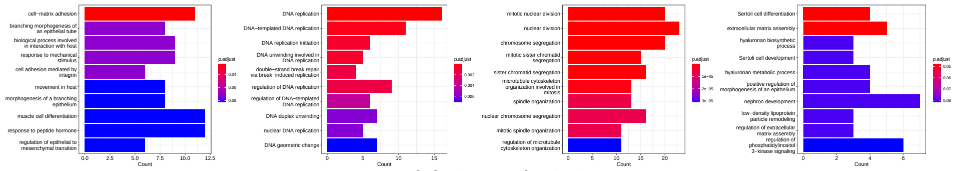

Lambda 0.5 Delta 350

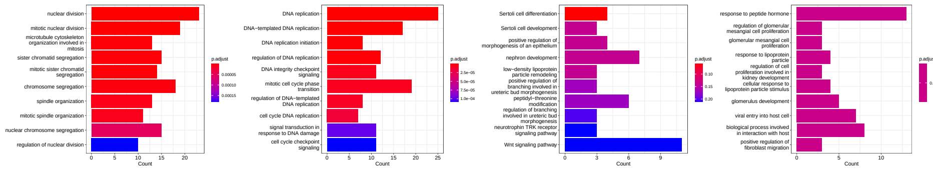

Lambda 0.6 Delta 350

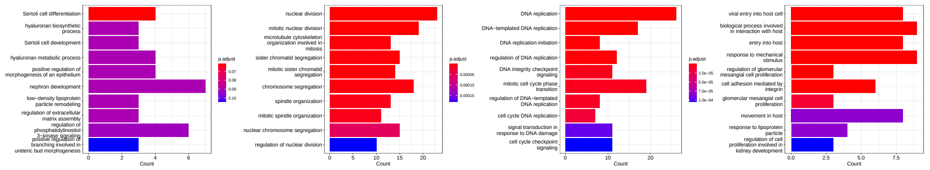

Lambda 0.7 Delta 350

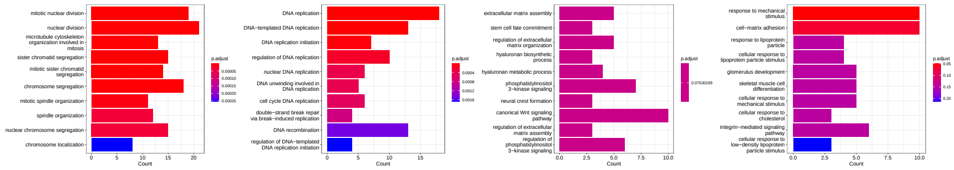

Lambda 0.8 Delta 350

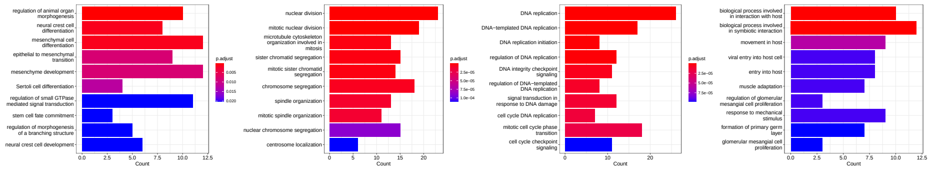

Lambda 0.9 Delta 350

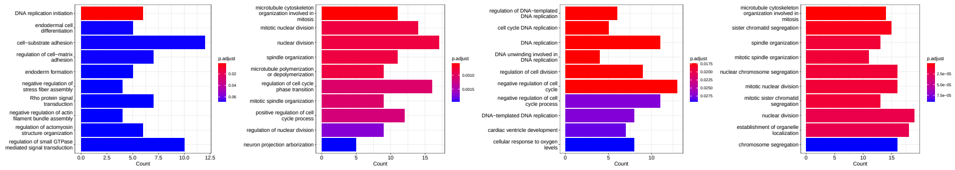

See next page

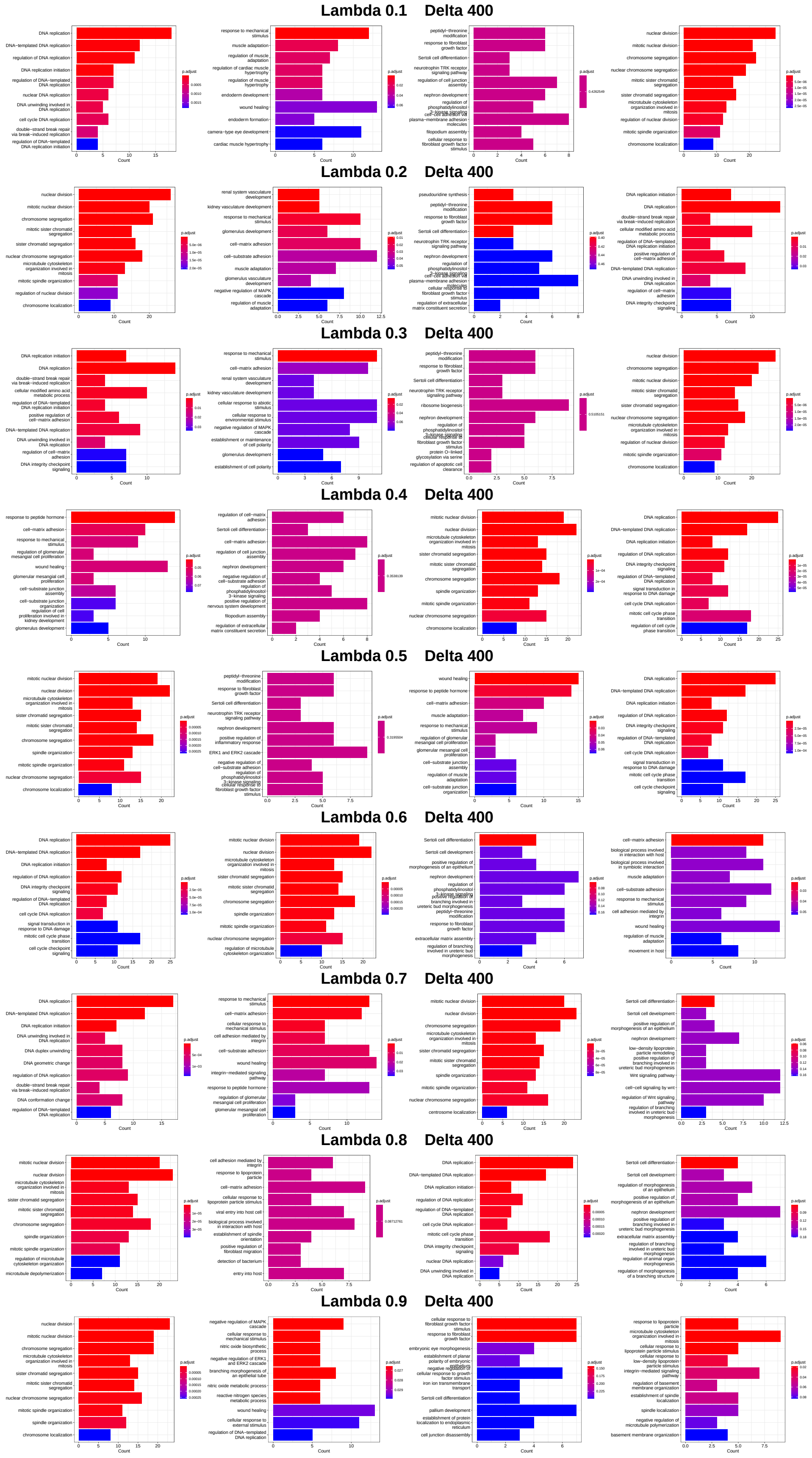

Figure S23: GO term enrichment analysis of stDGCC with different combinations of hyperparameters on the MERFISH human osteosarcoma dataset. The GO terms belong to C1, C2, C3, and C4 from left to right.

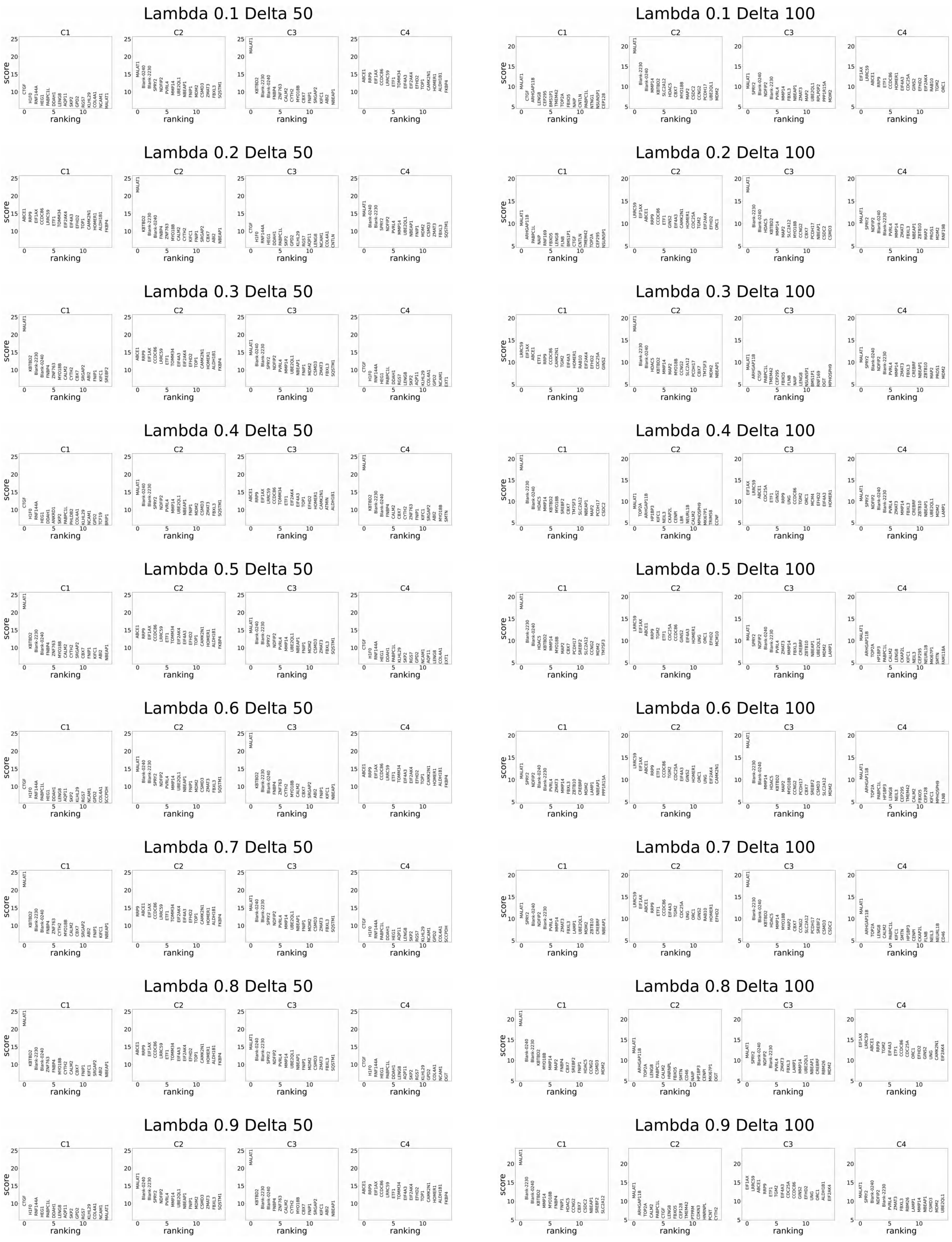

See next page

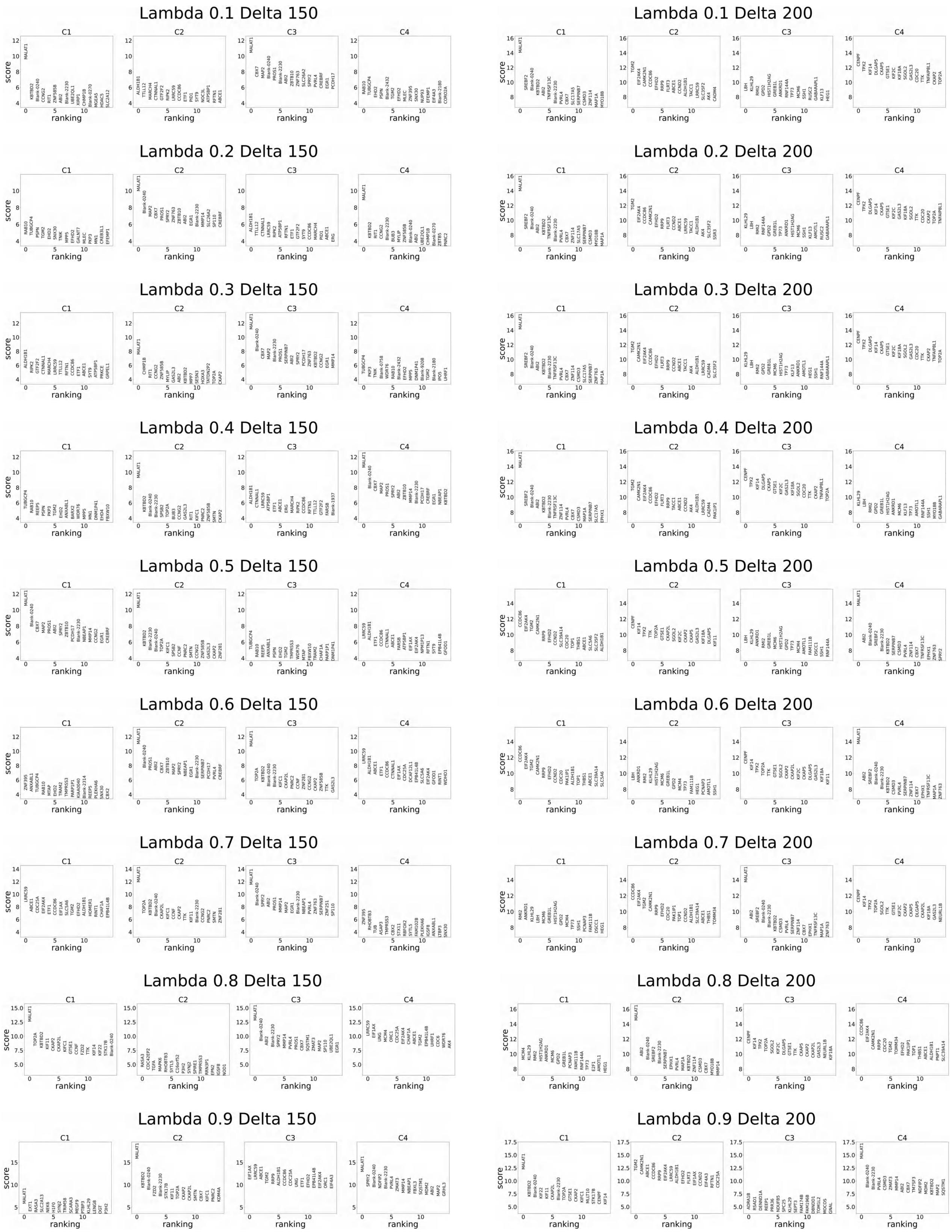

See next page

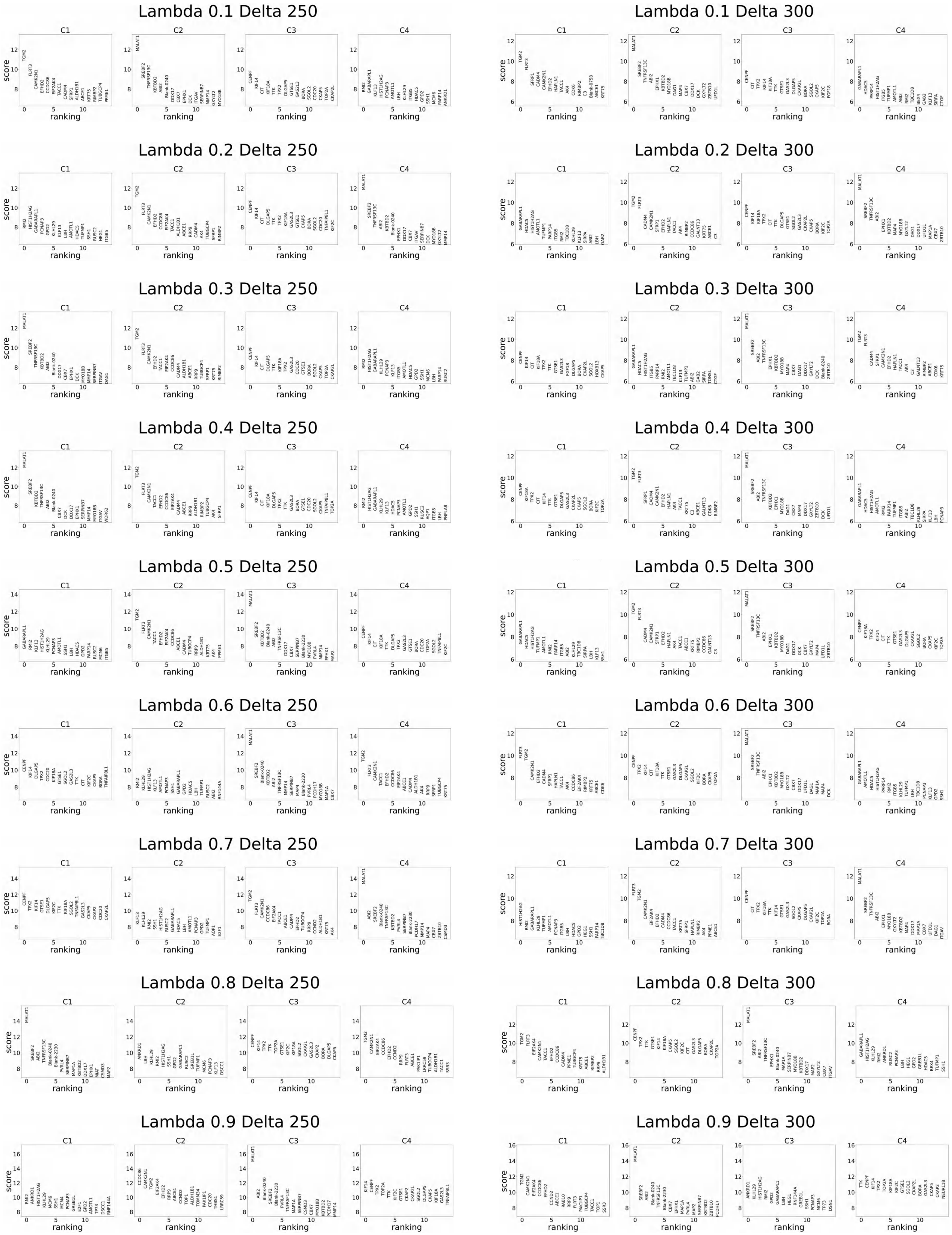

See next page

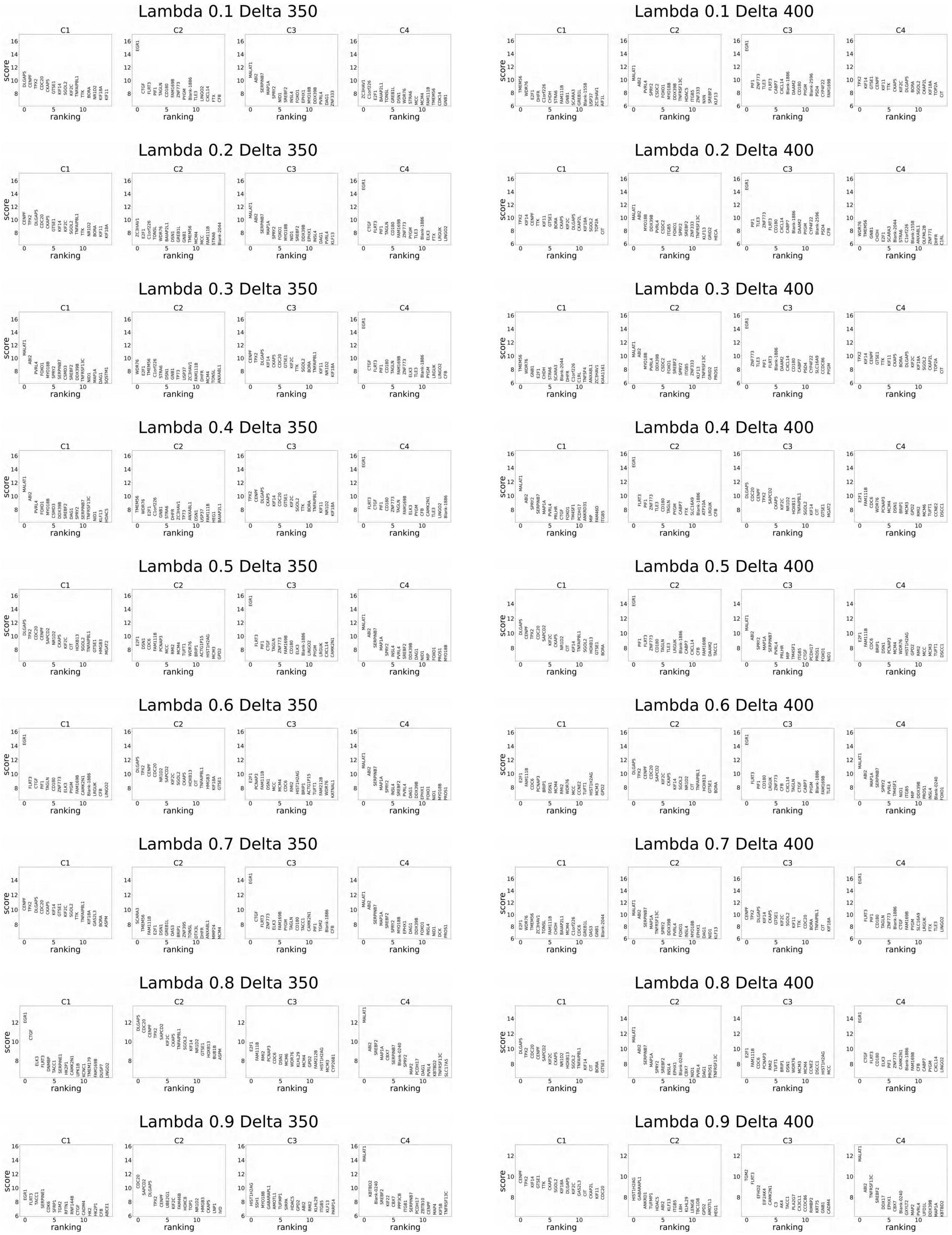

Figure S24: Top 15 DE genes of C1, C2, C3, and C4 of stDGCC with different combinations of hyperparameters on the MERFISH human osteosarcoma dataset

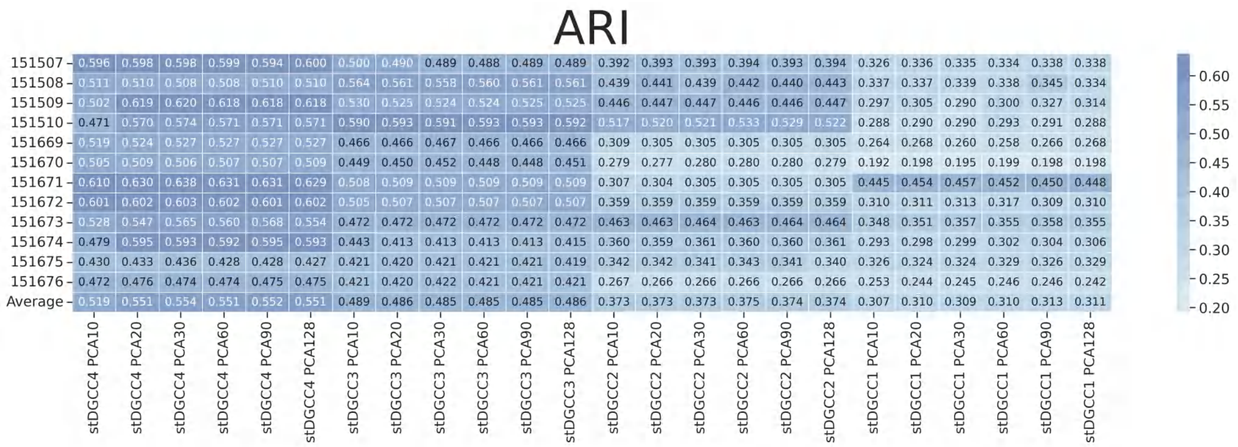

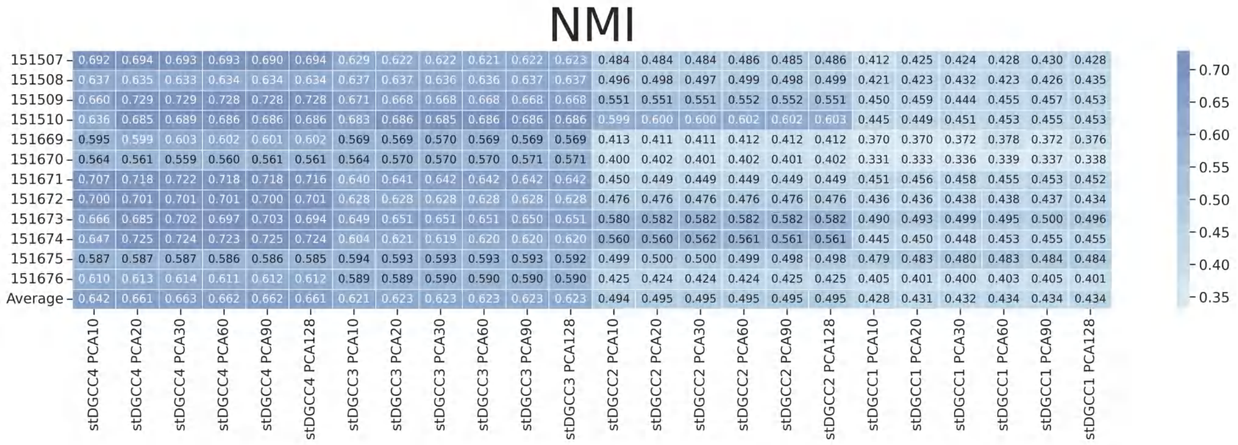

Figure S25: The clustering performance of stDGCC with different numbers of principal components, layers and nodes on the DLPFC dataset was evaluated using NMI and ARI as evaluation metrics.

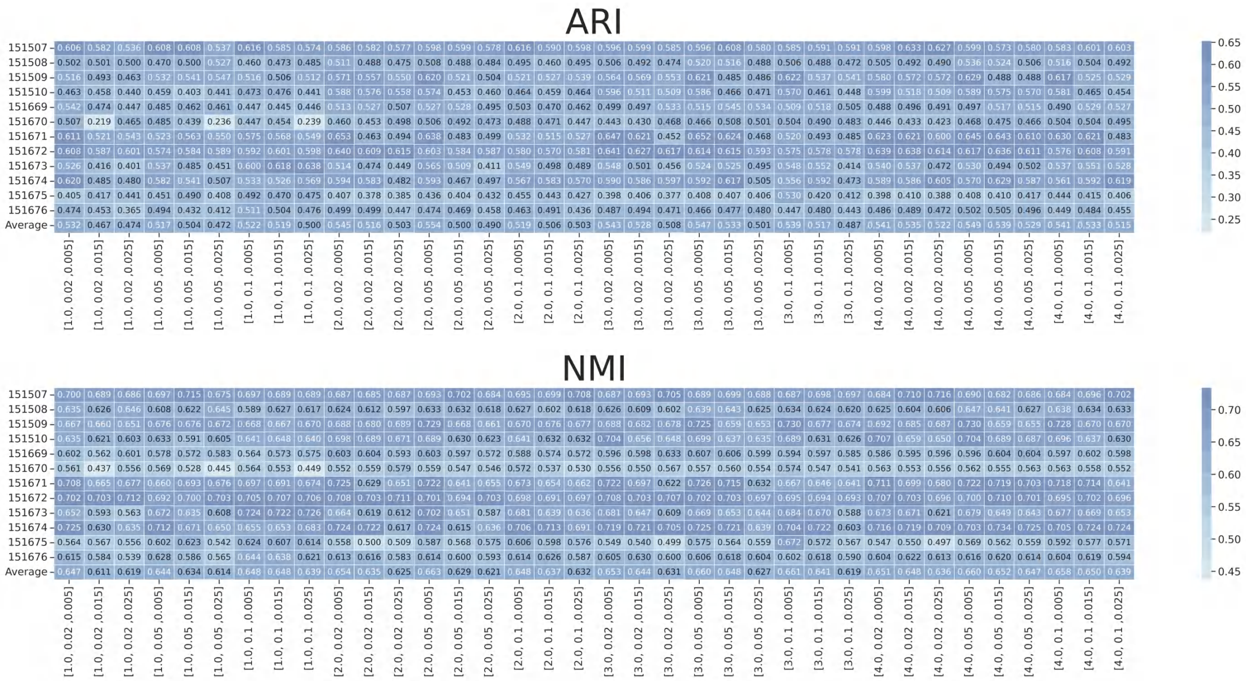

Figure S26: The clustering performance of stDGCC with different loss weight assignments on the DLPFC dataset was evaluated using NMI and ARI as evaluation metrics.

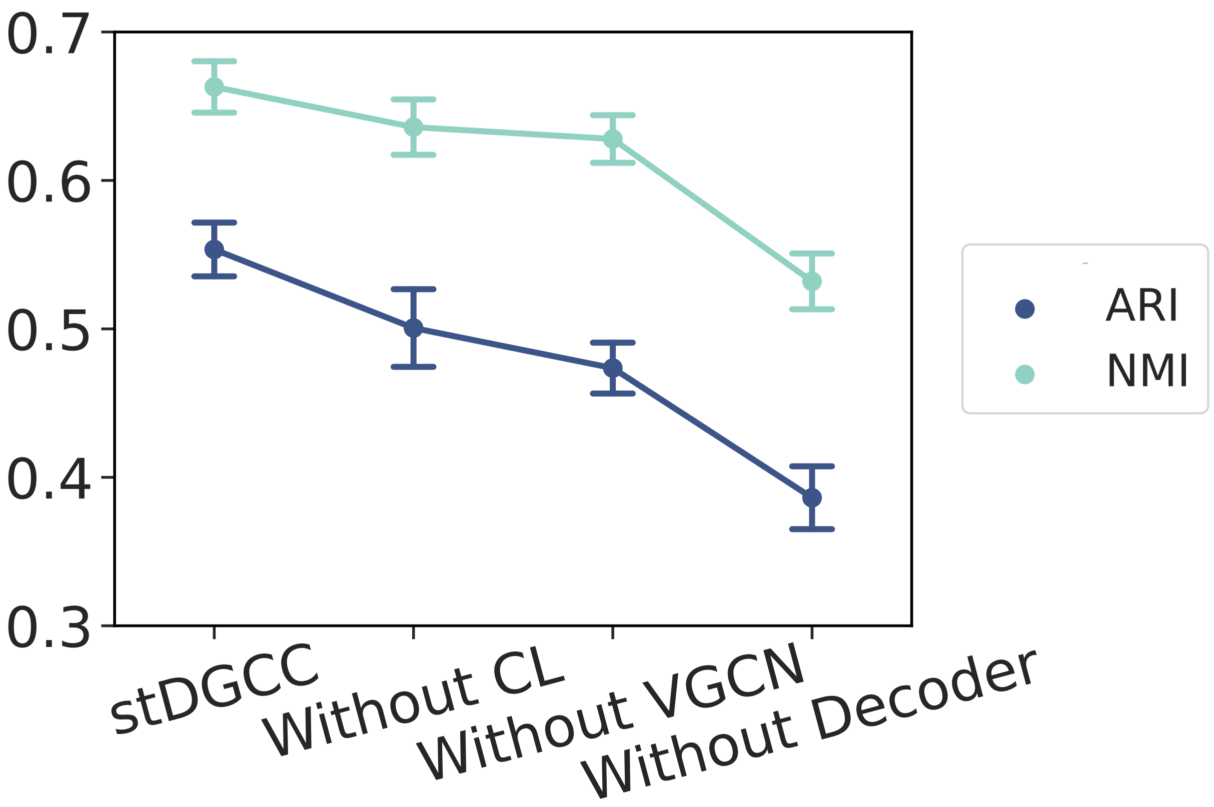

Figure S27: Line plot for clustering performance of stDGCC in four different cases on the DLPFC dataset.

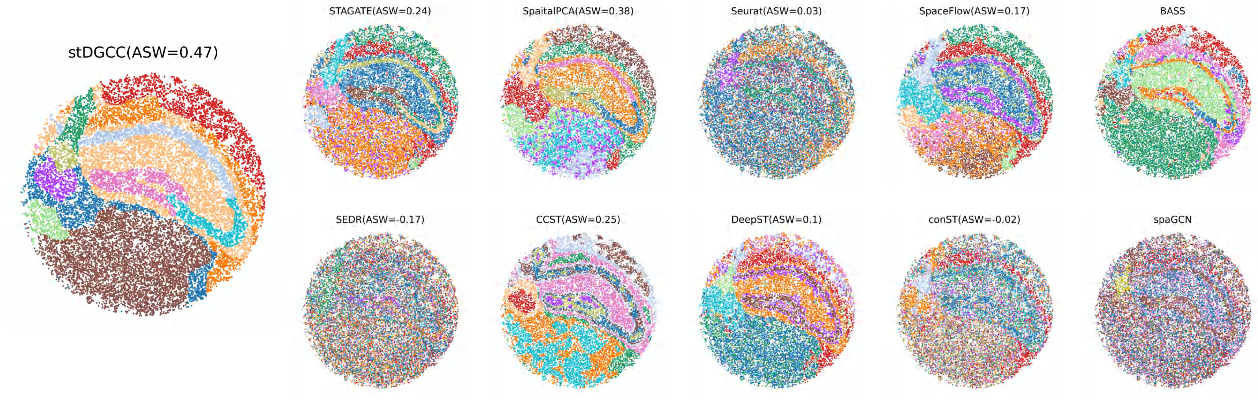

Figure S28: Spatial domains generated by stDGCC and other baseline methods on the Slide-seqV2 hippocampus dataset. Note that SpaGCN being end-to-end clustering method, cannot cannot be calculated with ASW.

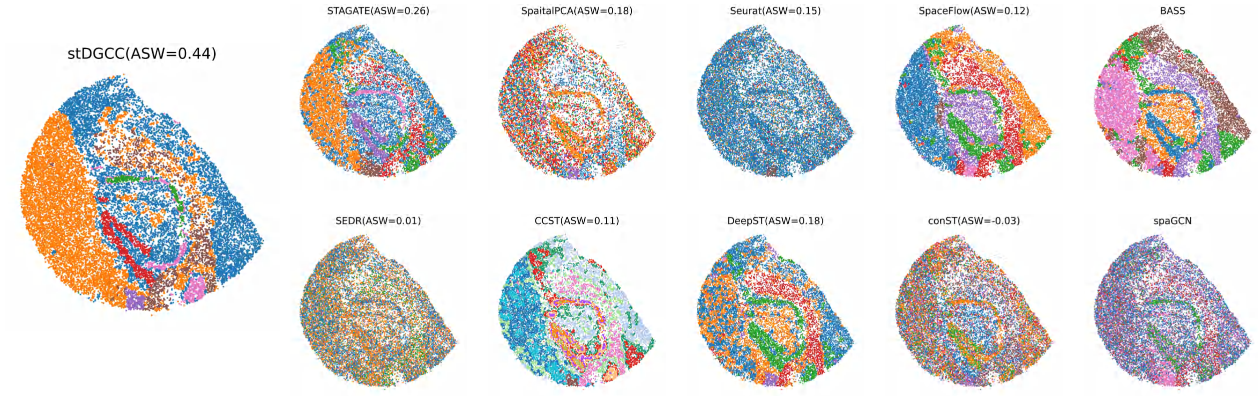

Figure S29: Spatial domains generated by stDGCC and other baseline methods on the Slide-seq hippocampus dataset. Note that SpaGCN and BASS being end-to-end clustering methods, cannot be calculated with ASW.

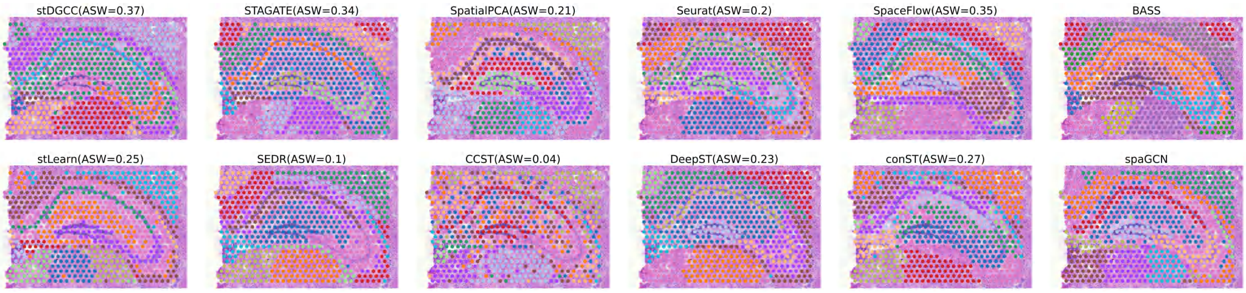

Figure S30: Spatial domains generated by stDGCC and other baseline methods on the 10x Visium mouse brain dataset. Note that SpaGCN and BASS being end-to-end clustering methods, cannot be calculated with ASW.

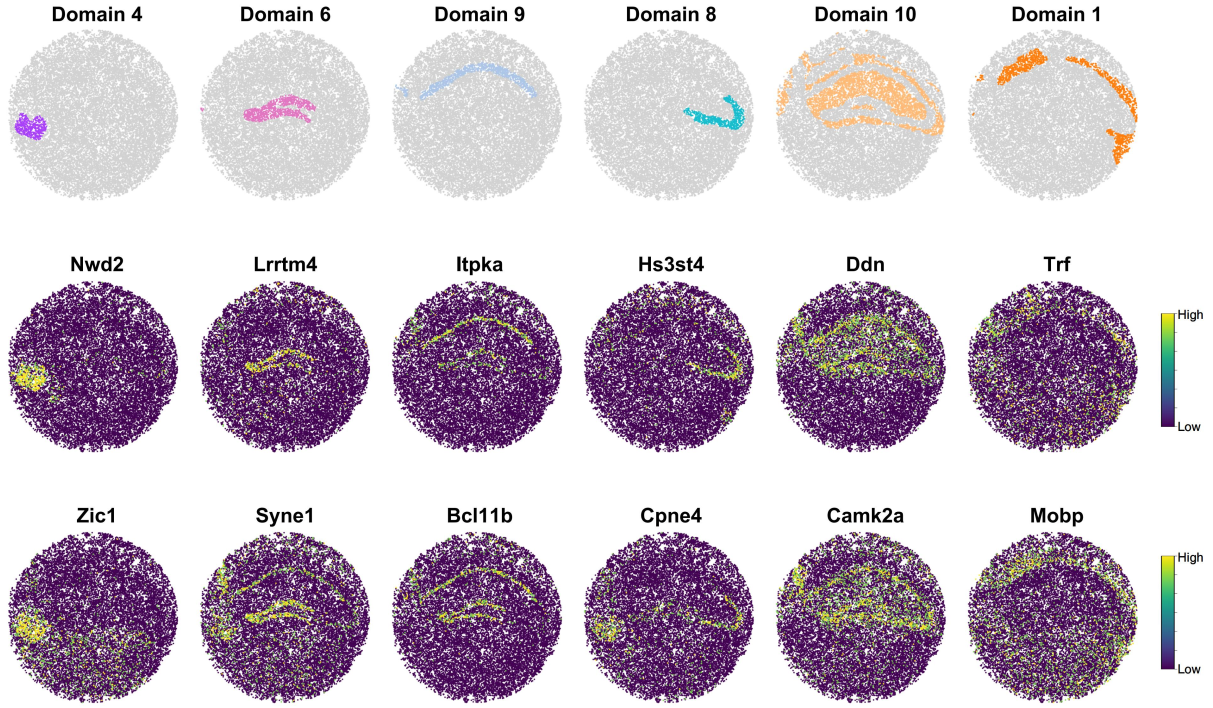

Figure S31: Visualization of marker genes and corresponding domains delineated by stDGCC on the Slide-seqV2 mouse hippocampus dataset.

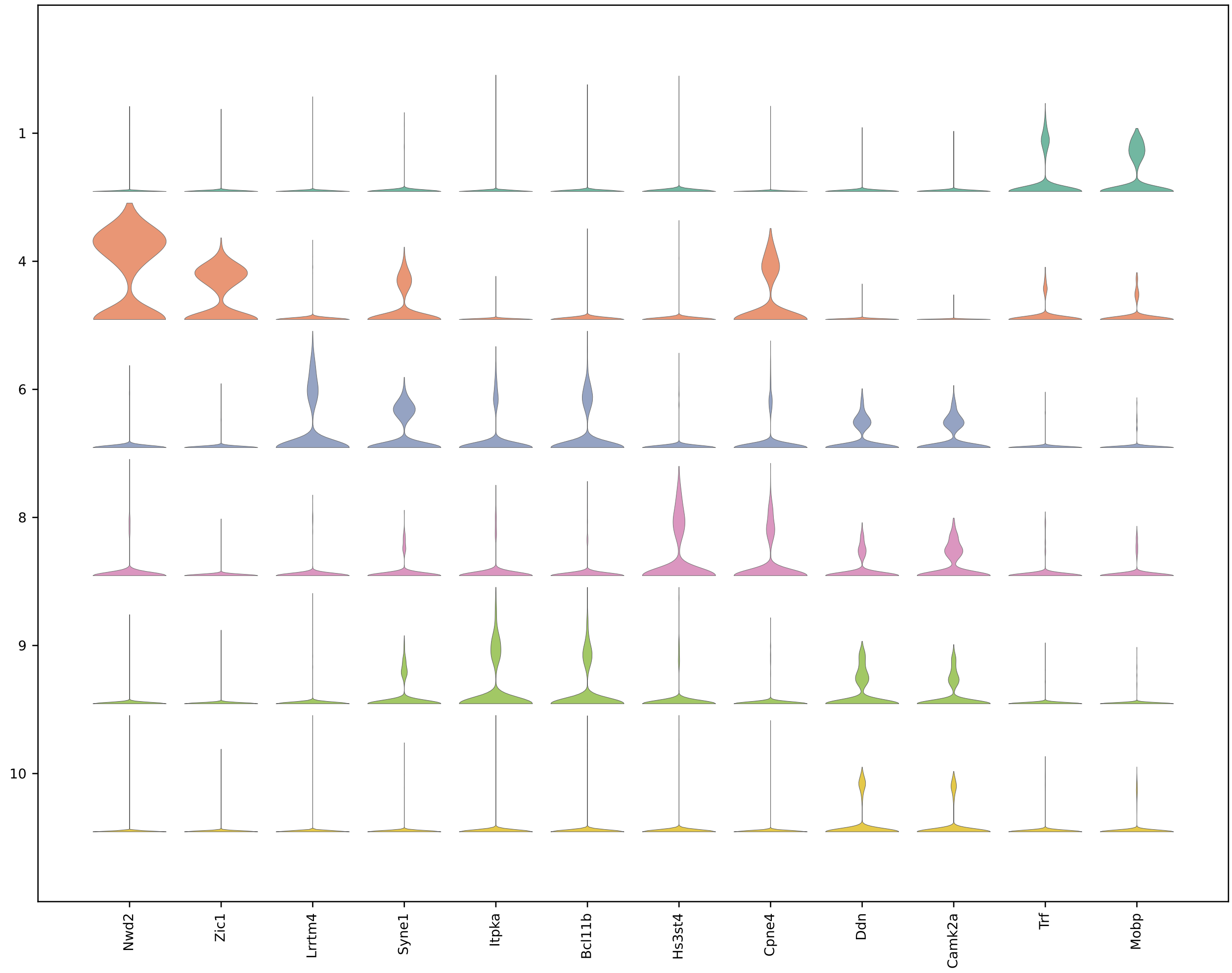

Figure S32: Stacked violin plots of marker genes in domains (1, 4, 6, 8, 9, 10) delineated by stDGCC on the Slide-seqV2 mouse hippocampus dataset.

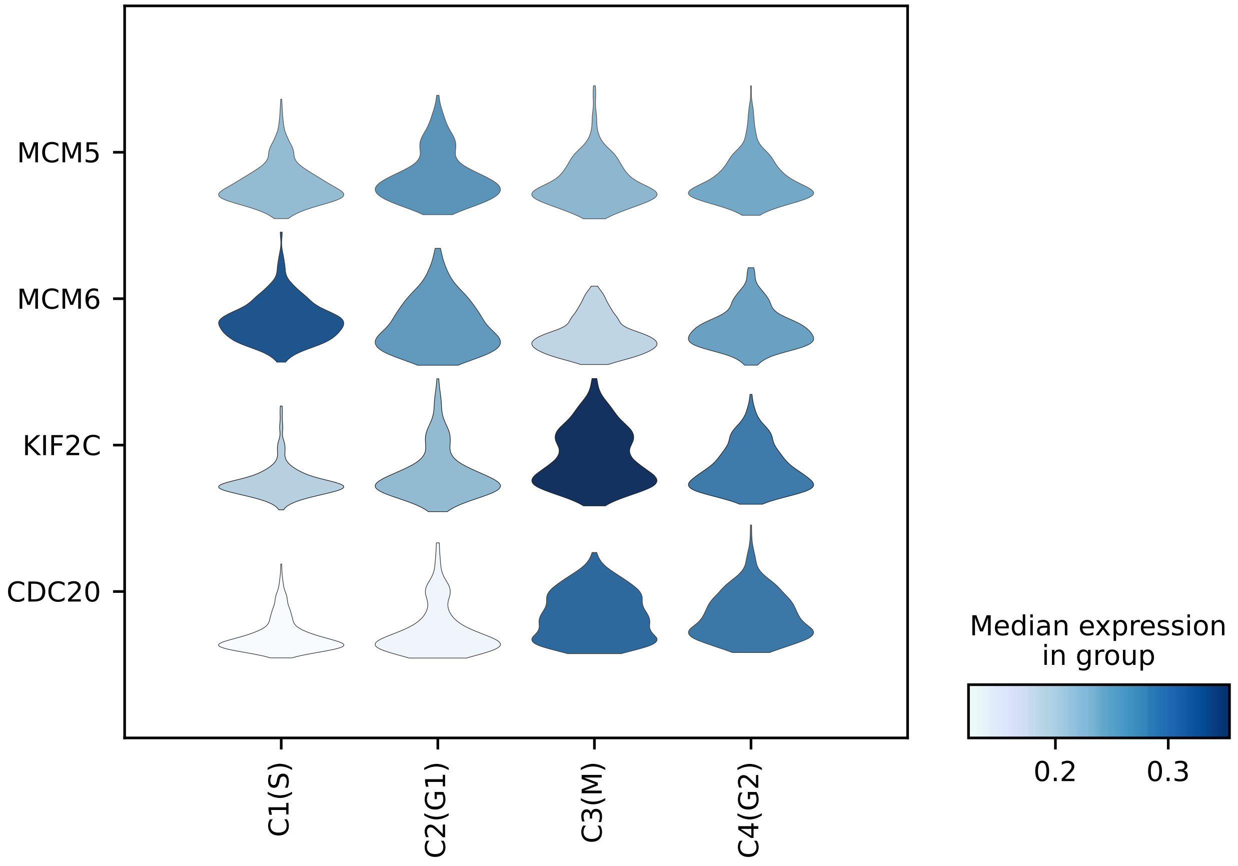

Figure S33: Stacked violin plots of marker genes in each cluster for stDGCC on the MERFISH human osteosarcoma dataset.

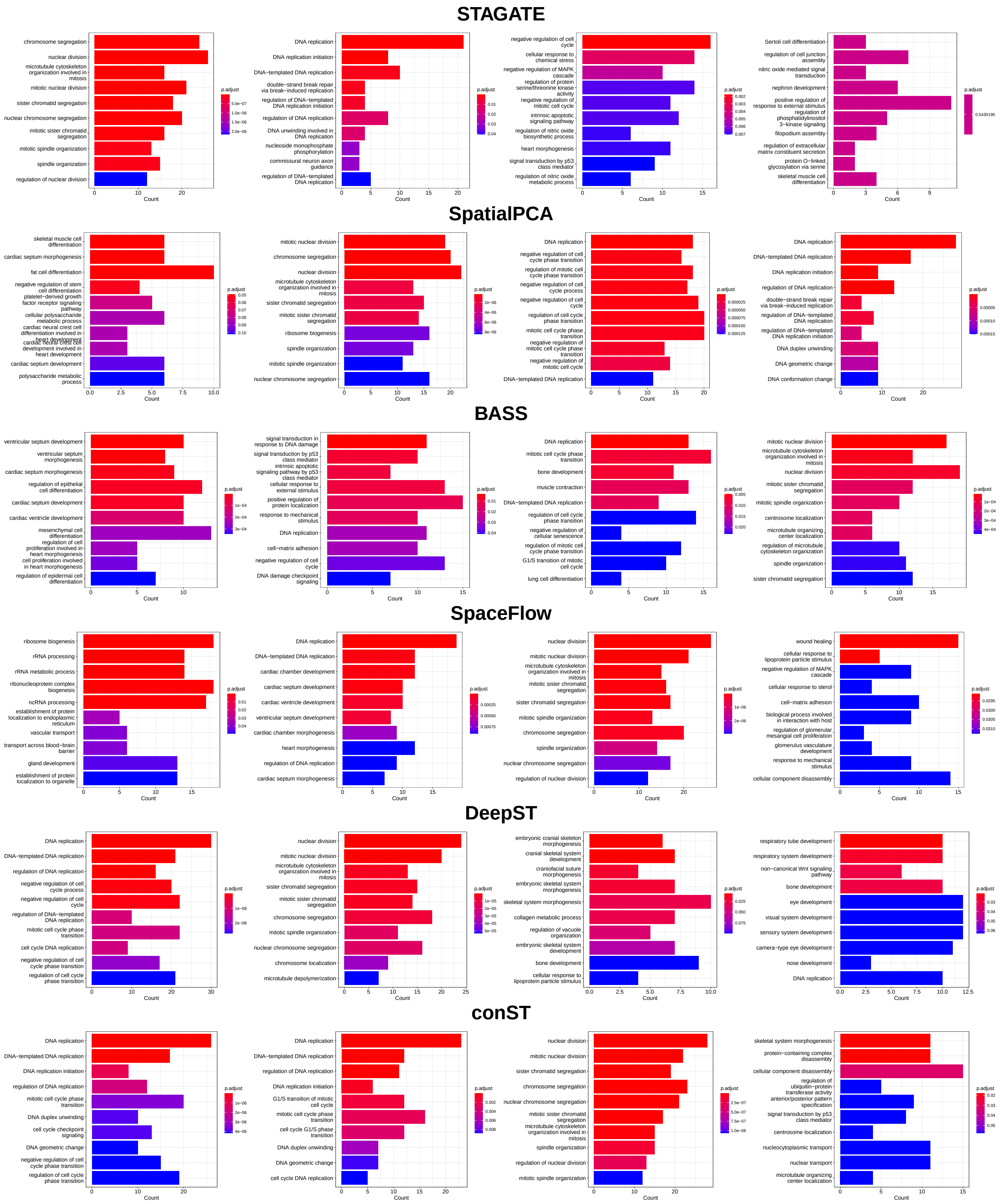

See next page

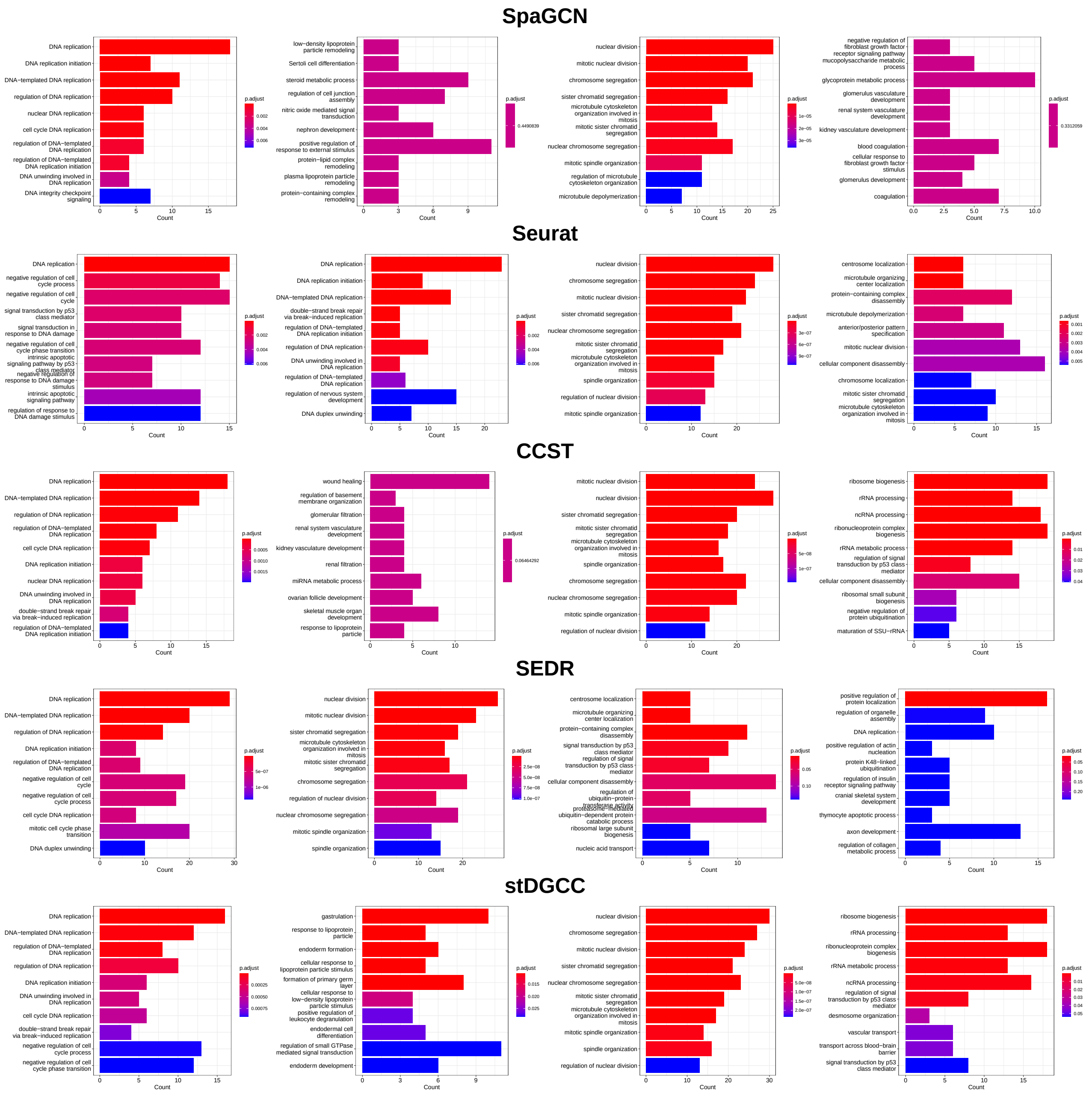

Figure S34: GO term enrichment analysis of stDGCC and other baseline methods on the MERFISH human osteosarcoma dataset. The GO terms belong to C1, C2, C3, and C4 from left to right.

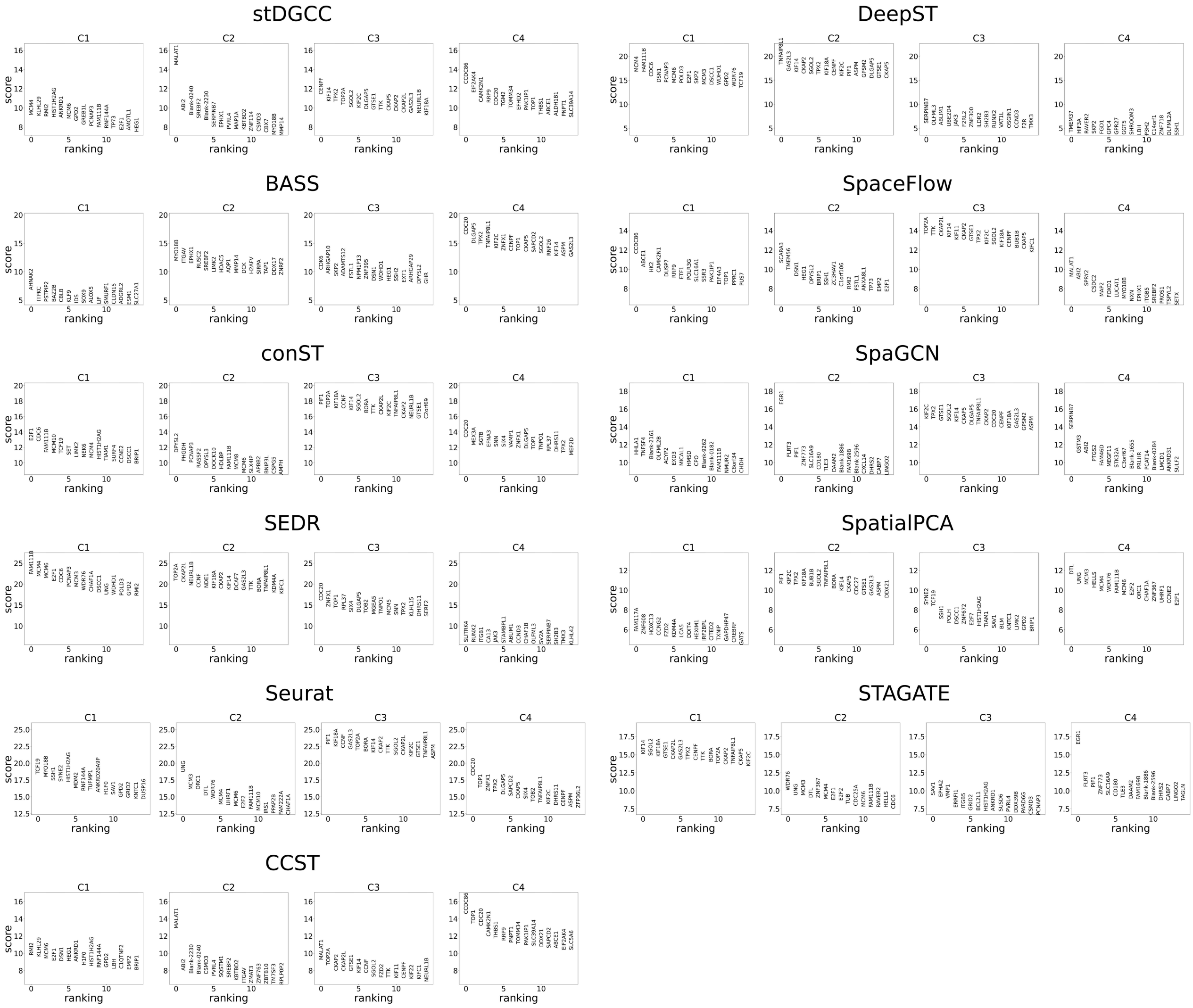

Figure S35: Top 15 DE genes of C1, C2, C3, and C4 of stDGCC and other baseline methods on the MERFISH human osteosarcoma dataset.

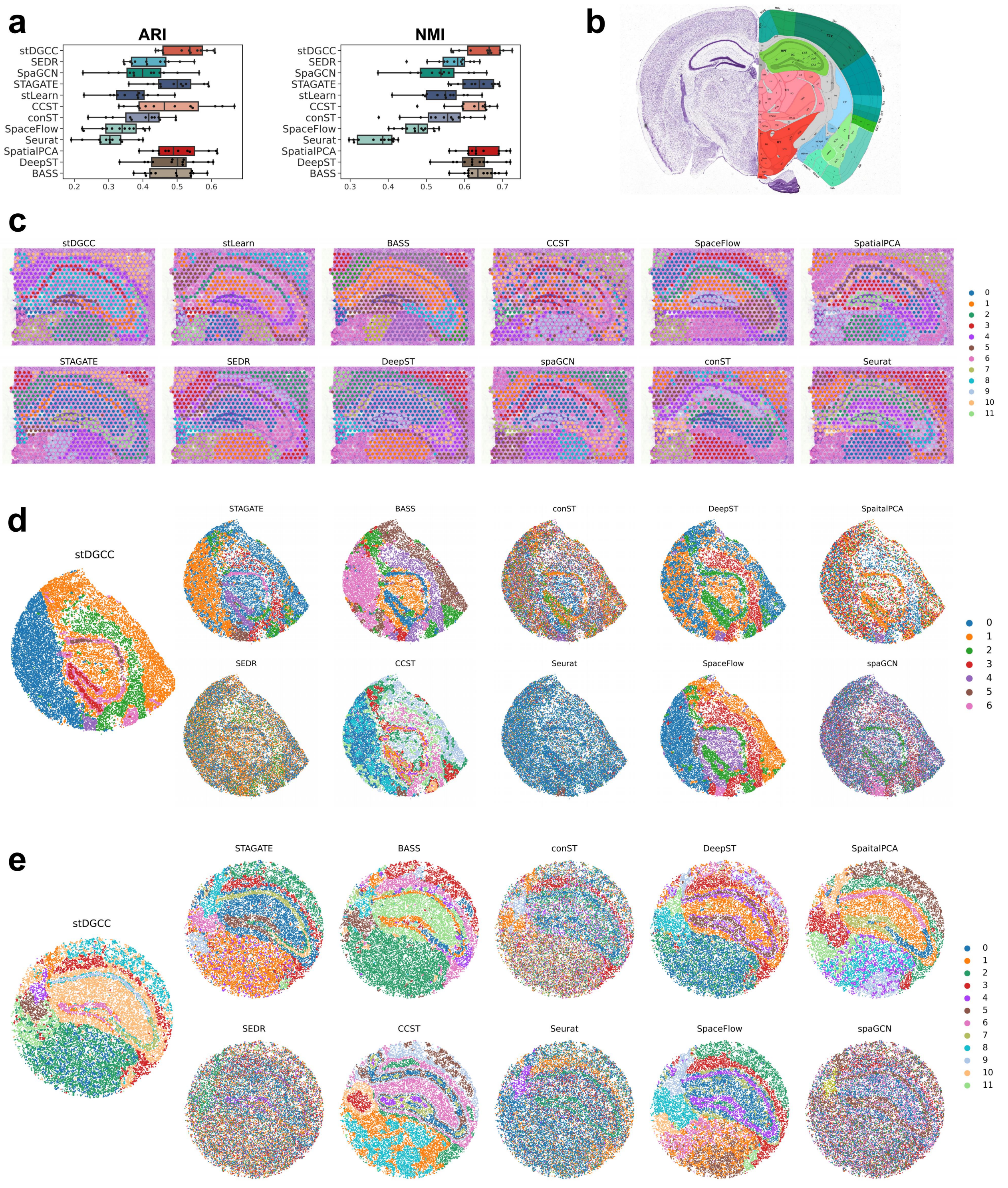

Figure S36: (a) Box plots of ARI and NMI for stDGCC and other baseline methods on the DLPFC dataset. (b) Allen mouse brain reference atlas. (c) Spatial domains generated by stDGCC and other baseline methods on the 10x mouse brain dataset. (d) Spatial domains generated by stDGCC and other baseline methods on the Slide-seq mouse hippocampus dataset. (e) Spatial domains generated by stDGCC and other baseline methods on the Slide-seqV2 mouse hippocampus dataset.
